# Supplementary material for: Comparing proficiency of obstetrics and gynaecology trainees with general surgery trainees using simulated laparoscopic tasks in Health Education England, North-West: a prospective observational study
Source: BMJ Open. 2023 Nov 10;13(11):e075113. doi: 10.1136/bmjopen-2023-075113 (PMC10649792; doi:10.1136/bmjopen-2023-075113)
Supplement: Supplementary data [file bmjopen-2023-075113supp001.pdf]

Table S1: Training matrix for O&G (adapted from RCOG). Required laparoscopic competencies are highlighted in **bold**. Competencies are signed off based on an entrustability scale* and as such no indicative numbers are included here.

	ST1	ST2	ST3	ST4	ST5	ST6	ST7
Curriculum	CiP progress appropriate to the relevant stage as per the CiP guides and entrustability levels.						
Examination	MRCOG Part 1			MRCOG Part 2 & Part 3			
At least 3 summative OSATS confirming competency by more than one assessor. At least one OSAT confirming competence should be supervised by a consultant.	Cervical smear	Caesarean section (Basic)<br><br>Non rotational assisted vaginal delivery (Ventouse & forceps)<br><br>Perineal repair<br><br>Surgical management of miscarriage/Surgical termination of pregnancy<br><br>Insertion of an Intrauterine system/intrauterine contraceptive device.<br><br>Endometrial biopsy	Manual removal of placenta<br><br>Transabdominal USS of early and late pregnancy	Hysteroscopy<br><br>**Diagnostic laparoscopy**<br><br>3rd degree perineal repair<br><br>Vulval biopsy	**Simple operative laparoscopy** (laparoscopic sterilisation or simple adnexal surgery e.g. adhesiolysis/ ovarian drilling<br><br>Caesarean section (Intermediate)<br><br>Rotational assisted vaginal delivery (any method)		Subspecialty specific competencies.<br><br>**Laparoscopic management of ectopic pregnancy**<br><br>**Ovarian cystectomy (laparoscopic & open)**<br><br>Surgical management of post partum haemorrhage

*Entrustability scale: 1= observe only, 2= direct supervision, 3= indirect supervision, 4= act independently with support, 5= act independently.
