# Supplementary material for: Comparing proficiency of obstetrics and gynaecology trainees with general surgery trainees using simulated laparoscopic tasks in Health Education England, North-West: a prospective observational study
Source: BMJ Open. 2023 Nov 10;13(11):e075113. doi: 10.1136/bmjopen-2023-075113 (PMC10649792; doi:10.1136/bmjopen-2023-075113)
Supplement: Supplementary data [file bmjopen-2023-075113supp002.pdf]

Table S2: Summary of required procedures in GS training. Indicative case/operative numbers are given for the specialty training phase where both the numbers and entrustability scales are used for assessment.

	CT1 Phase 1	CT2 Phase 1	ST3 Phase 2	ST4 Phase 2	ST5 Phase 2	ST6 Phase 2	ST7 Phase 3	ST8 Phase 3
Examinations		MRCS Part A<br>MRCS Part B						FRCS Part 1<br>FRCS Part 2
**Operative Requirements**<br><u>Level 1</u><br>Has observed<br><u>Level 2</u><br>Can do with assistance<br><u>Level 3</u><br>Can do whole but may need assistance<br><u>Level 4</u><br>Competent to do without assistance, including complications		Induction of pneumoperitoneum for laparoscopy with port placement (Level 2)<br>Appendicectomy (Level 3)<br>Open and close midline laparotomy incision (2)<br>Inguinal hernia repair (Level 2)<br>Primary abdominal wall hernia repair (Level 2)				Inguinal Hernia (level 4) [50 cases*]<br>Cholecystectomy (level 3) [40 cases*]<br>Segmental Colectomy (level 3) [15 cases*]<br>Emergency Laparotomy [45* cases]<br>Appendicectomy [60 cases*]		Emergency Laparotomy (Level 4) [100 cases*]<br>Appendicectomy (Level 4) [80 cases*]<br>Cholecystectomy [50 cases*] (level 4)<br>Segmental colectomy [20 cases*] (level 4)
**Other Operative Technical Skills**		Chest drain insertion (Level 3)<br>Needle biopsy including fine needle aspiration (Level 3)<br>Rigid sigmoidoscopy (Level 3)<br>Excision biopsy of benign skin or subcutaneous lesion (Level 4)						Indicative numbers and competencies for chosen specialty required. (Hepatopancreaticobiliary, Transplant, Endocrine, Colorectal, Oesophagogastric)
