# Supplementary material for: Comparing proficiency of obstetrics and gynaecology trainees with general surgery trainees using simulated laparoscopic tasks in Health Education England, North-West: a prospective observational study
Source: BMJ Open. 2023 Nov 10;13(11):e075113. doi: 10.1136/bmjopen-2023-075113 (PMC10649792; doi:10.1136/bmjopen-2023-075113)
Supplement: Author's manuscript [file bmjopen-2023-075113.draft_revisions.pdf]

BMJ Open is committed to open peer review. As part of this commitment we make the peer review history of every article we publish publicly available.

When an article is published we post the peer reviewers' comments and the authors' responses online. We also post the versions of the paper that were used during peer review. These are the versions that the peer review comments apply to.

The versions of the paper that follow are the versions that were submitted during the peer review process. They are not the versions of record or the final published versions. They should not be cited or distributed as the published version of this manuscript.

BMJ Open is an open access journal and the full, final, typeset and author-corrected version of record of the manuscript is available on our site with no access controls, subscription charges or pay-per-view fees (http://bmjopen.bmj.com).

If you have any questions on BMJ Open's open peer review process please email info.bmjopen@bmj.com

BMJ Open

Performance in laparoscopic surgery is superior in general surgery trainees than obstetrics and gynaecology trainees: a prospective observational study

Journal:	*BMJ Open*
Manuscript ID	bmjopen-2023-075113
Article Type:	Original research
Date Submitted by the Author:	26-Apr-2023
Complete List of Authors:	Khan, Zaibun N; Royal Lancaster Infirmary<br>Shrestha, Donna; Lancaster University<br>Shugaba, Abdulwarith; Lancaster University<br>Lambert, Joel E; Lancaster University<br>Clark, T; Birmingham Women's NHS Foundation Trust, Gynaecology; University of Birmingham,<br>Haslett, Elizabeth; Blackpool Victoria Hospital<br>Afors, Karolina; Whittington Health NHS Trust<br>Bampouras, Theodoros M; School of Sport and Exercise Sciences<br>Gaffney, Christopher; Lancaster University<br>Subar, Daren; East Lancashire Hospitals NHS Trust
Keywords:	OBSTETRICS, GYNAECOLOGY, MEDICAL EDUCATION & TRAINING

SCHOLARONE™
Manuscripts

I, the Submitting Author has the right to grant and does grant on behalf of all authors of the Work (as defined in the below author licence), an exclusive licence and/or a non-exclusive licence for contributions from authors who are: i) UK Crown employees; ii) where BMJ has agreed a CC-BY licence shall apply, and/or iii) in accordance with the terms applicable for US Federal Government officers or employees acting as part of their official duties; on a worldwide, perpetual, irrevocable, royalty-free basis to BMJ Publishing Group Ltd ("BMJ") its licensees and where the relevant Journal is co-owned by BMJ to the co-owners of the Journal, to publish the Work in this journal and any other BMJ products and to exploit all rights, as set out in our licence.

The Submitting Author accepts and understands that any supply made under these terms is made by BMJ to the Submitting Author unless you are acting as an employee on behalf of your employer or a postgraduate student of an affiliated institution which is paying any applicable article publishing charge ("APC") for Open Access articles. Where the Submitting Author wishes to make the Work available on an Open Access basis (and intends to pay the relevant APC), the terms of reuse of such Open Access shall be governed by a Creative Commons licence – details of these licences and which Creative Commons licence will apply to this Work are set out in our licence referred to above.

Other than as permitted in any relevant BMJ Author's Self Archiving Policies, I confirm this Work has not been accepted for publication elsewhere, is not being considered for publication elsewhere and does not duplicate material already published. I confirm all authors consent to publication of this Work and authorise the granting of this licence.

For peer review only

Performance in laparoscopic surgery is superior in general surgery trainees than obstetrics and gynaecology trainees: a prospective observational study

Zaibun N Khan[1] (0000-0002-9558-8964), Donna Shrestha [2] (0000-0001-8711-3515), Abdulwarith Shugaba [2], Joel E Lambert [2] (0000-0001-5663-6284), T Justin Clark [3] (0000-0002-5943-1062), Elizabeth Haslett [4] (0000-0003-1165-0415), Karolina Afors [5], Theodoros M Bampouras [6] (0000-0002-8991-4655), Christopher J Gaffney [2]* (0000-0001-7990-2792), Daren A Subar [7].

Affiliations:

1. Royal Lancaster Infirmary, Ashton Rd, Lancaster, LA1 4RP, UK.

2. Lancaster Medical School, Lancaster University, Health Innovation One, Sir John Fisher Drive, Lancaster, LA1 4AT, UK.

3. Birmingham Women's Hospital, Birmingham Women's & Children's NHS Foundation Trust, Metchley Park Road, Edgbaston, Birmingham, B15 2TG, UK.

4. Blackpool Victoria Hospital, Whinney Heys Road, Blackpool, FY3 8NR.

5. Whittington Health NHS Trust, Magdala Avenue, London N19 5NF, UK.

6. Liverpool John Moores University, School of Sport and Exercise Sciences, Tom Reilly Building, Byrom Street, Liverpool, L3 3AF, UK.

7. East Lancashire Hospitals NHS Trust, Haslingden Road, Blackburn, BB2 3HH, UK.

Correspondence to: Dr Christopher J Gaffney, Lancaster Medical School, Lancaster University, Health Innovation One, Sir John Fisher Drive, Lancaster, LA1 4AT, UK; c.gaffney@lancaster.ac.uk; +44 (0) 1524 593 602

Word count: 4620

ABSTRACT

BACKGROUND: Training programmes for obstetrics and gynaecology (O&G) and general surgery (GS) vary significantly, but both require proficiency in laparoscopic skills. We sought to determine performance in each specialty.

DESIGN: Prospective, Observational study.

SETTING: Health Education England North-West, UK.

PARTICIPANTS: 47 surgical trainees (24 O&G and 23 GS) were sub-divided into four groups: 11 junior O&G, 13 senior O&G, 11 junior GS, and 12 senior GS trainees.

OBJECTIVES: Trainees were tested on four simulated laparoscopic tasks; laparoscopic camera navigation (LCN), hand eye co-ordination (HEC), bimanual co-ordination (BMC) and suturing with intracorporeal knot tying (suturing).

RESULTS: O&G trainees completed LCN (P <0.001), HEC (P <0.001) and BMC (P <0.001) significantly slower than GS trainees. Furthermore, O&G found fewer number of targets in LCN (P =0.001) and dropped a greater number of pins than the GS trainees in BMC (P =0.04). In all three tasks, there were significant differences between O&G and GS trainees but no difference between the juniors and senior groups within each specialty. Performance in suturing also varied by specialty; senior O&G trainees scored significantly lower than senior GS trainees; O&G 11.4 ± 4.4 vs GS 16.8 ± 2.1, P = 0.03. Whilst suturing scores improved with seniority among O&G trainees, there was no difference between the junior and senior GS trainees; senior O&G 11.4 ± 4.4 vs junior O&G 3.6 ± 2.1, P = 0.004.

DISCUSSION: GS trainees performed better than O&G trainees in core laparoscopic skills and the structure of obstetrics and gynaecology training may require modification.

Keywords: Laparoscopy; obstetrics; gynecology; surgical training

For peer review only

INTRODUCTION

The foundations of laparoscopic surgery were laid by gynaecologists and the first sterilisation procedure was performed laparoscopically in 1936.[1] Gynaecologists have led advancements in laparoscopy through innovation in laparoscopic instruments and educational tools such as the pelvic simulator trainer and Hasson's open technique for entry, which is widely used by general surgeons today. [1, 2]

Obstetrics and gynaecology (O&G) and general surgery (GS) trainees are required to demonstrate competency in different procedures, (3, 4) however, the core psychomotor skills required for laparoscopy are similar. Some of these skills include laparoscopic camera navigation (LCN), hand eye co-ordination (HEC) and bimanual co-ordination (BMC). Surgical trainees should be proficient in these skills early in their training to enable development of more complex and specific laparoscopic procedural techniques. [5, 6]

O&G training, lasting seven years, consists of basic (ST1-ST2), intermediate (ST3-ST5) and advanced training (ST6-ST7). The training covers both obstetrics and gynaecology although there is a significant focus on acquiring obstetric competencies throughout the training. [7] Exposure to laparoscopic surgery is gained only through gynaecological practice. Trainees who wish to pursue gynaecological training can select Advanced Training Skills Modules (ATSM) or subspecialisation relevant to gynaecological surgery in the advance part of the program. [8] In contrast, GS training is eight years long, including two years of core surgical training (CST1-2) and six years of higher surgical training (ST3-ST8), where the final two years focusses on subspecialty training (Figure 1). [4] GS trainees are required to be independent in laparoscopic appendicectomy by the end of CST2. [4] In contrast, O&G trainees are expected to perform 'minor operative laparoscopy' by the end of the fifth training year. [3] GS trainees, therefore, gain laparoscopic experience throughout their training programme whilst O&G trainees receive most of their laparoscopic surgery exposure in the advanced part of the program. [8, 9]

Opportunities for theatre experience appear to be lacking in both specialties. In 2021, the Royal College of Obstetricians and Gynaecologists (RCOG) evaluated the training of 1415 trainees and found that less than half of the ST5 and ST6 trainees reported adequate opportunities to develop the required surgical skills relevant to their stage of training.[10] Similarly, amongst 155 GS applicants certifying for completion of training, only two-thirds had reached the required number of cases. However, nearly three-quarters of these trainees had met the requirements for key procedures in their field. [11] The GS training program may be delivering better laparoscopic training than O&G. A comparative approach between these two related surgical specialties may enable us to characterise the challenges associated with the acquisition of core laparoscopic skills in both O&G and GS trainees.

Our study compared the proficiency in core laparoscopic psychomotor skills amongst junior (ST3-ST5 in both specialties) and senior trainees (ST6-ST7 in O&G ST6-ST8 in GS). We hypothesised that there is no difference in the performance of core laparoscopic skills between O&G and GS trainees at all training stages.

For peer review only

METHOD

Participants

47 trainees (24 O&G and 23 GS) from Health Education England North-West (HEENW) were invited to participate in this prospective observational study between September 2021 and April 2022. Trainees were allocated a study number, which was recognisable only to the two study investigators involved in the recruitment of trainees. To explore the effect of surgical experience, the trainees were sub-divided by their training grades into four groups: junior O&G, senior O&G, junior GS and senior GS.

The 'junior' group consisted of trainees between ST3 and ST5, and the 'senior' group included trainees in the final two years of O&G and GS training programs. For the senior O&G group, we selected trainees undertaking one of the advanced modules in 'advanced laparoscopy for the excision of benign disease', 'benign abdominal surgery - open and laparoscopic' and 'gynae-oncology'. This was to enable the selection of trainees in receipt of regular gynaecology theatre sessions and, therefore, comparable with GS seniors. Senior GS trainees with a specialist interest in breast surgery were excluded due to limited laparoscopic work within this subspecialty.

All participants provided written informed consent prior to participation. They completed a questionnaire collecting data on demographic details and factors relating to laparoscopic proficiency, such as the use of video games and laparoscopic simulators, attendance at courses involving laparoscopic surgery, training stage at first exposure to laparoscopic work, and the typical frequency of attendance in theatre.

The study was approved by the O&G and GS heads of schools from HEENW. Ethical approval was granted by the Faculty of Health and Medicine Research Ethics Committee at Lancaster University (FHMREC20033) and testing was conducted in accordance with the Declaration of Helsinki. Following ethical approval, the study was registered at clinicaltrials.gov (NCT05116332).

Procedures

All trainees were assessed by two faculty members/assessors in individual rooms to minimise external distractions. Assessors were not involved in the training of any study participants and trainees were able to discretely request a different assessor (s) if they knew the pre-assigned member or felt uncomfortable with them, without giving a reason. Trainee's specialty and training stage was concealed from the assessors to ensure anonymity of trainees and blinding of the assessors. Laparoscopic proficiency was measured by observing four standardised, simulated tasks using validated assessment tools. [5, 12-14] All trainees received the same written and video instructions explaining the task before beginning any assessments.[15] The first three tasks assessed core laparoscopic psychomotor skills using the Laparoscopic Skills Training and Testing (LASTT) model. [13] The fourth task evaluated laparoscopic suturing and was assessed using the suturing and knot tying training and testing (SUTT-1) method by the European Academy of Gynaecological Surgery. [16] Trainees performed each task three times, except for the suturing task, which was completed once. The rationale behind restricting repetition to three iterations was to familiarise trainees to the task so that their optimal performance could be elicited without inducing a significant rehearsal effect. [17]

The same equipment was used throughout the testing period for all trainees. All assessors received standardised training modified from the "Training the Trainers" of the Gynaecological Endoscopic Surgical

For peer review only

Education and Assessment (GESEA) program. This consisted of an overview of all study tasks, instruments, scoring systems, and specifics details relating to set up and delivery of all the study tasks. Tasks were performed in order of increasing technical difficulty as described below.

Tasks

Task 1 - Laparoscopic Camera Navigation (LCN)

This task assessed the trainees' ability to navigate a 30° laparoscope to find 14 targets within the LASTT model. [12-14] The maximum time allowed was 300 seconds per iteration. A validation study on the LASTT model showed that the median time for task completion was 188 seconds for novices and ranged between 142 and 292 seconds. [12] As O&G trainees do not routinely use a 30° laparoscope, we used the upper limit of the range as the allocated time.

On the scoring sheet, the time taken to identify all 14 targets, or the last target identified within 300 seconds, was recorded. The task was considered successful when all 14 targets were identified in every iteration within the allocated timeframe. The trainees' best time (of the three iterations) was used to assess the speed of task completion. To assess the trainees' ability to integrate speed with navigation skills, the ratio of the total number of targets found to the total time taken to complete the task was calculated.

Task 2 – Hand eye co-ordination (HEC)

This task required the trainee to transfer six coloured cylinders to their respective coloured pins using a forceps in their dominant hand and navigating a 0° laparoscope with their non-dominant hand. [12-14] Time permitted for this task was 180 seconds per iteration. [12, 13]

Completion was determined when six cylinders were placed on their pins within the allocated time. The trainees' best time was used to calculate the speed of task completion. We recorded the total number of times a cylinder was dropped during each iteration. A sum of the three iterations gave a total number of drops. This was used as an indicator of precision of movement.

Task 3 – Bimanual Co-ordination (BMC)

This task assessed the trainees' ability to transfer six coloured pushpins between forceps in their dominant and non-dominant hands and place them in their coloured slots on the LASTT model. [12-14] The assessor navigated the camera for the trainees based on their instructions. A maximum of 180 seconds was allowed per iteration and outcome measures were the same as for HEC.

Task 4 – Laparoscopic suturing and intracorporeal knot placement (suturing)

A foam pad was used to assess suturing and knot placement using the SUTT-1 method. [16] All trainees were shown a 60-second video demonstration of laparoscopic suturing and intracorporeal knot tying to ensure that the instructions were standardised, and expectations were clearly understood. [18] Trainees were asked to place four interrupted sutures and perform four intracorporeal knots comprising of three throws. A maximum of 15 minutes was permitted for this task. The quality of suturing and knot-tying was assessed by two experienced consultants (one O&G and one GS consultant; both with over 10 years of experience in laparoscopic suturing) after completion of the task using a validated SUTT scoring system. [16] The assessors were blinded to the trainee and each other's score. All elements of the total suturing score, such as extent of trauma, were scored after thorough inspection of the foam pads. The suturing task was deemed complete if four horizontal sutures and four secure knots were secured within 15 minutes. The median number of sutures and knots inserted (out of four) and the total suturing scores were analysed.

A summary of the surgical tasks and their assessment are provided in Table 1.

For peer review only - http://bmjopen.bmj.com/site/about/guidelines.xhtml

Table 1. Summary of laparoscopic surgical tasks and methods

Task	Iterations	Time allocated	Data recorded	Outcome
**1 – Laparoscopic camera navigation (LCN)**	3	300 seconds	Time taken to find 14 targets<br>If exceeding 300 seconds, the last target found	Best time*<br>No of targets found
**2 – Hand eye co-ordination (HEC)**	3	180 seconds	Time taken<br>No. of objects placed<br>No. of drops	Best time*<br>Overall no. of drops**
**3 – Bimanual co-ordination (BMC)**	3	180 seconds	Time taken<br>No. of objects placed<br>No. of drops	Best time*<br>Overall no. of drops**
**4 – Suturing and intracorporeal knot placement (suturing)**	1	15 minutes	Time taken<br>Quality of sutures and knots	Median no. of sutures and knots<br>Total suturing scores

*shortest completion time out of three iterations.
**sum of dropped objects across the three iterations.

Statistical analysis

The Chi squared test (χ^2) was used to analyse demographic, training related variables between specialties (Table 2) and successful completion of all tasks. All continuous variables are reported as mean, standard deviation and 95% confidence intervals.

Normality was checked for tasks 1-3, including the LCN time and efficiency ratio, HEC time and precision score, and BMC time and precision score. As normality was only confirmed for BMC time, a robust ANOVA [19, 20] was used to compare the junior and senior trainee groups within the two specialties. The Holm-Bonferroni post hoc test was carried out to locate the difference and adjust for multiple comparisons when a significant result was observed. Where trainee's surgical experience did not have a significant effect, robust independent t-tests were used to compare differences between O&G and GS. Effect sizes (ξ) were calculated for all significant comparisons and 0.1 was considered small, 0.3 moderate and 0.5 large.[21] BMC time was analysed using ANOVA to compare junior and senior trainee groups within the two specialties and independent t-tests to assess differences between specialties. Holm-Bonferroni post hoc test was carried out to locate the difference and adjust for multiple comparisons when a significant result was observed.

In the suturing task, the number of sutures and knots were compared between the four groups using the Kruskal Wallis test, with Holm-Bonferroni correction for multiple pairwise comparisons. This data is reported as median and interquartile range. Hedges *g* was calculated for all significant comparisons with 0.2, 0.5 and 0.8 considered as small, moderate and large, respectively. [22] Agreement of total suturing scores between assessors was examined with Cronbach's alpha. [23] According to Bland and Altman, α =0.95 is desirable for clinical applications.[24] Total suturing scores were analysed using robust statistics as above. Statistical analysis was conducted in Jamovi Version 2.3.18.0 (The Jamovi project,

For peer review only

https://www.jamovi.org) while collation and creation of figures was completed in Graphpad Prism v9 (GraphPad Software, San Diego, Calif., USA). Statistical significance was set at P≤0.05 and the corrected values are presented.

RESULTS

Participant Characteristics

Two trainees were excluded from the analysis as they did not meet the inclusion criteria (one senior O&G trainee) and had incomplete data (one senior GS trainee). 23 O&G trainees (mean ± SD, age 34 ± 4 years) and 22 GS trainees (34 ± 5 years) were selected for data analysis. The OG group consisted of 11 junior and 12 senior trainees and GS group consisted of 11 junior and 11 senior trainees. Both groups were not significantly different except their gender. Most O&G trainees were female in contrast to GS, where the majority were male.

Factors relating to proficiency in laparoscopic skills

Pre-testing baseline questionnaires showed that a significantly larger number of O&G trainees used a simulator than GS trainees; O&G 16 (70%) vs. GS 7 (32%), P =0.01. However, the number of trainees using the simulator frequently, such as once a month, was similar between the two specialties: O&G 3 (13%) vs. GS 2 (9%), P=0.32. O&G trainees reported attending significantly fewer elective and emergency laparoscopic theatre sessions; O&G 64 (37%) and 23 (19%) vs. GS 110 (63%) and 100 (81%), P <0.001 for both comparisons. However, analysis by training grade showed that senior O&G and senior GS trainees attended a similar number of elective sessions; O&G 51 (80%) vs. GS 56 (51%), P=0.30. Furthermore, junior O&G trainees were assigned to an assistant's role significantly more frequently than junior GS trainees; O&G 7 (64%) vs. GS 2 (18%), P =0.05 **(Table 2).**

Table 2. Laparoscopic training experience amongst O&G and GS trainees. Data is presented as frequencies (%). P values in bold indicate significant findings.

	O&G (n=23)	GS (n=22)	P
**Females**	15 (65%)	5 (13%)	**0.004**
**Males**	8 (35%)	17 (77%)	
**Juniors**	11 (48%)	11 (50%)	0.88
**Seniors**	12 (52%)	11 (50%)	
**Right handedness**	21 (91%)	19 (86%)	0.59
**Left/**ambidextrous	2 (9%)	3 (14%)	
**Played video games**	11 (48%)	8 (36%)	0.43
**Used pelvic simulator**	16 (70%)	7 (32%)	**0.01**
**Weekly**	2 (9%)	0 (0%)	
**Monthly**	3 (13%)	2 (9%)	0.32
**Less frequent**	18 (78%)	20 (91%)	
**Attended laparoscopic courses**	18 (78%)	20 (91%)	0.24
**Start of laparoscopic training:**			
**Core training**	14 (61%)	14 (67%)†	0.69
**Registrar training**	9 (39%)	7 (33%)	0.69
**Elective theatre sessions**	64 (37%)	110 (63%)	**<0.001**

**Junior**	13 (20%)	54 (49%)	**<0.001**
**Senior**	51 (80%)	56 (51%)	0.30
**Emergency theatre sessions/month**	23 (19%)	100 (81%)	**<0.001**
**Junior**	10 (43%)‡	46 (46%)	**0.003**
**Senior**	13 (57%)	54 (54%)	**<0.001**
**Type of exposure**			
**Juniors as Operator**	4 (36%)	9 (82%)	**0.03**
**Juniors as Assistant**	7 (64%)	2 (18%)	**0.03**
**Seniors as Operator**	10 (83%)	10 (91%)	0.59
**Seniors as Assistant**	2 (17%)	1 (9%)	0.59

† One junior GS trainee did not answer. ‡One junior O&G trainee did not answer.

Successful completion of tasks

Overall, O&G and GS trainees had 69 and 66 attempts at each of the three core tasks, respectively. A smaller number of attempts were successfully completed by O&G trainees in comparison to GS trainees on all three tasks (LCN task: O&G 50 (72%) vs. GS 64 (97%), $P < 0.001$; HEC task: O&G 54 (78%) vs. GS 64 (97%), $P = 0.001$; BMC task: O&G 47 (68%) vs. GS 62 (94%), $P < 0.001$).

Task Completion times (Speed)

There was a significant effect of specialty on completion times for LCN; $F(3,33)= 6.26$, $P=0.005$, HEC; $F(3,33)=7.34$, $P=0.002$, BMC; $F(3,41)=11.6$, $P<0.001$. Post hoc analyses showed significant differences between junior O&G and junior GS trainees only and no significant difference was found within the specialty groups, (i.e., between junior and senior trainees in either specialty). Between groups comparison showed that O&G specialty trainees were 73 seconds slower at completing LCN; O&G 166 ± 56, (139 to 193) seconds vs. GS 93 ± 21 (83 to 103) seconds, $t(21)= 4.17$, $P<0.001$, Effect size (ξ) = 0.76. O&G trainees were also significantly slower at HEC; O&G 105 ± 30 (90 to 119) seconds vs GS 67 ± 13 (60 to 73) seconds, $t(25.6)=3.98$, $P<0.001$, $\xi= 0.66$ and BMC task; O&G 139 ± 32 (125 to 153) seconds vs GS 100 ± 20 (92 to 109) seconds, $t(43)= 4.74$, $P<0.001$, $\xi= 1.41$. **(Figure 2a-c).**

Precision of movements (Accuracy)

Specialty had a significant effect on the precision of movements in LCN; $F(3,33)=8.23$, $P=0.001$, and BMC; $F(3,33)=3.37$, $P=0.04$. However, no significant difference was found in the precision of movements in HEC; $F(3,33)= 0.96$, $P=0.43$. Post hoc analysis showed that greater trainee experience did not significantly affect precision outcomes on these tasks. Therefore, the data was analysed by overall specialty. Overall, in LCN, O&G trainees found fewer targets, in the given time, than GS trainees; O&G 0.09 ± 0.04, (0.07 to 0.10) vs. GS 0.16 ± 0.03, (0.14 to 0.17), $t(31.6)= 5.27$, $P<0.001$, $\xi= 0.82$. In BMC, O&G trainees dropped a significantly greater number of pins than GS trainees; O&G 5.4 ± 2.3 (4.3 to 6.6) vs. GS 2.9 ± 1.7 (2.1 to 3.8), $t(32.8)=3.03$ $P =0.005$, $\xi=0.53$. O&G and GS trainees both dropped similar number of cylinders during HEC task; O&G 3.5 ± 2.7 (2.2 to 4.8) vs. GS 2.3 ± 1.6 (1.5 to 3.1), $t(32.2)=1.23$, $P=0.22$, $\xi=$ 0.27. **(Figure 2 d-f).**

For peer review only

Suturing

The inter-rater agreement of the assessors on the suturing task was very high (Cronbach's alpha 0.98 for O&G and 0.97 for GS). One O&G trainee (4.3%) and eight GS trainees (36%) completed this task in time; P =0.007.

Number of inserted sutures and knots

Overall, O&G junior trainees were able to place fewer sutures and tie fewer intracorporeal knots than junior GS trainees (sutures: O&G 1 (1-1) vs. GS 4 (3-4), P = 0.005, Hedges g =0.98; knots: O&G 0 (0-1) vs. GS 2 (2-4), P = 0.005, g= 0.95). Senior O&G trainees tied significantly fewer knots than senior GS trainees (O&G 2.5 (1-3) vs. GS 4 (3-4), P = 0.03, g =0.51). However, senior trainees in O&G and GS groups placed similar number of sutures (O&G 3 (2-3) vs. GS 4 (3-4), P = 0.07, g =0.4).

Total suturing scores

O&G trainees had a significantly lower total suturing score than the GS trainees; F(3,33)=36.3, P <0.001). Post hoc analysis showed that junior O&G trainee's total suturing score was significantly lower than junior GS trainees; O&G 3.6 ± 2.1, (1.97 to 5.14) vs GS 14.9 ± 4.4 (11.5 to 18.3), P<0.001 and senior O&G trainees also scored lower than senior GS trainees; O&G 11.4 ± 4.4 (8.2 to 14.6) vs GS 16.8 ± 2.1 (15.2 to 18.4), P= 0.03. Senior O&G trainees had a significantly higher total suturing score than junior O&G trainees; Senior O&G 11.4 ± 4.4 (8.23 to 14.6) vs. Junior O&G 3.6 ± 2.1 (1.97 to 5.14), P = 0.004. Senior GS trainees, however, scored like their junior colleagues; Senior GS 16.8 ± 2.1 (15.2 to 18.4) vs. Junior GS 14.9 ± 4.4 (11.5 to 18.3), P = 0.35 (**Figure 3).**

DISCUSSION

Principal findings

The acquisition of core laparoscopic skills depends on multiple factors including exposure to large volumes of laparoscopic procedures, [25] deliberate practice, [26] and structured simulation programs. [27] It is unknown whether the differing design of O&G and GS training leads to differential attainment of laparoscopic skills. Our study found that GS trainees performed better than O&G trainees in all tasks that measured core laparoscopic psychomotor skills. This may, in part, be due to the discrepancy in the volume of laparoscopic practice between the two specialties. Our baseline questionnaire showed that the average GS trainee attended the operating theatre almost three times as often as the average O&G trainee and were more likely to perform as the main operator in contrast to O&G trainees.

Our study found that increased training experience had an impact on suturing and knot tying but not on the other three core laparoscopic tasks. This may be due to the simplicity of these core tasks. Surgical skills such as navigating a camera and retracting surgical tissue are usually learnt early in the training and probably reach a plateau phase rather quickly. As trainees progress, they are typically required to synchronise movements of both hands and eyes. It has , therefore, been shown that participants rapidly reached their optimal performance on simple tasks such as HEC and that despite further training no significant improvements were seen in performance. [5] Suturing, however, is regarded as a complex task and has been shown to improve with greater surgical experience. [28]

Meaning of the study: possible explanations and implications for clinicians and policymakers

For peer review only

O&G trainees need to acquire experience and craft competencies in obstetrics at an early stage in the training program. The hyperacute nature of obstetric emergencies and the 24/7 nature of the specialty demands early proficiency in obstetrics to enable effective participation in the on-call rotas. Most of the emergency work in O&G relates to obstetrics and exposure to out-of-hours laparoscopic procedures are therefore limited. [29] Our study confirmed the limited volume of out-of-hours laparoscopic exposure amongst O&G trainees.

Overall, O&G trainees attended fewer laparoscopic theatre sessions and were less likely to be given the main operator's role than their GS counterparts. However, this difference was largely between the junior trainees only. Our baseline questionnaire showed that senior O&G trainees, in fact, attended a similar number of elective theatre sessions as the senior GS trainees and they reported acting as the 'main operator' almost as frequently as the senior GS trainees. It appears that in O&G, theatre exposure and operative opportunities are concentrated in the latter part of the training. Although senior O&G trainees performed significantly better than their junior O&G colleagues, their suturing performance still lagged behind the senior GS trainees. This suggests that concentrated late exposure to laparoscopic surgery may not be as effective as consistent exposure throughout training. Psychological techniques have consistently shown that distributed practice is superior to concentrated practice and leads to the enhanced acquisition, consolidation, and retention of surgical skills. [30, 31]

The Royal College of Obstetricians and Gynaecologists (RCOG) expects all senior (advanced) trainees to be independent in laparoscopic salpingectomy (a procedure used for removing tubal ectopic pregnancy). [3] However, senior trainees' competency in salpingectomy has been shown to vary between 32% and 89%. [32, 33] More timely introduction of salpingectomy into the O&G curriculum would lead to earlier and more sustained exposure and allow trainees adequate opportunities to reach proficiency not only in this key procedure but also facilitate acquisition of more complex skills such as laparoscopic suturing. [5]

A greater number of O&G trainees reported using a pelvic simulator, however, only a minority reported using it frequently. Surgical skills learnt on simulators can apparently be transferred to real patient surgery, but these benefits are mostly observed with repetitive practice and as part of a structured simulation program. [34-36] The latter is promoted as a solution for bridging the gap between required operative skills and reduced training opportunities. [37, 38] In this context the American College of Obstetricians & Gynaecologist have included the Fundamental of Laparoscopic Surgery, a structured simulation program, as part of board certification for practice in O&G. [39]

The competing demands of delivering an O&G training program committed to delivering generalist as well as specialist training within seven years is unrealistic and results in a failure to balance the training aspirations of its trainees with the demands of providing a consultant led service. [40] With the inexorable move to minimally invasive surgery, it is of key importance that O&G curricula and training structure adapts to allow the right trainees to be selected and trained more intensely by appropriately skilled surgeons.

Strengths and limitations of the study

To our knowledge, this is the first prospective study to examine trainees' laparoscopic skills in two surgical specialties who work in an anatomically similar environment. The training tools in this study were based on widely used and validated assessments, [12, 13] and our inter-observer reliability for the suturing assessments was very high. The two assessors were not involved with the individual participants' training, and they were blinded to the trainee's specialty, experience and to each other's scores. This study was

For peer review only

localised to the North-West region of the UK and testing it on a national level would provide more precision around the estimates of skill and enhance external validity.

The sample size may appear small for an observational study. Nonetheless, there are no previous studies available examining a similar aspect, and due to the difficulties in estimating the minimum difference considered important in this context, a priori sample size estimation was not possible. Consequently, along with the mean and SD values, we also included CIs and effect sizes to enable future meta-analysis as well as inform readers of the precision and magnitude of the results.

Unanswered questions and future research

The validity of evaluating core psychomotor skills in laparoscopic surgery needs to be assessed against actual performance in the operating theatre. Our work showed that trainees with limited experience found suturing (an actual surgical procedure) challenging but not the core psychomotor tasks. This implies that it is not just the mastery of core skills, but the cognitive and motor processes involved in applying these skills which may influence performance on actual surgical procedures. Therefore, future studies could look at cognitive and musculoskeletal stress amongst the two specialities and the seniority of its trainees.

WHAT IS ALREADY KNOWN ON THIS TOPIC

- Over the years, operative training opportunities have been declining in both O&G and GS.
- Both specialties expect similar proficiency in core laparoscopic psychomotor skills from their trainees, but surgical training structures differ.
- A study comparing the acquisition of these core surgical skills in O&G and GS trainees has never been undertaken.

WHAT THIS STUDY ADDS

- Our study suggests that GS trainees may have greater proficiency in core laparoscopic skills than O&G trainees.
- Comparative data should be used to inform both the research and training agenda to help identify the key components of effective surgical training pathways with a view to improving postgraduate surgical training and ultimately patient care.

For peer review only

Acknowledgements: We would like to thank Karl Storz, Medtronic, BSGE and BRIDGES society for their financial support with the study. We would also like to thank Mr Brice Rodriguez, Mr Kenn Ma, Dr Alison Sambrook, Mr Kenn Lim, Mr Mohamed Elsherbiny, Mr Mohamed Abdelrahman, Ms Christa Hammill, Ms Aleyamma Abraham and Dr Nicholas Evans for their help with study protocol and conducting this study.

Funding: This study was funded by grants from the British Society of Gynaecological Endoscopy, Karl Storz and Medtronic.

Conflict of interest: None declared.

Author statement: DAS and ZK conceived and developed the research idea. ZK, DS, DAS, CG, TJC and EH designed and implemented the study protocol. All authors conducted the study. TB, CG and ZK analysed

the data. ZK, DS, AS, TJC, TB, CG, and DAS prepared the manuscript. All authors reviewed and approved the final manuscript.

Data statement: Data are available from the corresponding author (CG) upon reasonable request.

REFERENCES

1. Harrell AG, Heniford BT. Minimally invasive abdominal surgery: lux et veritas past, present, and future. The American Journal of Surgery. 2005;190(2):239-43.
2. Hasson H, Rotman C, Rana N, Kumari NA. Open Laparoscopy: 29- Years Experience. Obstet Gynecol. 2000;96:763-6.
3. Royal College of Obstetricians and Gynaecologists. Royal College of Obstetricians and Gynaecologists, Core Curriculum for Obstetrics and Gynaecology: RCOG; 2019 [updated June 2020. Available from: https://www.gmc-uk.org/-/media/documents/core-og-curriculum-2019-final-gmc-approved-20210504_pdf-79992893.pdf.
4. Intercollegiate surgical curriculum program (ISCP). Intercollegiate surgical curriculum program, Core Surgical Training Curriculum: ISCP; 2021 [updated Aug 2021. Available from: https://www.iscp.ac.uk/media/1326/core-surgical-training-curriculum-2021-minor-changes-for-august-2022.pdf.
5. Molinas CR, Campo R. Defining a structured training program for acquiring basic and advanced laparoscopic psychomotor skills in a simulator. Gynecological Surgery. 2010;7(4):427-35.
6. Molinas CR, Campo R. Retention of laparoscopic psychomotor skills after a structured training program depends on the quality of the training and on the complexity of the task. Gynecol Surg. 2016;13(4):395-402.
7. Royal College of Obstetricians and Gynaecologists. Royal College of Obstetricians and Gynaecologists, Training matrix London: RCOG; 2022 [Available from: https://www.rcog.org.uk/careers-and-training/starting-your-og-career/specialty-training/assessment-and-progression-through-training/training-matrix/.
8. Royal College of Obstetricians and Gynaecologists. Royal College of Obstetricians and Gynaecologists, Advanced Training in Obstetrics & Gynaecology: RCOG; 2019 [updated Aug 2019. Available from: https://www.rcog.org.uk/media/xigldqq1/advanced-training-definitive-document-2019.pdf.
9. McMurray R, Lawrence T, Afors K. Minimal access in gynaecological surgery: training the minimally invasive gynaecological surgeon. Obstetrics, Gynaecology & Reproductive Medicine. 2022;32(7):135-40.
10. Royal College of Obstetricians and Gynaecologists. Royal College of Obstetricians and Gynaecologists, Training Evaluation Feedback on Gynaecology training London: RCOG; 2021 [updated c2021. Available from: https://www.rcog.org.uk/media/vrkcsb4b/tef-2021-report-gynaecology-training.pdf.
11. Thomas C, Griffiths G, Abdelrahman T, Santos C, Lewis W. Does UK surgical training provide enough experience to meet today's training requirements? Bmj [Internet]. 2015 [cited 2022 October]; 350(h2503). Available from: https://www.bmj.com/content/350/bmj.h2503.
12. Molinas CR, De Win G, Ritter O, Keckstein J, Miserez M, Campo R. Feasibility and construct validity of a novel laparoscopic skills testing and training model. Gynecological surgery. 2008;5(4):281-90. eng.
13. Campo R, Reising C, Van Belle Y, Nassif J, O'Donovan P, Molinas CR. A valid model for testing and training laparoscopic psychomotor skills. Gynecological surgery. 2010;7(2):133-41. eng.
14. Campo R, Wattiez A, Leon De Wilde R, Molinas Sanabria CR. Training in laparoscopic surgery: From the lab to the or. Slovenian Journal of Public Health. 2012;51(4):285-98.
15. European Academy of Gynaecological Surgery. European Academy of Gynaecological Surgery, Laparoscopic Skills Training and Testing Method Europe: MiS Academy Europe; 2022 [Available from: https://europeanacademy.org/training-tools/lastt/.
16. Sleiman Z, Tanos V, Van Belle Y, Carvalho JL, Campo R. European Academy laparoscopic "Suturing Training and Testing'' significantly improves surgeons' performance. Facts Views Vis Obgyn. 2015;7(3):153-60. eng.
17. Feldman LS, Cao J, Andalib A, Fraser S, Fried GM. A method to characterize the learning curve for performance of a fundamental laparoscopic simulator task: defining "learning plateau" and "learning rate". Surgery. 2009;146(2):381-6.
18. LapPass Academy [online video]. Journal of surgical simulation. Laparoscopic Intracorporeal Suturing and Knot Tying, task 4. 1 video: 130 sec, HD, colour. UK: Journal of surgical simulation; 2020.

19. Field AP, Wilcox RR. Robust statistical methods: A primer for clinical psychology and experimental psychopathology researchers. Behav Res Ther. 2017;98:19-38.
20. Daszykowski M, Kaczmarek K, Vander Heyden Y, Walczak B. Robust statistics in data analysis — A review. Chemometrics and Intelligent Laboratory Systems. 2007;85(2):203-19.
21. Wilcox RR, Tian TS. Measuring effect size: a robust heteroscedastic approach for two or more groups. Journal of Applied Statistics. 2011;38(7):1359-68.
22. Brydges CR. Effect Size Guidelines, Sample Size Calculations, and Statistical Power in Gerontology. Innov Aging. 2019;3(4):1-8.
23. Tavakol M, Dennick R. Making sense of Cronbach's alpha. Int J Med Educ. 2011 Jun 27;2:53-5.
24. Bland JM, Altman DG. Statistics notes: Cronbach's alpha. BMJ [Internet]. 1997; 314(572).
25. Sadideen H, Alvand A, Saadeddin M, Kneebone R. Surgical experts: born or made? Int J Surg. 2013;11(9):773-8.
26. Reznick RK, MacRae H. Teaching Surgical Skills — Changes in the Wind. N Engl J Med. 2006;355(25):2664-9. eng.
27. Dawe SR, Pena GN, Windsor JA, Broeders JAJL, Cregan PC, Hewett PJ, et al. Systematic review of skills transfer after surgical simulation-based training. British Journal Of Surgery. 2014;101(9):pp1063-76. eng.
28. Train AT, Hu J, Narvaez JRF, Towle-Miller LM, Wilding GE, Cavuoto L, et al. Teaching surgery novices and trainees advanced laparoscopic suturing: a trial and tribulations. Surgical endoscopy. 2020;35(10):5816-26. eng.
29. Odejinmi F, Rizzuto I, Ballard KD. Potential barriers to the laparoscopic management of ectopic pregnancies: a regional UK study. Acta Obstet Gynecol Scand. 2010;89(10):1350-3. eng.
30. Cecilio-Fernandes D, Cnossen F, Jaarsma D, Tio RA. Avoiding Surgical Skill Decay: A Systematic Review on the Spacing of Training Sessions. J Surg Educ. 2018;75(2):471-80.
31. Gallagher AG, Jordan-Black JA, O'Sullivan GC. Prospective, randomized assessment of the acquisition, maintenance, and loss of laparoscopic skills. Ann Surg. 2012;256(2):387-93.
32. Moss EL, Bredaki FE, Jones PW, Hollingworth J, Luesley DM, Chan KK. Is gynaecological surgical training a cause for concern? A questionnaire survey of trainees and trainers. BMC Med Educ. 2011;11(1):32-. eng.
33. Christopoulos G, Kelly T, Lavery S, Trew G. Surgical skills of specialty trainees in emergency gynaecological laparoscopic procedures: a national UK survey. J Obstet Gynaecol. 2014;34(5):435-8.
34. Issenberg SB, McGaghie WC, Petrusa ER, Lee Gordon D, Scalese RJ. Features and uses of high-fidelity medical simulations that lead to effective learning: a BEME systematic review. Med Teach. 2005;27(1):10-28.
35. Gala R, Orejuela F, Gerten K, Lockrow E, Kilpatrick C, Chohan L, et al. Effect of validated skills simulation on operating room performance in obstetrics and gynecology residents: a randomized controlled trial. Obstet Gynecol. 2013;121(3):578-84.
36. Palter VN, Grantcharov TP. Development and validation of a comprehensive curriculum to teach an advanced minimally invasive procedure: a randomized controlled trial. Ann Surg. 2012;256(1):25-32.
37. Aggarwal R, Ward J, Balasundaram I, al. e. Proving the effectiveness of virtual reality simulation for training in laparoscopic surgery. Ann Surg. 2007;246(5):771-9.
38. Bryant-Smith A, Rymer J, Holland T, Brincat M. 'Perfect practice makes perfect': the role of laparoscopic simulation training in modern gynaecological training. The obstetrician & gynaecologist. 2020;22(1):69-74. eng.
39. American College of Obstetricians & Gynaecologists. American College of Obstetricians & Gynaecologists Washington: ACOG; 2022 [updated c2022. Available from: https://www.acog.org/education-and-events/creog/curriculum-resources/additional-curricular-resources/fundamentals-of-laparoscopic-surgery.
40. Royal College of Obstetricians and Gynaecologists. Royal College of Obstetricians and Gynaecologists, Becoming tomorrow's Specialist London: RCOG; 2014 [updated Sep 2014. Available from: https://www.rcog.org.uk/media/iglkfjri/becoming-tomorrows-specialist.pdf.

For peer review only

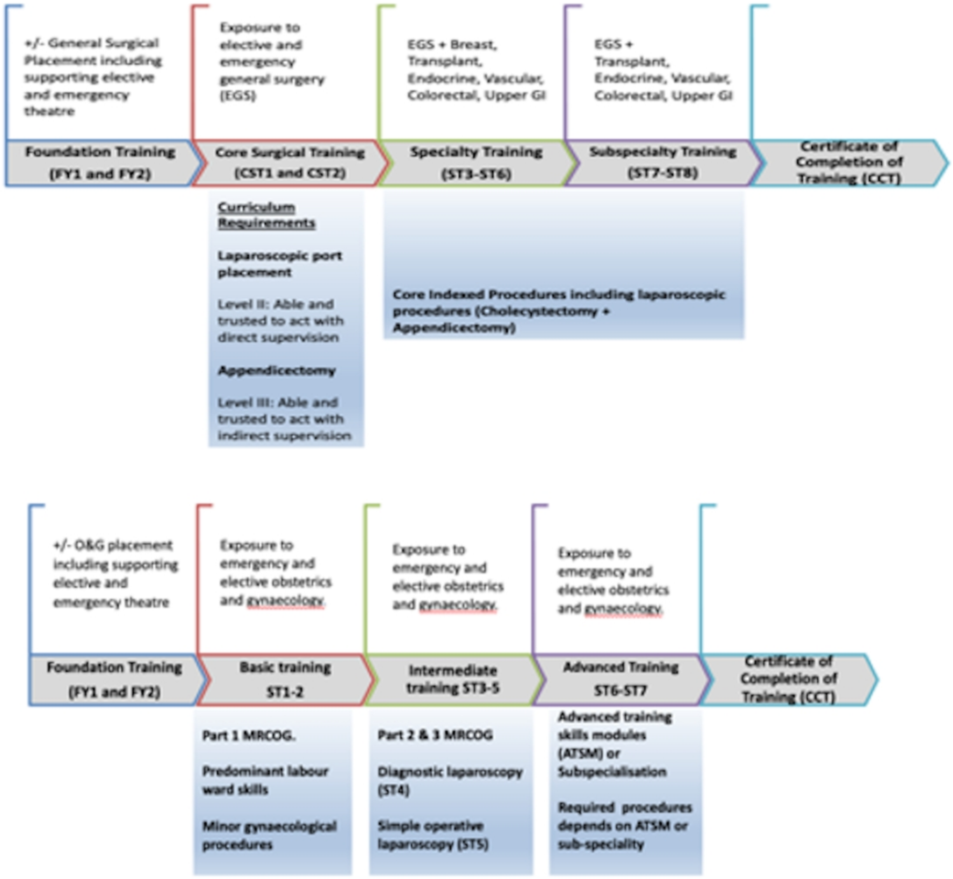

Figure 1: Outline of the training pathways in O&G (top) and GS (bottom). Adapted from the Royal College of Obstetricians & Gynaecologists (RCOG) (3) and intercollegiate surgical curriculum program (ISCP). (4)

478x443mm (118 x 118 DPI)

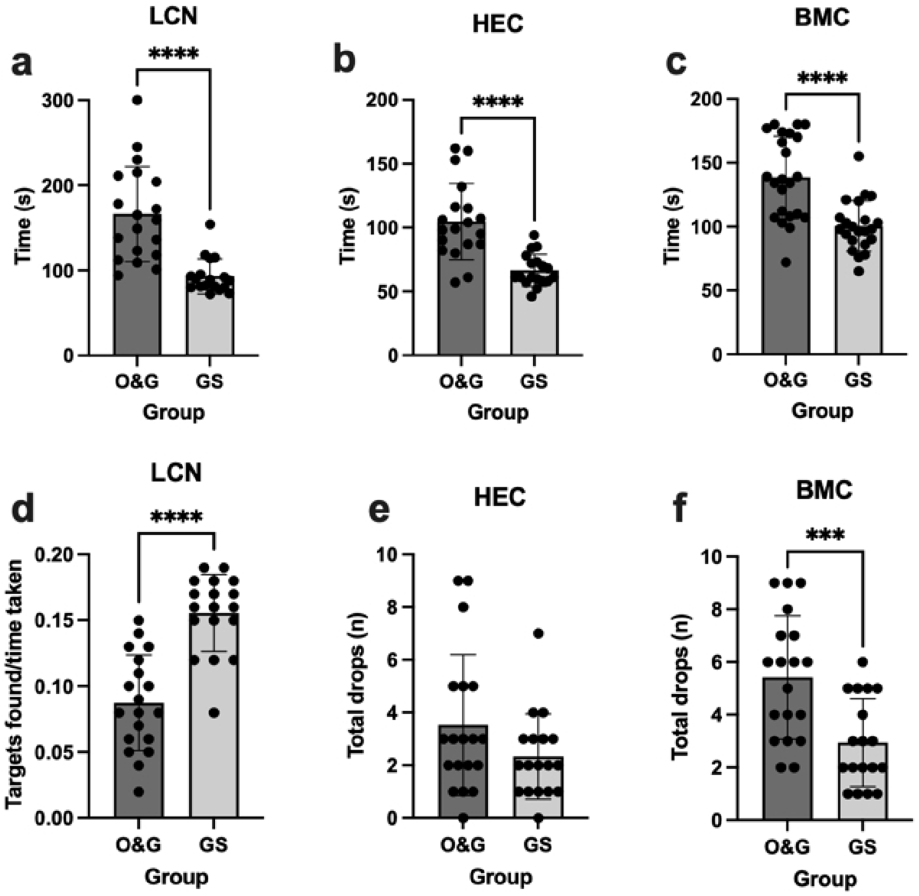

Figure 2. Time taken to complete laparoscopic tasks (a-c) and laparoscopic precision of movements by speciality (d-f). Data are presented as mean ± SD. Task completion time for LCN (a), HEC (b) and BMC (c). Trainees' ability to integrate camera navigation skills with speed (d), the number of drops in HEC (e) and the number of drops in BMC (f).

175x164mm (100 x 100 DPI)

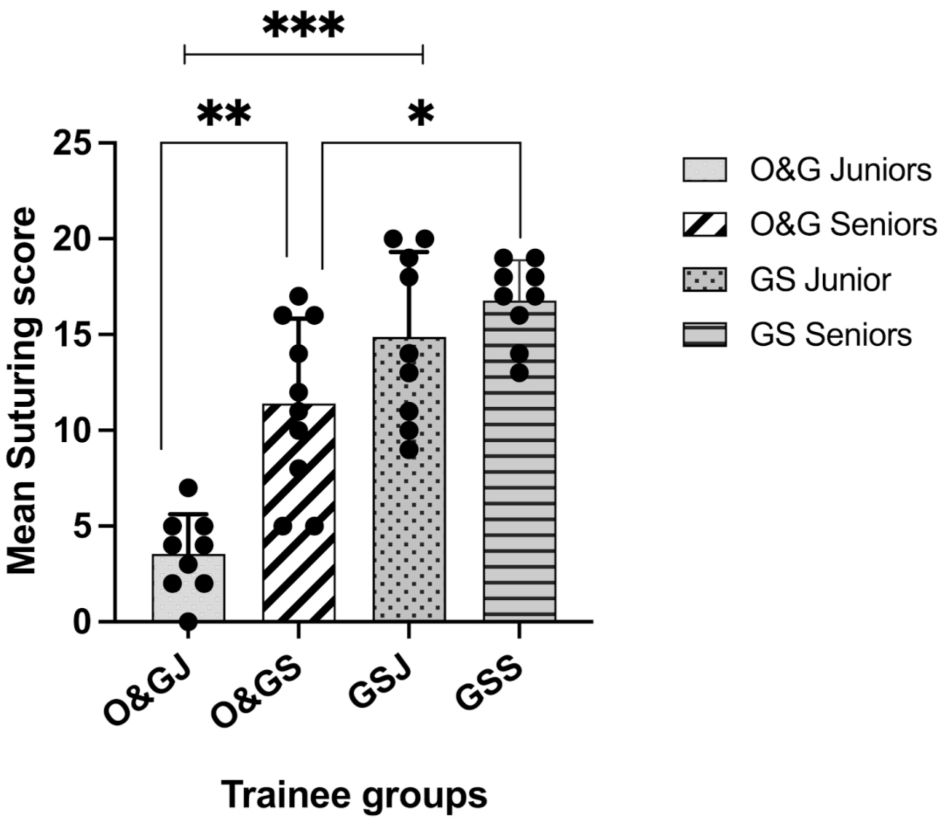

Figure 3. Total suturing scores by trainee’s experience within O&G and GS. Data are presented as mean ± SD.

128x108mm (300 x 300 DPI)

Reporting checklist for cross sectional study.

Based on the STROBE cross sectional guidelines.

Instructions to authors

Complete this checklist by entering the page numbers from your manuscript where readers will find each of the items listed below.

Your article may not currently address all the items on the checklist. Please modify your text to include the missing information. If you are certain that an item does not apply, please write "n/a" and provide a short explanation.

Upload your completed checklist as an extra file when you submit to a journal.

In your methods section, say that you used the STROBE cross sectionalreporting guidelines, and cite them as:

von Elm E, Altman DG, Egger M, Pocock SJ, Gotzsche PC, Vandenbroucke JP. The Strengthening the Reporting of Observational Studies in Epidemiology (STROBE) Statement: guidelines for reporting observational studies.

For peer review only

	Reporting Item	Page Number
**Title and abstract**		
Title	Indicate the study’s design with a commonly used term in the title or the abstract	1
Abstract	Provide in the abstract an informative and balanced summary of what was done and what was found	2
**Introduction**		
Background / rationale	Explain the scientific background and rationale for the investigation being reported	3
Objectives	State specific objectives, including any prespecified hypotheses	3
**Methods**		
Study design	Present key elements of study design early in the paper	4
Setting	Describe the setting, locations, and relevant dates, including periods of recruitment, exposure, follow-up, and data collection	4

Eligibility criteria	Give the eligibility criteria, and the sources and methods of selection of participants.	4
	Clearly define all outcomes, exposures, predictors, potential confounders, and effect modifiers. Give diagnostic criteria, if applicable	5-6
Data sources / measurement	For each variable of interest give sources of data and details of methods of assessment (measurement). Describe comparability of assessment methods if there is more than one group. Give information separately for for exposed and unexposed groups if applicable.	5-6
Bias	Describe any efforts to address potential sources of bias	5-6
Study size	Explain how the study size was arrived at	Sample size not possible to calculate as no previous study on this topic to base clinically significant effect size, and standard deviation measures on.
Quantitative variables	Explain how quantitative variables were handled in the analyses. If applicable, describe which groupings were chosen, and why	6
Statistical methods	Describe all statistical methods, including those used to control for confounding	7
Statistical methods	Describe any methods used to examine subgroups and interactions	7
Statistical methods	Explain how missing data were addressed	7
Statistical methods	If applicable, describe analytical methods taking account of sampling strategy	NA
Statistical methods	Describe any sensitivity analyses	NA

Results

For peer review only

Participants	Report numbers of individuals at each stage of study—eg numbers potentially eligible, examined for eligibility, confirmed eligible, included in the study, completing follow-up, and analysed. Give information separately for for exposed and unexposed groups if applicable.	7
Participants	Give reasons for non-participation at each stage	7
Participants	Consider use of a flow diagram	NA
Descriptive data	Give characteristics of study participants (eg demographic, clinical, social) and information on exposures and potential confounders. Give information separately for exposed and unexposed groups if applicable.	8
Descriptive data	Indicate number of participants with missing data for each variable of interest	7-8
Outcome data	Report numbers of outcome events or summary measures. Give information separately for exposed and unexposed groups if applicable.	8-10
Main results	Give unadjusted estimates and, if applicable, confounder-adjusted estimates and their precision (eg, 95% confidence interval). Make clear which confounders were adjusted for and why they were included	7-10
Main results	Report category boundaries when continuous variables were categorized	9-10
Main results	If relevant, consider translating estimates of relative risk into absolute risk for a meaningful time period	No risks/RR reported
Other analyses	Report other analyses done—e.g., analyses of subgroups and interactions, and sensitivity analyses	9-10
**Discussion**		
Key results	Summarise key results with reference to study objectives	11
Limitations	Discuss limitations of the study, taking into account sources of potential bias or imprecision. Discuss both direction and magnitude of any potential bias.	12

For peer review only

Interpretation	Give a cautious overall interpretation considering objectives, limitations, multiplicity of analyses, results from similar studies, and other relevant evidence.	11-12
Generalisability	Discuss the generalisability (external validity) of the study results	12
**Other Information**		
Funding	Give the source of funding and the role of the funders for the present study and, if applicable, for the original study on which the present article is based	13

None The STROBE checklist is distributed under the terms of the Creative Commons Attribution License CC-BY. This checklist can be completed online using https://www.goodreports.org/, a tool made by the EQUATOR Network in collaboration with Penelope.ai

For peer review only

BMJ Open

Performance in simulated core laparoscopic skills is superior in general surgery trainees than obstetrics and gynaecology trainees: a prospective observational study

Journal:	*BMJ Open*
Manuscript ID	bmjopen-2023-075113.R1
Article Type:	Original research
Date Submitted by the Author:	18-Aug-2023
Complete List of Authors:	Khan, Zaibun N; Royal Lancaster Infirmary<br>Shrestha, Donna; Lancaster University<br>Shugaba, Abdulwarith; Lancaster University<br>Lambert, Joel E; Lancaster University<br>Clark, T; Birmingham Women's NHS Foundation Trust, Gynaecology; University of Birmingham,<br>Haslett, Elizabeth; Blackpool Victoria Hospital<br>Afors, Karolina; Whittington Health NHS Trust<br>Bampouras, Theodoros M; School of Sport and Exercise Sciences<br>Gaffney, Christopher; Lancaster University<br>Subar, Daren; East Lancashire Hospitals NHS Trust
**Primary Subject Heading**:	Surgery
Secondary Subject Heading:	Obstetrics and gynaecology, Medical education and training
Keywords:	OBSTETRICS, GYNAECOLOGY, MEDICAL EDUCATION & TRAINING

SCHOLARONE™
Manuscripts

I, the Submitting Author has the right to grant and does grant on behalf of all authors of the Work (as defined in the below author licence), an exclusive licence and/or a non-exclusive licence for contributions from authors who are: i) UK Crown employees; ii) where BMJ has agreed a CC-BY licence shall apply, and/or iii) in accordance with the terms applicable for US Federal Government officers or employees acting as part of their official duties; on a worldwide, perpetual, irrevocable, royalty-free basis to BMJ Publishing Group Ltd ("BMJ") its licensees and where the relevant Journal is co-owned by BMJ to the co-owners of the Journal, to publish the Work in this journal and any other BMJ products and to exploit all rights, as set out in our licence.

The Submitting Author accepts and understands that any supply made under these terms is made by BMJ to the Submitting Author unless you are acting as an employee on behalf of your employer or a postgraduate student of an affiliated institution which is paying any applicable article publishing charge ("APC") for Open Access articles. Where the Submitting Author wishes to make the Work available on an Open Access basis (and intends to pay the relevant APC), the terms of reuse of such Open Access shall be governed by a Creative Commons licence – details of these licences and which Creative Commons licence will apply to this Work are set out in our licence referred to above.

Other than as permitted in any relevant BMJ Author's Self Archiving Policies, I confirm this Work has not been accepted for publication elsewhere, is not being considered for publication elsewhere and does not duplicate material already published. I confirm all authors consent to publication of this Work and authorise the granting of this licence.

For peer review only

Performance in simulated core laparoscopic skills is superior in general surgery trainees than obstetrics and gynaecology trainees: a prospective observational study

Zaibun N Khan[1] (0000-0002-9558-8964), Donna Shrestha [2] (0000-0001-8711-3515), Abdulwarith Shugaba [2], Joel E Lambert [2] (0000-0001-5663-6284), T Justin Clark [3] (0000-0002-5943-1062), Elizabeth Haslett [4] (0000-0003-1165-0415), Karolina Afors [5], Theodoros M Bampouras [6] (0000-0002-8991-4655), Christopher J Gaffney [2]* (0000-0001-7990-2792), Daren A Subar [7].

Affiliations:

1. Royal Lancaster Infirmary, Ashton Rd, Lancaster, LA1 4RP, UK.

2. Lancaster Medical School, Lancaster University, Health Innovation One, Sir John Fisher Drive, Lancaster, LA1 4AT, UK.

3. Birmingham Women's Hospital, Birmingham Women's & Children's NHS Foundation Trust, Metchley Park Road, Edgbaston, Birmingham, B15 2TG, UK.

4. Blackpool Victoria Hospital, Whinney Heys Road, Blackpool, FY3 8NR.

5. Whittington Health NHS Trust, Magdala Avenue, London N19 5NF, UK.

6. Liverpool John Moores University, School of Sport and Exercise Sciences, Tom Reilly Building, Byrom Street, Liverpool, L3 3AF, UK.

7. East Lancashire Hospitals NHS Trust, Haslingden Road, Blackburn, BB2 3HH, UK.

Correspondence to: Dr Christopher J Gaffney, Lancaster Medical School, Lancaster University, Health Innovation One, Sir John Fisher Drive, Lancaster, LA1 4AT, UK; c.gaffney@lancaster.ac.uk; +44 (0) 1524 593 602

Word count: 3984

For peer review only

ABSTRACT

BACKGROUND: Training programmes for obstetrics and gynaecology (O&G) and general surgery (GS) vary significantly, but both require proficiency in laparoscopic skills. We sought to determine performance in each specialty.

DESIGN: Prospective, Observational study.

SETTING: Health Education England North-West, UK.

PARTICIPANTS: 47 surgical trainees (24 O&G and 23 GS) were sub-divided into four groups: 11 junior O&G, 13 senior O&G, 11 junior GS, and 12 senior GS trainees.

OBJECTIVES: Trainees were tested on four simulated laparoscopic tasks; laparoscopic camera navigation (LCN), hand eye co-ordination (HEC), bimanual co-ordination (BMC) and suturing with intracorporeal knot tying (suturing).

RESULTS: O&G trainees completed LCN ($P < 0.001$), HEC ($P < 0.001$) and BMC ($P < 0.001$) significantly slower than GS trainees. Furthermore, O&G found fewer number of targets in LCN ($P = 0.001$) and dropped a greater number of pins than the GS trainees in BMC ($P = 0.04$). In all three tasks, there were significant differences between O&G and GS trainees but no difference between the juniors and senior groups within each specialty. Performance in suturing also varied by specialty; senior O&G trainees scored significantly lower than senior GS trainees; O&G 11.4 ± 4.4 vs GS 16.8 ± 2.1, $P = 0.03$. Whilst suturing scores improved with seniority among O&G trainees, there was no difference between the junior and senior GS trainees; senior O&G 11.4 ± 4.4 vs junior O&G 3.6 ± 2.1, $P = 0.004$.

DISCUSSION: GS trainees performed better than O&G trainees in core laparoscopic skills and the structure of obstetrics and gynaecology training may require modification.

Keywords: Laparoscopy; obstetrics; gynaecology; surgical training

For peer review only

Strengths and Limitations

- This is the first study to compare laparoscopic proficiency of trainees in obstetrics and gynaecology and general surgery using simulated tasks.
- The study's prospective design, robust data collection techniques including duplicate and blinded outcome assessment, and use of validated tools allowed us to minimise bias.
- The study reported effect sizes as well standard deviations and confidence intervals to allow the reader to assess the magnitude of study findings.
- The generalisability of the study can be enhanced if the study is repeated on a national or international scale.
- Larger comparative cohorts can provide more precision around the estimates of skill and allow adjustment for potential prognostic factors.

For peer review only

INTRODUCTION

The foundations of laparoscopic surgery were laid by gynaecologists and the first sterilisation procedure was performed laparoscopically in 1936.[1] Gynaecologists have led advancements in laparoscopy through innovation in laparoscopic instruments and educational tools such as the pelvic simulator trainer and Hasson's open technique for entry, which is widely used by general surgeons today. [1, 2]

Obstetrics and gynaecology (O&G) and general surgery (GS) trainees are required to demonstrate competency in different procedures, [3, 4] however, the core psychomotor skills required for laparoscopy are similar. Some of these skills include laparoscopic camera navigation (LCN), hand eye co-ordination (HEC) and bimanual co-ordination (BMC). Surgical trainees should be proficient in these skills early in their training to enable development of more complex and specific laparoscopic procedural techniques. [5, 6]

O&G training, lasting seven years, consists of basic (ST1-ST2), intermediate (ST3-ST5) and advanced training (ST6-ST7). The training covers both obstetrics and gynaecology although there is a significant focus on acquiring obstetric competencies throughout the training. [7] Exposure to laparoscopic surgery is gained only through gynaecological practice. Trainees who wish to pursue gynaecological training can select Advanced Training Skills Modules (ATSM) or subspecialisation relevant to gynaecological surgery in the advance part of the program. [8] In contrast, GS training is eight years long, including two years of core surgical training (CST1-2) and six years of higher surgical training (ST3-ST8), where the final two years focusses on subspecialty training (Figure 1). [4] GS trainees are required to be independent in laparoscopic appendicectomy by the end of CST2. [4] In contrast, O&G trainees are expected to perform 'minor operative laparoscopy' by the end of the fifth training year. [3] GS trainees, therefore, gain laparoscopic experience throughout their training programme whilst O&G trainees receive most of their laparoscopic surgery exposure in the advanced part of the program. [8, 9] The content of each stage of laparoscopic training in obstetrics and gynaecology and general surgery training is detailed in Table S1 and S2.

Opportunities for theatre experience appear to be lacking in both specialties. In 2021, the Royal College of Obstetricians and Gynaecologists (RCOG) evaluated the training of 1415 trainees and found that less than half of the ST5 and ST6 trainees reported adequate opportunities to develop the required surgical skills relevant to their stage of training.[10] Similarly, amongst 155 GS applicants certifying for completion of training, only two-thirds had reached the required number of cases. However, nearly three-quarters of these trainees had met the requirements for key procedures in their field. [11]

Our study compared the proficiency in core laparoscopic psychomotor skills amongst junior (ST3-ST5 in both specialties) and senior trainees (ST6-ST7 in O&G ST6-ST8 in GS) using a Karl Storz Szabo-Berci box trainer. We hypothesised that there is no difference in the performance of core laparoscopic skills between O&G and GS trainees at all training stages.

For peer review only

METHODS

Participants

47 trainees (24 O&G and 23 GS) from Health Education England North-West (HEENW) were invited to participate in this prospective observational study between September 2021 and April 2022. Trainees were allocated a study number, which was recognisable only to the two study investigators involved in the recruitment of trainees. To explore the effect of surgical experience, the trainees were sub-divided by their training grades into four groups: junior O&G, senior O&G, junior GS and senior GS.

The 'junior' group consisted of trainees between ST3 and ST5, and the 'senior' group included trainees in the final two years of O&G and GS training programs. For the senior O&G group, we selected trainees undertaking one of the advanced modules in 'advanced laparoscopy for the excision of benign disease', 'benign abdominal surgery - open and laparoscopic' and 'gynae-oncology'. This was to enable the selection of trainees in receipt of regular gynaecology theatre sessions and, therefore, comparable with GS seniors. Senior GS trainees with a specialist interest in breast surgery were excluded due to limited laparoscopic work within this subspecialty.

All participants provided written informed consent prior to participation. They completed a questionnaire collecting data on demographic details and factors relating to laparoscopic proficiency, such as the use of video games and laparoscopic simulators, attendance at courses involving laparoscopic surgery, training stage at first exposure to laparoscopic work, and the typical frequency of attendance in theatre.

The study was approved by the O&G and GS heads of schools from HEENW. Ethical approval was granted by the Faculty of Health and Medicine Research Ethics Committee at Lancaster University (FHMREC20033) and testing was conducted in accordance with the Declaration of Helsinki. Following ethical approval, the study was registered at clinicaltrials.gov (NCT05116332).

Patient and public involvement

No patient involved.

Procedures

All trainees were assessed by two faculty members/assessors in individual rooms to minimise external distractions. Assessors were not involved in the training of any study participants and trainees were able to discretely request a different assessor (s) if they knew the pre-assigned member or felt uncomfortable with them, without giving a reason. Trainee's specialty and training stage was concealed from the assessors to ensure anonymity of trainees and blinding of the assessors. Laparoscopic proficiency was measured by observing four standardised, simulated tasks using validated assessment tools. [5, 12-14] All trainees received the same written and video instructions explaining the task before beginning any assessments.[15] All tasks were performed on a Karl Storz Szabo-Berci-Sackier laparoscopic trainer. The first three tasks assessed core laparoscopic psychomotor skills using the Laparoscopic Skills Training and Testing (LASTT) model. [13] The fourth task evaluated laparoscopic suturing and was assessed using the suturing and knot tying training and testing (SUTT-1) method by the European Academy of Gynaecological Surgery. [16] Trainees performed each task three times, except for the suturing task, which was completed once. The rationale behind restricting repetition to three iterations was to familiarise trainees to the task so that their optimal performance could be elicited without inducing a significant rehearsal effect. [17]

For peer review only

The same equipment was used throughout the testing period for all trainees. All assessors received standardised training modified from the "Training the Trainers" of the Gynaecological Endoscopic Surgical Education and Assessment (GESEA) program. This consisted of an overview of all study tasks, instruments, scoring systems, and specifics details relating to set up and delivery of all the study tasks. Tasks were performed in order of increasing technical difficulty as described below.

Tasks

Task 1 - Laparoscopic Camera Navigation (LCN)

This task assessed the trainees' ability to navigate a 30° 10mm laparoscope to find 14 targets within the LASTT model. [12-14] The maximum time allowed was 300 seconds per iteration. A validation study on the LASTT model showed that the median time for task completion was 188 seconds for novices and ranged between 142 and 292 seconds. [12] O&G trainees use 30°telescope in hysteroscopic surgery and when using smaller laparoscopes. As the experience with using larger 30° laparoscopes may have been limited, we used the upper limit of the time range as the allocated time.

On the scoring sheet, the time taken to identify all 14 targets, or the last target identified within 300 seconds, was recorded. The task was considered successful when all 14 targets were identified in every iteration within the allocated timeframe. The trainees' best time (of the three iterations) was used to assess the speed of task completion. To assess the trainees' ability to integrate speed with navigation skills, the ratio of the total number of targets found to the total time taken to complete the task was calculated.

Task 2 – Hand eye co-ordination (HEC)

This task required the trainee to transfer six coloured cylinders to their respective coloured pins using a forceps in their dominant hand and navigating a 0° laparoscope with their non-dominant hand. [12-14] Time permitted for this task was 180 seconds per iteration. [12, 13]

Completion was determined when six cylinders were placed on their pins within the allocated time. The trainees' best time was used to calculate the speed of task completion. We recorded the total number of times a cylinder was dropped during each iteration. A sum of the three iterations gave a total number of drops. This was used as an indicator of precision of movement.

Task 3 – Bimanual Co-ordination (BMC)

This task assessed the trainees' ability to transfer six coloured pushpins between forceps in their dominant and non-dominant hands and place them in their coloured slots on the LASTT model. [12-14] The assessor navigated the camera for the trainees based on their instructions. A maximum of 180 seconds was allowed per iteration and outcome measures were the same as for HEC.

Task 4 – Laparoscopic suturing and intracorporeal knot placement (suturing)

A foam pad was used to assess suturing and knot placement using the SUTT-1 method. [16] All trainees were shown a 60-second video demonstration of laparoscopic suturing and intracorporeal knot tying to ensure that the instructions were standardised, and expectations were clearly understood. [18] Trainees were asked to place four interrupted sutures and perform four intracorporeal knots comprising of three throws. A maximum of 15 minutes was permitted for this task. The quality of suturing and knot-tying was assessed by two experienced consultants (one O&G and one GS consultant; both with over 10 years of experience in laparoscopic suturing) after completion of the task using a validated SUTT scoring system. [16] The assessors were blinded to the trainee and each other's score. All components of the total suturing score, such as extent of trauma, were scored after thorough inspection of the foam pads. The suturing task

For peer review only

was deemed complete if four horizontal sutures and four secure knots were secured within 15 minutes. The median number of sutures and knots inserted (out of four) and the total suturing scores were analysed.

A summary of the surgical tasks and their assessment are provided in Table 1.

Table 1. Summary of laparoscopic surgical tasks using a box trainer and methods

Task	Iterations	Time allocated	Data recorded	Outcome
**1 – Laparoscopic camera navigation (LCN)**	3	300 seconds	Time taken to find 14 targets<br>If exceeding 300 seconds, the last target found	Best time*<br>No of targets found
**2 – Hand eye co-ordination (HEC)**	3	180 seconds	Time taken<br>No. of objects placed<br>No. of drops	Best time*<br>Overall no. of drops**
**3 – Bimanual co-ordination (BMC)**	3	180 seconds	Time taken<br>No. of objects placed<br>No. of drops	Best time*<br>Overall no. of drops**
**4 – Suturing and intracorporeal knot placement (suturing)**	1	15 minutes	Time taken<br>Quality of sutures and knots	Median no. of sutures and knots<br>Total suturing scores

*shortest completion time out of three iterations.
**sum of dropped objects across the three iterations.

Statistical analysis

The Chi squared test (χ^2) was used to analyse demographic, training related variables between specialties (Table 2) and successful completion of all tasks. All continuous variables are reported as mean, standard deviation and 95% confidence intervals.

Normality was checked for tasks 1-3, including the LCN time and efficiency ratio, HEC time and precision score, and BMC time and precision score. As normality was only confirmed for BMC time, a robust ANOVA [19, 20] was used to compare the junior and senior trainee groups within the two specialties. The Holm-Bonferroni post hoc test was carried out to locate the difference and adjust for multiple comparisons when a significant result was observed. Where trainee's surgical experience did not have a significant effect, robust independent t-tests were used to compare differences between O&G and GS. Effect sizes (ξ) were calculated for all significant comparisons and 0.1 was considered small, 0.3 moderate and 0.5 large.[21] BMC time was analysed using ANOVA to compare junior and senior trainee groups within the two specialties and independent t-tests to assess differences between specialties. Holm-Bonferroni post hoc test was carried out to locate the difference and adjust for multiple comparisons when a significant result was observed.

For peer review only

In the suturing task, the number of sutures and knots were compared between the four groups using the Kruskal Wallis test, with Holm-Bonferroni correction for multiple pairwise comparisons. This data is reported as median and interquartile range. Hedges *g* was calculated for all significant comparisons with 0.2, 0.5 and 0.8 considered as small, moderate and large, respectively. [22] Agreement of total suturing scores between assessors was examined with Cronbach's alpha. [23] According to Bland and Altman, α =0.95 is desirable for clinical applications.[24] Total suturing scores were analysed using robust statistics as above. Statistical analysis was conducted in Jamovi Version 2.3.18.0 (The Jamovi project, https://www.jamovi.org) while collation and creation of figures was completed in GraphPad Prism v9 (GraphPad Software, San Diego, Calif., USA). Statistical significance was set at P≤0.05 and the corrected values are presented.

RESULTS

Participant Characteristics

Two trainees were excluded from the analysis as they did not meet the inclusion criteria (one senior O&G trainee) and had incomplete data (one senior GS trainee). 23 O&G trainees (mean ± SD, age 34 ± 4 years) and 22 GS trainees (34 ± 5 years) were selected for data analysis. The OG group consisted of 11 junior and 12 senior trainees and GS group consisted of 11 junior and 11 senior trainees. Both groups were not significantly different except their gender. Most O&G trainees were female in contrast to GS, where the majority were male.

Factors relating to proficiency in laparoscopic skills

Pre-testing baseline questionnaires showed that a significantly larger number of O&G trainees used a simulator than GS trainees; O&G 16 (70%) vs. GS 7 (32%), P =0.01. However, the number of trainees using the simulator frequently, such as once a month, was similar between the two specialties: O&G 3 (13%) vs. GS 2 (9%), P=0.32. O&G trainees reported attending significantly fewer elective and emergency laparoscopic theatre sessions; O&G 64 (37%) and 23 (19%) vs. GS 110 (63%) and 100 (81%), P <0.001 for both comparisons. However, analysis by training grade showed that senior O&G and senior GS trainees attended a similar number of elective sessions; O&G 51 (80%) vs. GS 56 (51%), P=0.30. Furthermore, junior O&G trainees were assigned to an assistant's role significantly more frequently than junior GS trainees; O&G 7 (64%) vs. GS 2 (18%), P =0.05 **(Table 2).**

For peer review only

Table 2. Laparoscopic training experience amongst O&G and GS trainees. Data is presented as frequencies (%). P values in bold indicate significant findings.

	O&G (n=23)	GS (n=22)	P
**Females**	15 (65%)	5 (13%)	**0.004**
**Males**	8 (35%)	17 (77%)	
**Juniors**	11 (48%)	11 (50%)	0.88
**Seniors**	12 (52%)	11 (50%)	
**Right handedness**	21 (91%)	19 (86%)	0.59
**Left/**ambidextrous	2 (9%)	3 (14%)	
**Played video games**	11 (48%)	8 (36%)	0.43
**Used pelvic simulator**	16 (70%)	7 (32%)	**0.01**
**Weekly**	2 (9%)	0 (0%)	
**Monthly**	3 (13%)	2 (9%)	0.32
**Less frequent**	18 (78%)	20 (91%)	
**Attended laparoscopic courses**	18 (78%)	20 (91%)	0.24
**Start of laparoscopic training:**			
**Core training**	14 (61%)	14 (67%)†	0.69
**Registrar training**	9 (39%)	7 (33%)	0.69
**Elective theatre sessions**	64 (37%)	110 (63%)	**<0.001**
**Junior**	13 (20%)	54 (49%)	**<0.001**
**Senior**	51 (80%)	56 (51%)	0.30
**Emergency theatre sessions/month**	23 (19%)	100 (81%)	**<0.001**
**Junior**	10 (43%)‡	46 (46%)	**0.003**
**Senior**	13 (57%)	54 (54%)	**<0.001**
**Type of exposure**			
**Juniors as Operator**	4 (36%)	9 (82%)	**0.03**
**Juniors as Assistant**	7 (64%)	2 (18%)	**0.03**
**Seniors as Operator**	10 (83%)	10 (91%)	0.59
**Seniors as Assistant**	2 (17%)	1 (9%)	0.59

† One junior GS trainee did not answer. ‡One junior O&G trainee did not answer.

Successful completion of tasks

Overall, O&G and GS trainees had 69 and 66 attempts at each of the three core tasks, respectively. A smaller number of attempts were successfully completed by O&G trainees in comparison to GS trainees on all three

tasks (LCN task: O&G 50 (72%) vs. GS 64 (97%), P <0.001; HEC task: O&G 54 (78%) vs. GS 64 (97%), P = 0.001; BMC task: O&G 47 (68%) vs. GS 62 (94%), P< 0.001).

Task Completion times (Speed)

There was a significant effect of specialty on completion times for LCN; F(3,33)= 6.26, P=0.005, HEC; F(3,33)=7.34, P=0.002, BMC; F(3,41)=11.6, P<0.001. Post hoc analyses showed significant differences between junior O&G and junior GS trainees only and no significant difference was found within the specialty groups, (i.e., between junior and senior trainees in either specialty). Between groups comparison showed that O&G specialty trainees were 73 seconds slower at completing LCN; O&G 166 ± 56, (139 to 193) seconds vs. GS 93 ± 21 (83 to 103) seconds, t(21)= 4.17, P<0.001, Effect size (ξ) = 0.76. O&G trainees were also significantly slower at HEC; O&G 105 ± 30 (90 to 119) seconds vs GS 67 ± 13 (60 to 73) seconds, t(25.6)=3.98, P<0.001, ξ= 0.66 and BMC task; O&G 139 ± 32 (125 to 153) seconds vs GS 100 ± 20 (92 to 109) seconds, t(43)= 4.74, P<0.001, ξ= 1.41. **(Figure 2a-c).**

Precision of movements (Accuracy)

Specialty had a significant effect on the precision of movements in LCN; F(3,33)=8.23, P=0.001, and BMC; F(3,33)=3.37, P=0.04. However, no significant difference was found in the precision of movements in HEC; F(3,33)= 0.96, P=0.43. Post hoc analysis showed that greater trainee experience did not significantly affect precision outcomes on these tasks. Therefore, the data was analysed by overall specialty. Overall, in LCN, O&G trainees found fewer targets, in the given time, than GS trainees; O&G 0.09 ± 0.04, (0.07 to 0.10) vs. GS 0.16 ± 0.03, (0.14 to 0.17), t(31.6)= 5.27, P<0.001, ξ= 0.82. In BMC, O&G trainees dropped a significantly greater number of pins than GS trainees; O&G 5.4 ± 2.3 (4.3 to 6.6) vs. GS 2.9 ± 1.7 (2.1 to 3.8), t(32.8)=3.03 P =0.005, ξ=0.53. O&G and GS trainees both dropped similar number of cylinders during HEC task; O&G 3.5 ± 2.7 (2.2 to 4.8) vs. GS 2.3 ± 1.6 (1.5 to 3.1), t(32.2)=1.23, P=0.22, ξ= 0.27. **(Figure 2 d-f).**

Suturing

The inter-rater agreement of the assessors on the suturing task was very high (Cronbach's alpha 0.98 for O&G and 0.97 for GS). One O&G trainee (4.3%) and eight GS trainees (36%) completed this task in time; P =0.007.

Number of inserted sutures and knots

Overall, O&G junior trainees were able to place fewer sutures and tie fewer intracorporeal knots than junior GS trainees (sutures: O&G 1 (1-1) vs. GS 4 (3-4), P = 0.005, Hedges *g* =0.98; knots: O&G 0 (0-1) vs. GS 2 (2-4), P = 0.005, *g*= 0.95). Senior O&G trainees tied significantly fewer knots than senior GS trainees (O&G 2.5 (1-3) vs. GS 4 (3-4), P = 0.03, *g* =0.51). However, senior trainees in O&G and GS groups placed similar number of sutures (O&G 3 (2-3) vs. GS 4 (3-4), P = 0.07, *g* =0.4).

Total suturing scores

O&G trainees had a significantly lower total suturing score than the GS trainees; F(3,33)=36.3, P <0.001). Post hoc analysis showed that junior O&G trainee's total suturing score was significantly lower than junior GS trainees; O&G 3.6 ± 2.1, (1.97 to 5.14) vs GS 14.9 ± 4.4 (11.5 to 18.3), P<0.001 and senior O&G trainees also scored lower than senior GS trainees; O&G 11.4 ± 4.4 (8.2 to 14.6) vs GS 16.8 ± 2.1 (15.2 to 18.4), P= 0.03. Senior O&G trainees had a significantly higher total suturing score than junior O&G trainees; Senior O&G 11.4 ± 4.4 (8.23 to 14.6) vs. Junior O&G 3.6 ± 2.1 (1.97 to 5.14), P = 0.004. Senior GS trainees, however,

For peer review only

scored like their junior colleagues; Senior GS 16.8 ± 2.1 (15.2 to 18.4) vs. Junior GS 14.9 ± 4.4 (11.5 to 18.3), P = 0.35 (**Figure 3).**

DISCUSSION

Principal findings

The acquisition of core laparoscopic skills depends on multiple factors including exposure to large volumes of laparoscopic procedures, [25] deliberate practice, [26] and structured simulation programs. [27] It is unknown whether the differing design of O&G and GS training leads to differential attainment of laparoscopic skills. Our study found that GS trainees performed better than O&G trainees in all tasks that measured core laparoscopic psychomotor skills. This may, in part, be due to the discrepancy in the volume of laparoscopic practice between the two specialties. Our baseline questionnaire showed that the average GS trainee attended the operating theatre almost three times as often as the average O&G trainee and were more likely to perform as the main operator in contrast to O&G trainees.

Our study found that increased training experience had an impact on suturing and knot tying but not on the other three core laparoscopic tasks. This may be due to the simplicity of these core tasks. Surgical skills such as navigating a camera and retracting surgical tissue are usually learnt early in the training and reach a plateau phase rather quickly. It has been confirmed that participants rapidly reached their optimal performance on simple tasks such as HEC and that despite further training no significant improvements were seen in performance. [5] Suturing, however, is regarded as a complex task and has been shown to improve with greater surgical experience. [28]

Meaning of the study: possible explanations and implications for clinicians and policymakers

Most of the emergency work in O&G relates to obstetrics and exposure to out-of-hours laparoscopic procedures is therefore limited. [29] Our study confirmed this. Overall, O&G trainees attended fewer laparoscopic theatre sessions and were less likely to be given the main operator's role than their GS counterparts. However, this difference was largely between the junior trainees only. Our baseline questionnaire showed that senior O&G trainees, in fact, attended a similar number of elective theatre sessions as the senior GS trainees and acted as the 'main operator' almost as frequently as the senior GS trainees. It appears that in O&G, theatre exposure and operative opportunities are concentrated in the latter part of the training. Psychological techniques have consistently shown that distributed practice is superior to concentrated practice and leads to the enhanced acquisition, consolidation, and retention of surgical skills. [30, 31] However, it remains unclear if the model of concentrated exposure in O&G may have contributed to the discrepancy in performance between the two specialties.

The Royal College of Obstetricians and Gynaecologists (RCOG) expects all senior (advanced) trainees to be independent in laparoscopic salpingectomy (a procedure used for removing tubal ectopic pregnancy). [3] However, senior trainees' competency in salpingectomy has been shown to vary between 32% and 89%. [32, 33] Based on feedback from O&G trainees, and documented benefits of distributed practice in learning new skills,[30, 31] introducing salpingectomy earlier in the O&G curriculum might be helpful. It may encourage hospitals to give trainees more surgical exposure from an earlier stage and trainees achieving

For peer review only

competency in this simple procedure may find it easier to learn more complex skills such as laparoscopic suturing. [5]

A greater number of O&G trainees reported using a pelvic simulator, however, only a minority reported using it frequently. Surgical skills learnt on simulators can be transferred to real patient surgery, but these benefits are mostly observed with repetitive practice and as part of a structured simulation program. [34-36] The latter is promoted as a solution for bridging the gap between required operative skills and reduced training opportunities. [37, 38] In this context the American College of Obstetricians & Gynaecologist have included a structured simulation program, as part of board certification for practice in O&G. [39]

Strengths and limitations of the study

To our knowledge, this is the first prospective study to examine trainees' laparoscopic skills in two surgical specialties who work in an anatomically similar environment. The training tools in this study were based on widely used and validated assessments, [12, 13] and our inter-observer reliability for the suturing assessments was very high. The two assessors were not involved with the individual participants' training, and they were blinded to the trainee's specialty, experience and to each other's scores.

This study was localised to the North-West region of the UK and testing it on a national level would provide more precision around the estimates of skill and enhance external validity.

The effect of training grade was only apparent in the suturing and knot tying exercise. The effect of training grade was only apparent in the suturing and knot tying exercise. In the original study validating LASTT model, the novices were predominantly students with little or no operative experience and the experts were specialists with significant experience in advance surgical procedures. So, although the original study showed significant differences between novices and experts,[12] our junior group was more experienced than their novices. Therefore, it is possible that such differences were not large enough between our groups.

Simulation practice can facilitate the acquisition of new surgical skills if used systemically and comprehensively. Only a minority of the trainees undertook regular simulation and as such it is unlikely to have had a significant effect on the study tasks. Nonetheless, the type of simulation practice in this study has not been recorded, and this is a limitation.

The sample size may appear small for an observational study. Nonetheless, there are no previous studies available examining a similar aspect, and due to the difficulties in estimating the minimum difference considered important in this context, a priori sample size estimation was not possible. Consequently, along with the mean and SD values, we also included CIs and effect sizes to enable future meta-analysis as well as inform readers of the precision and magnitude of the results.

Finally, the male: female ratio between the specialty groups was considerably different, probably reflecting the relevant population in each specialty. Although evidence points to lack of differences between male and female surgeons,[40, 41] future studies should aim to equate the participants based on sex, to alleviate any concerns around grouping male and female surgeons together.

Unanswered questions and future research

The validity of evaluating core psychomotor skills in laparoscopic surgery needs to be assessed against actual performance in the operating theatre. Our work showed that trainees with limited experience found

For peer review only

suturing (an actual surgical procedure) challenging but not the core psychomotor tasks. This implies that it is not just the mastery of core skills, but the cognitive and motor processes involved in applying these skills which may influence performance on actual surgical procedures. Therefore, future studies could look at cognitive and musculoskeletal stress amongst the two specialities and the seniority of its trainees.

Acknowledgements: We would like to thank Karl Storz, Medtronic, BSGE and BRIDGES society for their financial support with the study. We would also like to thank Mr Brice Rodriguez, Mr Kenn Ma, Dr Alison Sambrook, Mr Kenn Lim, Mr Mohamed Elsherbiny, Mr Mohamed Abdelrahman, Ms Christa Hammill, Ms Aleyamma Abraham, and Dr Nicholas Evans for their help with study protocol and conducting this study.

Funding: This study was funded by grants from the British Society of Gynaecological Endoscopy, Karl Storz and Medtronic.

Conflict of interest: None declared.

Author contributions: DAS and ZK conceived and developed the research idea. ZK, DS, DAS, CG, TJC and EH designed and implemented the study protocol. ZK, DS, AS, JEL, TJC, EH, KA, TB, CG and DAS conducted the study. TB, CG and ZK analysed the data. ZK, DS, AS, TJC, TB, CG, and DAS prepared the manuscript. All authors reviewed and approved the final manuscript.

Data statement: Data are available from the corresponding author (CG) upon reasonable request.

Ethics approval statement: Ethical approval was granted by the Faculty of Health and Medicine Research Ethics Committee at Lancaster University (FHMREC20033)

REFERENCES

1. Harrell AG, Heniford BT. Minimally invasive abdominal surgery: lux et veritas past, present, and future. The American Journal of Surgery. 2005;190(2):239-43.
2. Hasson H, Rotman C, Rana N, Kumari NA. Open Laparoscopy: 29- Years Experience. Obstet Gynecol. 2000;96:763-6.
3. Royal College of Obstetricians and Gynaecologists. Royal College of Obstetricians and Gynaecologists, Core Curriculum for Obstetrics and Gynaecology: RCOG; 2019 [updated June 2020. Available from: https://www.gmc-uk.org/-/media/documents/core-og-curriculum-2019-final-gmc-approved-20210504_pdf-79992893.pdf.
4. Intercollegiate surgical curriculum program (ISCP). Intercollegiate surgical curriculum program, Core Surgical Training Curriculum: ISCP; 2021 [updated Aug 2021. Available from: https://www.iscp.ac.uk/media/1326/core-surgical-training-curriculum-2021-minor-changes-for-august-2022.pdf.
5. Molinas CR, Campo R. Defining a structured training program for acquiring basic and advanced laparoscopic psychomotor skills in a simulator. Gynecological Surgery. 2010;7(4):427-35.
6. Molinas CR, Campo R. Retention of laparoscopic psychomotor skills after a structured training program depends on the quality of the training and on the complexity of the task. Gynecol Surg. 2016;13(4):395-402.
7. Royal College of Obstetricians and Gynaecologists. Royal College of Obstetricians and Gynaecologists, Training matrix London: RCOG; 2022 [Available from: https://www.rcog.org.uk/careers-and-training/starting-your-og-career/specialty-training/assessment-and-progression-through-training/training-matrix/.

For peer review only

8. Royal College of Obstetricians and Gynaecologists. Royal College of Obstetricians and Gynaecologists, Advanced Training in Obstetrics & Gynaecology: RCOG; 2019 [updated Aug 2019. Available from: https://www.rcog.org.uk/media/xigldqq1/advanced-training-definitive-document-2019.pdf.
9. McMurray R, Lawrence T, Afors K. Minimal access in gynaecological surgery: training the minimally invasive gynaecological surgeon. Obstetrics, Gynaecology & Reproductive Medicine. 2022;32(7):135-40.
10. Royal College of Obstetricians and Gynaecologists. Royal College of Obstetricians and Gynaecologists, Training Evaluation Feedback on Gynaecology training London: RCOG; 2021 [updated c2021. Available from: https://www.rcog.org.uk/media/vrkcsb4b/tef-2021-report-gynaecology-training.pdf.
11. Thomas C, Griffiths G, Abdelrahman T, Santos C, Lewis W. Does UK surgical training provide enough experience to meet today's training requirements? Bmj [Internet]. 2015 [cited 2022 October]; 350(h2503). Available from: https://www.bmj.com/content/350/bmj.h2503.
12. Molinas CR, De Win G, Ritter O, Keckstein J, Miserez M, Campo R. Feasibility and construct validity of a novel laparoscopic skills testing and training model. Gynecological surgery. 2008;5(4):281-90. eng.
13. Campo R, Reising C, Van Belle Y, Nassif J, O'Donovan P, Molinas CR. A valid model for testing and training laparoscopic psychomotor skills. Gynecological surgery. 2010;7(2):133-41. eng.
14. Campo R, Wattiez A, Leon De Wilde R, Molinas Sanabria CR. Training in laparoscopic surgery: From the lab to the or. Slovenian Journal of Public Health. 2012;51(4):285-98.
15. European Academy of Gynaecological Surgery. European Academy of Gynaecological Surgery, Laparoscopic Skills Training and Testing Method Europe: MiS Academy Europe; 2022 [Available from: https://europeanacademy.org/training-tools/lastt/.
16. Sleiman Z, Tanos V, Van Belle Y, Carvalho JL, Campo R. European Academy laparoscopic "Suturing Training and Testing'' significantly improves surgeons' performance. Facts Views Vis Obgyn. 2015;7(3):153-60. eng.
17. Feldman LS, Cao J, Andalib A, Fraser S, Fried GM. A method to characterize the learning curve for performance of a fundamental laparoscopic simulator task: defining "learning plateau" and "learning rate". Surgery. 2009;146(2):381-6.
18. LapPass Academy [online video]. Journal of surgical simulation. Laparoscopic Intracorporeal Suturing and Knot Tying, task 4. 1 video: 130 sec, HD, colour. UK: Journal of surgical simulation; 2020.
19. Field AP, Wilcox RR. Robust statistical methods: A primer for clinical psychology and experimental psychopathology researchers. Behav Res Ther. 2017;98:19-38.
20. Daszykowski M, Kaczmarek K, Vander Heyden Y, Walczak B. Robust statistics in data analysis — A review. Chemometrics and Intelligent Laboratory Systems. 2007;85(2):203-19.
21. Wilcox RR, Tian TS. Measuring effect size: a robust heteroscedastic approach for two or more groups. Journal of Applied Statistics. 2011;38(7):1359-68.
22. Brydges CR. Effect Size Guidelines, Sample Size Calculations, and Statistical Power in Gerontology. Innov Aging. 2019;3(4):1-8.
23. Tavakol M, Dennick R. Making sense of Cronbach's alpha. Int J Med Educ. 2011 Jun 27;2:53-5.
24. Bland JM, Altman DG. Statistics notes: Cronbach's alpha. BMJ [Internet]. 1997; 314(572).
25. Sadideen H, Alvand A, Saadeddin M, Kneebone R. Surgical experts: born or made? Int J Surg. 2013;11(9):773-8.
26. Reznick RK, MacRae H. Teaching Surgical Skills — Changes in the Wind. N Engl J Med. 2006;355(25):2664-9. eng.
27. Dawe SR, Pena GN, Windsor JA, Broeders JAJL, Cregan PC, Hewett PJ, et al. Systematic review of skills transfer after surgical simulation-based training. British Journal Of Surgery. 2014;101(9):pp1063-76. eng.
28. Train AT, Hu J, Narvaez JRF, Towle-Miller LM, Wilding GE, Cavuoto L, et al. Teaching surgery novices and trainees advanced laparoscopic suturing: a trial and tribulations. Surgical endoscopy. 2020;35(10):5816-26. eng.
29. Odejinmi F, Rizzuto I, Ballard KD. Potential barriers to the laparoscopic management of ectopic pregnancies: a regional UK study. Acta Obstet Gynecol Scand. 2010;89(10):1350-3. eng.
30. Cecilio-Fernandes D, Cnossen F, Jaarsma D, Tio RA. Avoiding Surgical Skill Decay: A Systematic Review on the Spacing of Training Sessions. J Surg Educ. 2018;75(2):471-80.
31. Gallagher AG, Jordan-Black JA, O'Sullivan GC. Prospective, randomized assessment of the acquisition, maintenance, and loss of laparoscopic skills. Ann Surg. 2012;256(2):387-93.
32. Moss EL, Bredaki FE, Jones PW, Hollingworth J, Luesley DM, Chan KK. Is gynaecological surgical training a cause for concern? A questionnaire survey of trainees and trainers. BMC Med Educ. 2011;11(1):32-. eng.

For peer review only

33. Christopoulos G, Kelly T, Lavery S, Trew G. Surgical skills of specialty trainees in emergency gynaecological laparoscopic procedures: a national UK survey. J Obstet Gynaecol. 2014;34(5):435-8.
34. Issenberg SB, McGaghie WC, Petrusa ER, Lee Gordon D, Scalese RJ. Features and uses of high-fidelity medical simulations that lead to effective learning: a BEME systematic review. Med Teach. 2005;27(1):10-28.
35. Gala R, Orejuela F, Gerten K, Lockrow E, Kilpatrick C, Chohan L, et al. Effect of validated skills simulation on operating room performance in obstetrics and gynecology residents: a randomized controlled trial. Obstet Gynecol. 2013;121(3):578-84.
36. Palter VN, Grantcharov TP. Development and validation of a comprehensive curriculum to teach an advanced minimally invasive procedure: a randomized controlled trial. Ann Surg. 2012;256(1):25-32.
37. Aggarwal R, Ward J, Balasundaram I, al. e. Proving the effectiveness of virtual reality simulation for training in laparoscopic surgery. Ann Surg. 2007;246(5):771-9.
38. Bryant-Smith A, Rymer J, Holland T, Brincat M. 'Perfect practice makes perfect': the role of laparoscopic simulation training in modern gynaecological training. The obstetrician & gynaecologist. 2020;22(1):69-74. eng.
39. American College of Obstetricians & Gynaecologists. American College of Obstetricians & Gynaecologists Washington: ACOG; 2022 [updated c2022. Available from: https://www.acog.org/education-and-events/creog/curriculum-resources/additional-curricular-resources/fundamentals-of-laparoscopic-surgery.
40. Ali A, Subhi Y, Ringsted C, Konge L. Gender differences in the acquisition of surgical skills: a systematic review. Surg Endosc. 2015;29(11):3065-73. eng.
41. Busshoff J, Datta RR, Bruns T, Kleinert R, Morgenstern B, Pfister D, et al. Gender benefit in laparoscopic surgical performance using a 3D-display system: data from a randomized cross-over trial. Surg Endosc. 2022 Jun;36(6):4376-85. PubMed PMID: 34750707. PMCID: PMC9085658. Epub 20211108.

Figure legends

Figure 1 - Outline of the training pathways in GS (a) and O&G (b). Adapted from the Royal College of Obstetricians & Gynaecologists (RCOG) (3) and intercollegiate surgical curriculum program (ISCP). (4)

Figure 2 - Time taken to complete laparoscopic tasks (a-c) and laparoscopic precision of movements by speciality (d-f). Task completion time for LCN (a), HEC (b) and BMC (c). Trainees' ability to integrate camera navigation skills with speed (d), the number of drops in HEC (e) and the number of drops in BMC (f). Data are presented as mean ± SD.

Figure 3 - Total suturing scores by trainee's experience within O&G and GS. Data are presented as mean ± SD.

For peer review only

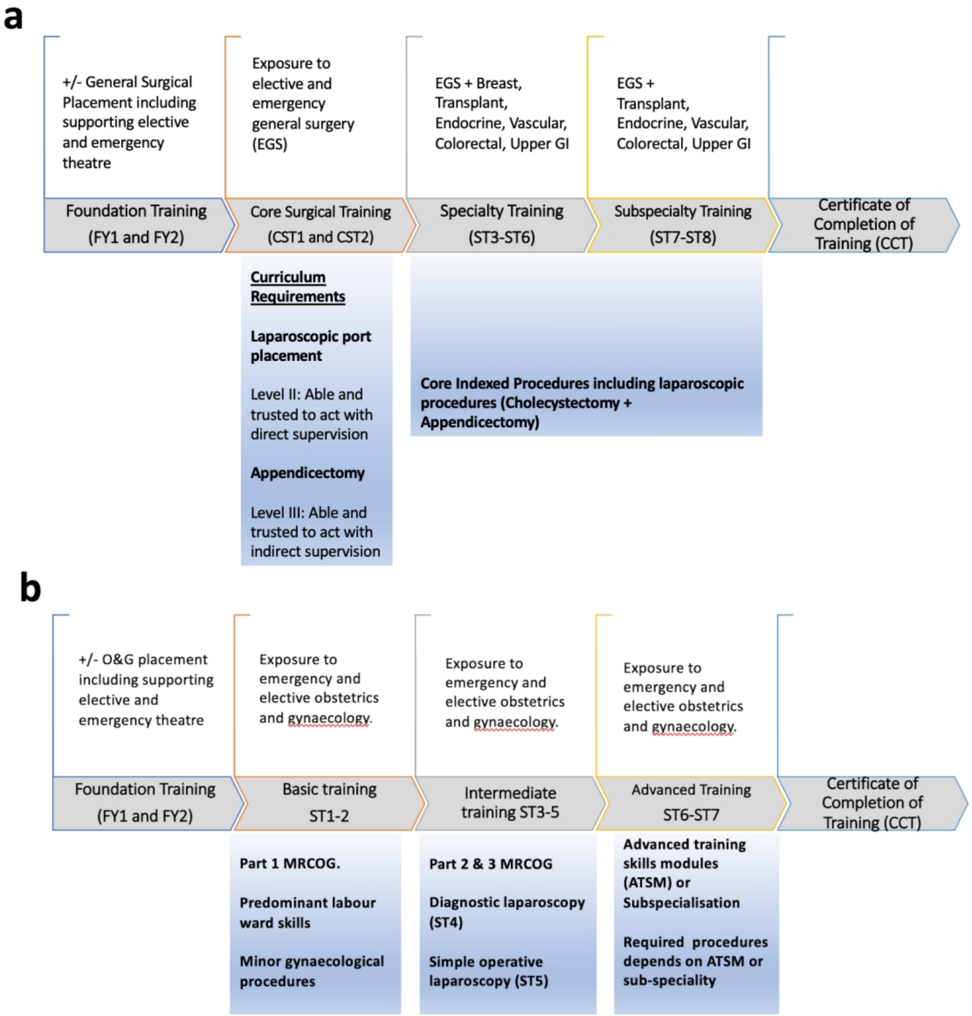

Figure 1: Outline of the training pathways in GS (a) and O&G (b). Adapted from the Royal College of Obstetricians & Gynaecologists (RCOG) (3) and intercollegiate surgical curriculum program (ISCP). (4)

140x145mm (300 x 300 DPI)

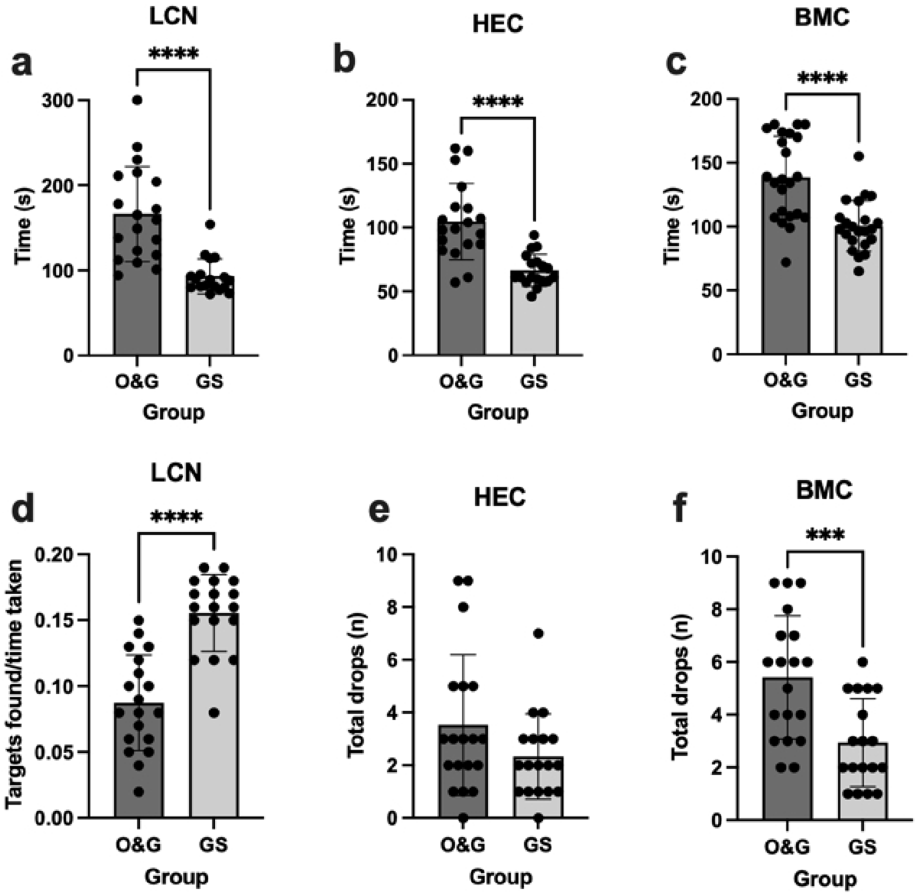

Figure 2. Time taken to complete laparoscopic tasks (a-c) and laparoscopic precision of movements by speciality (d-f). Task completion time for LCN (a), HEC (b) and BMC (c). Trainees' ability to integrate camera navigation skills with speed (d), the number of drops in HEC (e) and the number of drops in BMC (f). Data are presented as mean ± SD.

175x164mm (100 x 100 DPI)

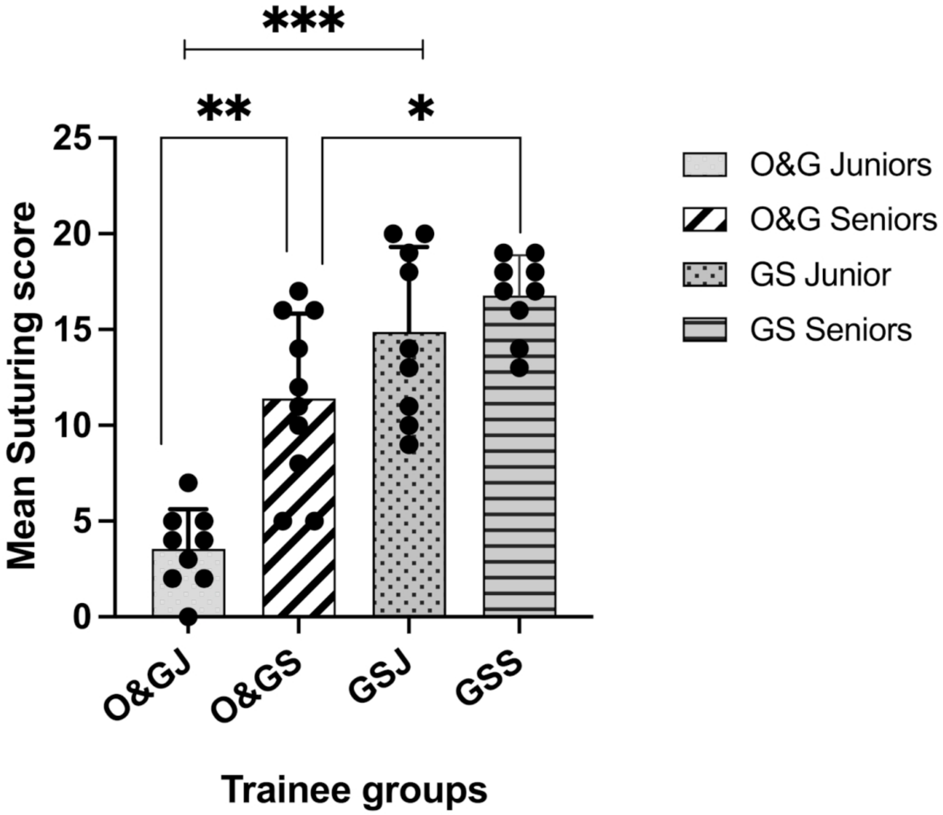

Figure 3. Total suturing scores by trainee’s experience within O&G and GS. Data are presented as mean ± SD.

128x108mm (300 x 300 DPI)

Table S1: Training matrix for O&G (adapted from RCOG). Required laparoscopic competencies are highlighted in **bold**. Competencies are signed off based on an entrustability scale* and as such no indicative numbers are included here.

	ST1	ST2	ST3	ST4	ST5	ST6	ST7
Curriculum	CiP progress appropriate to the relevant stage as per the CiP guides and entrustability levels.						
Examination	MRCOG Part 1			MRCOG Part 2 & Part 3			
At least 3 summative OSATS confirming competency by more than one assessor. At least one OSAT confirming competence should be supervised by a consultant.	Cervical smear	Caesarean section (Basic)<br><br>Non rotational assisted vaginal delivery (Ventouse & forceps)<br><br>Perineal repair<br><br>Surgical management of miscarriage/Surgical termination of pregnancy<br><br>Insertion of an Intrauterine system/intrauterine contraceptive device.<br><br>Endometrial biopsy	Manual removal of placenta<br><br>Transabdominal USS of early and late pregnancy	Hysteroscopy<br><br>**Diagnostic laparoscopy**<br><br>3rd degree perineal repair<br><br>Vulval biopsy	**Simple operative laparoscopy** (laparoscopic sterilisation or simple adnexal surgery e.g. adhesiolysis/ ovarian drilling<br><br>Caesarean section (Intermediate)<br><br>Rotational assisted vaginal delivery (any method)		Subspecialty specific competencies.<br><br>**Laparoscopic management of ectopic pregnancy**<br><br>**Ovarian cystectomy (laparoscopic & open)**<br><br>Surgical management of post partum haemorrhage

*Entrustability scale: 1= observe only, 2= direct supervision, 3= indirect supervision, 4= act independently with support, 5= act independently.

For peer review only

Table S2: Summary of required procedures in GS training. Indicative case/operative numbers are given for the specialty training phase where both the numbers and entrustability scales are used for assessment.

	CT1 Phase 1	CT2 Phase 1	ST3 Phase 2	ST4 Phase 2	ST5 Phase 2	ST6 Phase 2	ST7 Phase 3	ST8 Phase 3
Examinations		MRCS Part A<br>MRCS Part B						FRCS Part 1<br>FRCS Part 2
**Operative Requirements**<br>Level 1<br>Has observed<br>Level 2<br>Can do with assistance<br>Level 3<br>Can do whole but may need assistance<br>Level 4<br>Competent to do without assistance, including complications		Induction of pneumoperitoneum for laparoscopy with port placement (Level 2)<br>Appendicectomy (Level 3)<br>Open and close midline laparotomy incision (2)<br>Inguinal hernia repair (Level 2)<br>Primary abdominal wall hernia repair (Level 2)				Inguinal Hernia (level 4) [50 cases*]<br>Cholecystectomy (level 3) [40 cases*]<br>Segmental Colectomy (level 3) [15 cases*]<br>Emergency Laparotomy [45* cases]<br>Appendicectomy [60 cases*]		Emergency Laparotomy (Level 4) [100 cases*]<br>Appendicectomy (Level 4) [80 cases*]<br>Cholecystectomy [50 cases*] (level 4)<br>Segmental colectomy [20 cases*] (level 4)
**Other Operative Technical Skills**		Chest drain insertion (Level 3)<br>Needle biopsy including fine needle aspiration (Level 3)<br>Rigid sigmoidoscopy (Level 3)<br>Excision biopsy of benign skin or subcutaneous lesion (Level 4)						Indicative numbers and competencies for chosen specialty required. (Hepatopancreaticobiliary, Transplant, Endocrine, Colorectal, Oesophagogastric)

For peer review only

Reporting checklist for cross sectional study.

Based on the STROBE cross sectional guidelines.

Instructions to authors

Complete this checklist by entering the page numbers from your manuscript where readers will find each of the items listed below.

Your article may not currently address all the items on the checklist. Please modify your text to include the missing information. If you are certain that an item does not apply, please write "n/a" and provide a short explanation.

Upload your completed checklist as an extra file when you submit to a journal.

In your methods section, say that you used the STROBE cross sectionalreporting guidelines, and cite them as:

von Elm E, Altman DG, Egger M, Pocock SJ, Gotzsche PC, Vandenbroucke JP. The Strengthening the Reporting of Observational Studies in Epidemiology (STROBE) Statement: guidelines for reporting observational studies.

	Reporting Item	Page Number
**Title and abstract**		
Title	Indicate the study's design with a commonly used term in the title or the abstract	1
Abstract	Provide in the abstract an informative and balanced summary of what was done and what was found	2
**Introduction**		
Background / rationale	Explain the scientific background and rationale for the investigation being reported	3
Objectives	State specific objectives, including any prespecified hypotheses	3
**Methods**		
Study design	Present key elements of study design early in the paper	4
Setting	Describe the setting, locations, and relevant dates, including periods of recruitment, exposure, follow-up, and data collection	4

For peer review only

Eligibility criteria	Give the eligibility criteria, and the sources and methods of selection of participants.	4
	Clearly define all outcomes, exposures, predictors, potential confounders, and effect modifiers. Give diagnostic criteria, if applicable	5-6
Data sources / measurement	For each variable of interest give sources of data and details of methods of assessment (measurement). Describe comparability of assessment methods if there is more than one group. Give information separately for for exposed and unexposed groups if applicable.	5-6
Bias	Describe any efforts to address potential sources of bias	5-6
Study size	Explain how the study size was arrived at	Sample size not possible to calculate as no previous study on this topic to base clinically significant effect size, and standard deviation measures on.
Quantitative variables	Explain how quantitative variables were handled in the analyses. If applicable, describe which groupings were chosen, and why	6
Statistical methods	Describe all statistical methods, including those used to control for confounding	7
Statistical methods	Describe any methods used to examine subgroups and interactions	7
Statistical methods	Explain how missing data were addressed	7
Statistical methods	If applicable, describe analytical methods taking account of sampling strategy	NA
Statistical methods	Describe any sensitivity analyses	NA

Results

For peer review only

Participants	Report numbers of individuals at each stage of study—eg numbers potentially eligible, examined for eligibility, confirmed eligible, included in the study, completing follow-up, and analysed. Give information separately for for exposed and unexposed groups if applicable.	7
Participants	Give reasons for non-participation at each stage	7
Participants	Consider use of a flow diagram	NA
Descriptive data	Give characteristics of study participants (eg demographic, clinical, social) and information on exposures and potential confounders. Give information separately for exposed and unexposed groups if applicable.	8
Descriptive data	Indicate number of participants with missing data for each variable of interest	7-8
Outcome data	Report numbers of outcome events or summary measures. Give information separately for exposed and unexposed groups if applicable.	8-10
Main results	Give unadjusted estimates and, if applicable, confounder-adjusted estimates and their precision (eg, 95% confidence interval). Make clear which confounders were adjusted for and why they were included	7-10
Main results	Report category boundaries when continuous variables were categorized	9-10
Main results	If relevant, consider translating estimates of relative risk into absolute risk for a meaningful time period	No risks/RR reported
Other analyses	Report other analyses done—e.g., analyses of subgroups and interactions, and sensitivity analyses	9-10
**Discussion**		
Key results	Summarise key results with reference to study objectives	11
Limitations	Discuss limitations of the study, taking into account sources of potential bias or imprecision. Discuss both direction and magnitude of any potential bias.	12

For peer review only

Interpretation	Give a cautious overall interpretation considering objectives, limitations, multiplicity of analyses, results from similar studies, and other relevant evidence.	11-12
Generalisability	Discuss the generalisability (external validity) of the study results	12
**Other Information**		
Funding	Give the source of funding and the role of the funders for the present study and, if applicable, for the original study on which the present article is based	13

None The STROBE checklist is distributed under the terms of the Creative Commons Attribution License CC-BY. This checklist can be completed online using https://www.goodreports.org/, a tool made by the EQUATOR Network in collaboration with Penelope.ai

For peer review only

BMJ Open

A prospective observational study comparing proficiency of obstetrics & gynaecology trainees with general surgical trainees using simulated laparoscopic tasks

Journal:	*BMJ Open*
Manuscript ID	bmjopen-2023-075113.R2
Article Type:	Original research
Date Submitted by the Author:	02-Oct-2023
Complete List of Authors:	Khan, Zaibun N; Royal Lancaster Infirmary<br>Shrestha, Donna; Lancaster University<br>Shugaba, Abdulwarith; Lancaster University<br>Lambert, Joel E; Lancaster University<br>Clark, T; Birmingham Women's NHS Foundation Trust, Gynaecology; University of Birmingham,<br>Haslett, Elizabeth; Blackpool Victoria Hospital<br>Afors, Karolina; Whittington Health NHS Trust<br>Bampouras, Theodoros M; School of Sport and Exercise Sciences<br>Gaffney, Christopher; Lancaster University<br>Subar, Daren; East Lancashire Hospitals NHS Trust
**Primary Subject Heading**:	Surgery
Secondary Subject Heading:	Obstetrics and gynaecology, Medical education and training
Keywords:	OBSTETRICS, GYNAECOLOGY, MEDICAL EDUCATION & TRAINING

SCHOLARONE™
Manuscripts

I, the Submitting Author has the right to grant and does grant on behalf of all authors of the Work (as defined in the below author licence), an exclusive licence and/or a non-exclusive licence for contributions from authors who are: i) UK Crown employees; ii) where BMJ has agreed a CC-BY licence shall apply, and/or iii) in accordance with the terms applicable for US Federal Government officers or employees acting as part of their official duties; on a worldwide, perpetual, irrevocable, royalty-free basis to BMJ Publishing Group Ltd ("BMJ") its licensees and where the relevant Journal is co-owned by BMJ to the co-owners of the Journal, to publish the Work in this journal and any other BMJ products and to exploit all rights, as set out in our licence.

The Submitting Author accepts and understands that any supply made under these terms is made by BMJ to the Submitting Author unless you are acting as an employee on behalf of your employer or a postgraduate student of an affiliated institution which is paying any applicable article publishing charge ("APC") for Open Access articles. Where the Submitting Author wishes to make the Work available on an Open Access basis (and intends to pay the relevant APC), the terms of reuse of such Open Access shall be governed by a Creative Commons licence – details of these licences and which Creative Commons licence will apply to this Work are set out in our licence referred to above.

Other than as permitted in any relevant BMJ Author's Self Archiving Policies, I confirm this Work has not been accepted for publication elsewhere, is not being considered for publication elsewhere and does not duplicate material already published. I confirm all authors consent to publication of this Work and authorise the granting of this licence.

For peer review only

A prospective observational study comparing proficiency of obstetrics & gynaecology trainees with general surgical trainees using simulated laparoscopic tasks

Zaibun N Khan[1] (0000-0002-9558-8964), Donna Shrestha [2] (0000-0001-8711-3515), Abdulwarith Shugaba [2], Joel E Lambert [2] (0000-0001-5663-6284), T Justin Clark [3] (0000-0002-5943-1062), Elizabeth Haslett [4] (0000-0003-1165-0415), Karolina Afors [5], Theodoros M Bampouras [6] (0000-0002-8991-4655), Christopher J Gaffney [2]* (0000-0001-7990-2792), Daren A Subar [7].

Affiliations:

1. Royal Lancaster Infirmary, Ashton Rd, Lancaster, LA1 4RP, UK.

2. Lancaster Medical School, Lancaster University, Health Innovation One, Sir John Fisher Drive, Lancaster, LA1 4AT, UK.

3. Birmingham Women's Hospital, Birmingham Women's & Children's NHS Foundation Trust, Metchley Park Road, Edgbaston, Birmingham, B15 2TG, UK.

4. Blackpool Victoria Hospital, Whinney Heys Road, Blackpool, FY3 8NR.

5. Whittington Health NHS Trust, Magdala Avenue, London N19 5NF, UK.

6. Liverpool John Moores University, School of Sport and Exercise Sciences, Tom Reilly Building, Byrom Street, Liverpool, L3 3AF, UK.

7. East Lancashire Hospitals NHS Trust, Haslingden Road, Blackburn, BB2 3HH, UK.

Correspondence to: Dr Christopher J Gaffney, Lancaster Medical School, Lancaster University, Health Innovation One, Sir John Fisher Drive, Lancaster, LA1 4AT, UK; c.gaffney@lancaster.ac.uk; +44 (0) 1524 593 602

Word count: 3984

For peer review only

ABSTRACT

BACKGROUND: Training programmes for obstetrics and gynaecology (O&G) and general surgery (GS) vary significantly, but both require proficiency in laparoscopic skills. We sought to determine performance in each specialty.

DESIGN: Prospective, Observational study.

SETTING: Health Education England North-West, UK.

PARTICIPANTS: 47 surgical trainees (24 O&G and 23 GS) were sub-divided into four groups: 11 junior O&G, 13 senior O&G, 11 junior GS, and 12 senior GS trainees.

OBJECTIVES: Trainees were tested on four simulated laparoscopic tasks; laparoscopic camera navigation (LCN), hand eye co-ordination (HEC), bimanual co-ordination (BMC) and suturing with intracorporeal knot tying (suturing).

RESULTS: O&G trainees completed LCN (P <0.001), HEC (P <0.001) and BMC (P <0.001) significantly slower than GS trainees. Furthermore, O&G found fewer number of targets in LCN (P =0.001) and dropped a greater number of pins than the GS trainees in BMC (P =0.04). In all three tasks, there were significant differences between O&G and GS trainees but no difference between the juniors and senior groups within each specialty. Performance in suturing also varied by specialty; senior O&G trainees scored significantly lower than senior GS trainees; O&G 11.4 ± 4.4 vs GS 16.8 ± 2.1, P = 0.03. Whilst suturing scores improved with seniority among O&G trainees, there was no difference between the junior and senior GS trainees; senior O&G 11.4 ± 4.4 vs junior O&G 3.6 ± 2.1, P = 0.004.

DISCUSSION: GS trainees performed better than O&G trainees in core laparoscopic skills and the structure of obstetrics and gynaecology training may require modification.

Keywords: Laparoscopy; obstetrics; gynaecology; surgical training

For peer review only

Strengths and Limitations

- The study's prospective design, robust data collection techniques including duplicate and blinded outcome assessment, and use of validated tools allowed us to minimise bias.
- The study reported effect sizes as well standard deviations and confidence intervals to allow the reader to assess the magnitude of study findings.
- The generalisability of the study can be enhanced if the study is repeated on a national or international scale.
- Larger comparative cohorts can provide more precision around the estimates of skill and allow adjustment for potential prognostic factors.

For peer review only

INTRODUCTION

The foundations of laparoscopic surgery were laid by gynaecologists and the first sterilisation procedure was performed laparoscopically in 1936.[1] Gynaecologists have led advancements in laparoscopy through innovation in laparoscopic instruments and educational tools such as the pelvic simulator trainer and Hasson's open technique for entry, which is widely used by general surgeons today. [1, 2]

Obstetrics and gynaecology (O&G) and general surgery (GS) trainees are required to demonstrate competency in different procedures, [3, 4] however, the core psychomotor skills required for laparoscopy are similar. Some of these skills include laparoscopic camera navigation (LCN), hand eye co-ordination (HEC) and bimanual co-ordination (BMC). Surgical trainees should be proficient in these skills early in their training to enable development of more complex and specific laparoscopic procedural techniques. [5, 6]

O&G training, lasting seven years, consists of basic (ST1-ST2), intermediate (ST3-ST5) and advanced training (ST6-ST7). The training covers both obstetrics and gynaecology although there is a significant focus on acquiring obstetric competencies throughout the training. [7] Exposure to laparoscopic surgery is gained only through gynaecological practice. Trainees who wish to pursue gynaecological training can select Advanced Training Skills Modules (ATSM) or subspecialisation relevant to gynaecological surgery in the advance part of the program. [8] In contrast, GS training is eight years long, including two years of core surgical training (CST1-2) and six years of higher surgical training (ST3-ST8), where the final two years focusses on subspecialty training (Figure 1). [4] GS trainees are required to be independent in laparoscopic appendicectomy by the end of CST2. [4] In contrast, O&G trainees are expected to perform 'minor operative laparoscopy' by the end of the fifth training year. [3] GS trainees, therefore, gain laparoscopic experience throughout their training programme whilst O&G trainees receive most of their laparoscopic surgery exposure in the advanced part of the program. [8, 9] The content of each stage of laparoscopic training in obstetrics and gynaecology and general surgery training is detailed in Table S1 and S2.

Opportunities for theatre experience appear to be lacking in both specialties. In 2021, the Royal College of Obstetricians and Gynaecologists (RCOG) evaluated the training of 1415 trainees and found that less than half of the ST5 and ST6 trainees reported adequate opportunities to develop the required surgical skills relevant to their stage of training.[10] Similarly, amongst 155 GS applicants certifying for completion of training, only two-thirds had reached the required number of cases. However, nearly three-quarters of these trainees had met the requirements for key procedures in their field. [11]

Our study compared the proficiency in core laparoscopic psychomotor skills amongst junior (ST3-ST5 in both specialties) and senior trainees (ST6-ST7 in O&G ST6-ST8 in GS) using a Karl Storz Szabo-Berci box trainer. We hypothesised that there is no difference in the performance of core laparoscopic skills between O&G and GS trainees at all training stages.

For peer review only

METHODS

Participants

47 trainees (24 O&G and 23 GS) from Health Education England North-West (HEENW) were invited to participate in this prospective observational study between September 2021 and April 2022. Trainees were allocated a study number, which was recognisable only to the two study investigators involved in the recruitment of trainees. To explore the effect of surgical experience, the trainees were sub-divided by their training grades into four groups: junior O&G, senior O&G, junior GS and senior GS.

The 'junior' group consisted of trainees between ST3 and ST5, and the 'senior' group included trainees in the final two years of O&G and GS training programs. For the senior O&G group, we selected trainees undertaking one of the advanced modules in 'advanced laparoscopy for the excision of benign disease', 'benign abdominal surgery - open and laparoscopic' and 'gynae-oncology'. This was to enable the selection of trainees in receipt of regular gynaecology theatre sessions and, therefore, comparable with GS seniors. Senior GS trainees with a specialist interest in breast surgery were excluded due to limited laparoscopic work within this subspecialty.

All participants provided written informed consent prior to participation. They completed a questionnaire collecting data on demographic details and factors relating to laparoscopic proficiency, such as the use of video games and laparoscopic simulators, attendance at courses involving laparoscopic surgery, training stage at first exposure to laparoscopic work, and the typical frequency of attendance in theatre.

The study was approved by the O&G and GS heads of schools from HEENW. Ethical approval was granted by the Faculty of Health and Medicine Research Ethics Committee at Lancaster University (FHMREC20033) and testing was conducted in accordance with the Declaration of Helsinki. Following ethical approval, the study was registered at clinicaltrials.gov (NCT05116332).

Patient and public involvement

No patient involved.

Procedures

All trainees were assessed by two faculty members/assessors in individual rooms to minimise external distractions. Assessors were not involved in the training of any study participants and trainees were able to discretely request a different assessor (s) if they knew the pre-assigned member or felt uncomfortable with them, without giving a reason. Trainee's specialty and training stage was concealed from the assessors to ensure anonymity of trainees and blinding of the assessors. Laparoscopic proficiency was measured by observing four standardised, simulated tasks using validated assessment tools. [5, 12-14] All trainees received the same written and video instructions explaining the task before beginning any assessments.[15] All tasks were performed on a Karl Storz Szabo-Berci-Sackier laparoscopic trainer. The first three tasks assessed core laparoscopic psychomotor skills using the Laparoscopic Skills Training and Testing (LASTT) model. [13] The fourth task evaluated laparoscopic suturing and was assessed using the suturing and knot tying training and testing (SUTT-1) method by the European Academy of Gynaecological Surgery. [16] Trainees performed each task three times, except for the suturing task, which was completed once. The rationale behind restricting repetition to three iterations was to familiarise trainees to the task so that their optimal performance could be elicited without inducing a significant rehearsal effect. [17]

For peer review only - http://bmjopen.bmj.com/site/about/guidelines.xhtml

The same equipment was used throughout the testing period for all trainees. All assessors received standardised training modified from the "Training the Trainers" of the Gynaecological Endoscopic Surgical Education and Assessment (GESEA) program. This consisted of an overview of all study tasks, instruments, scoring systems, and specifics details relating to set up and delivery of all the study tasks. Tasks were performed in order of increasing technical difficulty as described below.

Tasks

Task 1 - Laparoscopic Camera Navigation (LCN)

This task assessed the trainees' ability to navigate a 30° 10mm laparoscope to find 14 targets within the LASTT model. [12-14] The maximum time allowed was 300 seconds per iteration. A validation study on the LASTT model showed that the median time for task completion was 188 seconds for novices and ranged between 142 and 292 seconds. [12] O&G trainees use 30°telescope in hysteroscopic surgery and when using smaller laparoscopes. As the experience with using larger 30° laparoscopes may have been limited, we used the upper limit of the time range as the allocated time.

On the scoring sheet, the time taken to identify all 14 targets, or the last target identified within 300 seconds, was recorded. The task was considered successful when all 14 targets were identified in every iteration within the allocated timeframe. The trainees' best time (of the three iterations) was used to assess the speed of task completion. To assess the trainees' ability to integrate speed with navigation skills, the ratio of the total number of targets found to the total time taken to complete the task was calculated.

Task 2 – Hand eye co-ordination (HEC)

This task required the trainee to transfer six coloured cylinders to their respective coloured pins using a forceps in their dominant hand and navigating a 0° laparoscope with their non-dominant hand. [12-14] Time permitted for this task was 180 seconds per iteration. [12, 13]

Completion was determined when six cylinders were placed on their pins within the allocated time. The trainees' best time was used to calculate the speed of task completion. We recorded the total number of times a cylinder was dropped during each iteration. A sum of the three iterations gave a total number of drops. This was used as an indicator of precision of movement.

Task 3 – Bimanual Co-ordination (BMC)

This task assessed the trainees' ability to transfer six coloured pushpins between forceps in their dominant and non-dominant hands and place them in their coloured slots on the LASTT model. [12-14] The assessor navigated the camera for the trainees based on their instructions. A maximum of 180 seconds was allowed per iteration and outcome measures were the same as for HEC.

Task 4 – Laparoscopic suturing and intracorporeal knot placement (suturing)

A foam pad was used to assess suturing and knot placement using the SUTT-1 method. [16] All trainees were shown a 60-second video demonstration of laparoscopic suturing and intracorporeal knot tying to ensure that the instructions were standardised, and expectations were clearly understood. [18] Trainees were asked to place four interrupted sutures and perform four intracorporeal knots comprising of three throws. A maximum of 15 minutes was permitted for this task. The quality of suturing and knot-tying was assessed by two experienced consultants (one O&G and one GS consultant; both with over 10 years of experience in laparoscopic suturing) after completion of the task using a validated SUTT scoring system. [16] The assessors were blinded to the trainee and each other's score. All components of the total suturing score, such as extent of trauma, were scored after thorough inspection of the foam pads. The suturing task

For peer review only

was deemed complete if four horizontal sutures and four secure knots were secured within 15 minutes. The median number of sutures and knots inserted (out of four) and the total suturing scores were analysed.

A summary of the surgical tasks and their assessment are provided in Table 1.

Table 1. Summary of laparoscopic surgical tasks using a box trainer and methods

Task	Iterations	Time allocated	Data recorded	Outcome
**1 – Laparoscopic camera navigation (LCN)**	3	300 seconds	Time taken to find 14 targets<br>If exceeding 300 seconds, the last target found	Best time*<br>No of targets found
**2 – Hand eye co-ordination (HEC)**	3	180 seconds	Time taken<br>No. of objects placed<br>No. of drops	Best time*<br>Overall no. of drops**
**3 – Bimanual co-ordination (BMC)**	3	180 seconds	Time taken<br>No. of objects placed<br>No. of drops	Best time*<br>Overall no. of drops**
**4 – Suturing and intracorporeal knot placement (suturing)**	1	15 minutes	Time taken<br>Quality of sutures and knots	Median no. of sutures and knots<br>Total suturing scores

*shortest completion time out of three iterations.
**sum of dropped objects across the three iterations.

Statistical analysis

The Chi squared test (χ^2) was used to analyse demographic, training related variables between specialties (Table 2) and successful completion of all tasks. All continuous variables are reported as mean, standard deviation and 95% confidence intervals.

Normality was checked for tasks 1-3, including the LCN time and efficiency ratio, HEC time and precision score, and BMC time and precision score. As normality was only confirmed for BMC time, a robust ANOVA [19, 20] was used to compare the junior and senior trainee groups within the two specialties. The Holm-Bonferroni post hoc test was carried out to locate the difference and adjust for multiple comparisons when a significant result was observed. Where trainee's surgical experience did not have a significant effect, robust independent t-tests were used to compare differences between O&G and GS. Effect sizes (ξ) were calculated for all significant comparisons and 0.1 was considered small, 0.3 moderate and 0.5 large.[21] BMC time was analysed using ANOVA to compare junior and senior trainee groups within the two specialties and independent t-tests to assess differences between specialties. Holm-Bonferroni post hoc test was carried out to locate the difference and adjust for multiple comparisons when a significant result was observed.

For peer review only

In the suturing task, the number of sutures and knots were compared between the four groups using the Kruskal Wallis test, with Holm-Bonferroni correction for multiple pairwise comparisons. This data is reported as median and interquartile range. Hedges *g* was calculated for all significant comparisons with 0.2, 0.5 and 0.8 considered as small, moderate and large, respectively. [22] Agreement of total suturing scores between assessors was examined with Cronbach's alpha. [23] According to Bland and Altman, α =0.95 is desirable for clinical applications.[24] Total suturing scores were analysed using robust statistics as above. Statistical analysis was conducted in Jamovi Version 2.3.18.0 (The Jamovi project, https://www.jamovi.org) while collation and creation of figures was completed in GraphPad Prism v9 (GraphPad Software, San Diego, Calif., USA). Statistical significance was set at P≤0.05 and the corrected values are presented.

RESULTS

Participant Characteristics

Two trainees were excluded from the analysis as they did not meet the inclusion criteria (one senior O&G trainee) and had incomplete data (one senior GS trainee). 23 O&G trainees (mean ± SD, age 34 ± 4 years) and 22 GS trainees (34 ± 5 years) were selected for data analysis. The OG group consisted of 11 junior and 12 senior trainees and GS group consisted of 11 junior and 11 senior trainees. Both groups were not significantly different except their gender. Most O&G trainees were female in contrast to GS, where the majority were male.

Factors relating to proficiency in laparoscopic skills

Pre-testing baseline questionnaires showed that a significantly larger number of O&G trainees used a simulator than GS trainees; O&G 16 (70%) vs. GS 7 (32%), P =0.01. However, the number of trainees using the simulator frequently, such as once a month, was similar between the two specialties: O&G 3 (13%) vs. GS 2 (9%), P=0.32. O&G trainees reported attending significantly fewer elective and emergency laparoscopic theatre sessions; O&G 64 (37%) and 23 (19%) vs. GS 110 (63%) and 100 (81%), P <0.001 for both comparisons. However, analysis by training grade showed that senior O&G and senior GS trainees attended a similar number of elective sessions; O&G 51 (80%) vs. GS 56 (51%), P=0.30. Furthermore, junior O&G trainees were assigned to an assistant's role significantly more frequently than junior GS trainees; O&G 7 (64%) vs. GS 2 (18%), P =0.05 **(Table 2).**

For peer review only

Table 2. Laparoscopic training experience amongst O&G and GS trainees. Data is presented as frequencies (%). P values in bold indicate significant findings.

	O&G (n=23)	GS (n=22)	P
**Females**	15 (65%)	5 (13%)	**0.004**
**Males**	8 (35%)	17 (77%)	
**Juniors**	11 (48%)	11 (50%)	0.88
**Seniors**	12 (52%)	11 (50%)	
**Right handedness**	21 (91%)	19 (86%)	0.59
**Left/**ambidextrous	2 (9%)	3 (14%)	
**Played video games**	11 (48%)	8 (36%)	0.43
**Used pelvic simulator**	16 (70%)	7 (32%)	**0.01**
**Weekly**	2 (9%)	0 (0%)	
**Monthly**	3 (13%)	2 (9%)	0.32
**Less frequent**	18 (78%)	20 (91%)	
**Attended laparoscopic courses**	18 (78%)	20 (91%)	0.24
**Start of laparoscopic training:**			
**Core training**	14 (61%)	14 (67%)†	0.69
**Registrar training**	9 (39%)	7 (33%)	0.69
**Elective theatre sessions**	64 (37%)	110 (63%)	**<0.001**
**Junior**	13 (20%)	54 (49%)	**<0.001**
**Senior**	51 (80%)	56 (51%)	0.30
**Emergency theatre sessions/month**	23 (19%)	100 (81%)	**<0.001**
**Junior**	10 (43%)‡	46 (46%)	**0.003**
**Senior**	13 (57%)	54 (54%)	**<0.001**
**Type of exposure**			
**Juniors as Operator**	4 (36%)	9 (82%)	**0.03**
**Juniors as Assistant**	7 (64%)	2 (18%)	**0.03**
**Seniors as Operator**	10 (83%)	10 (91%)	0.59
**Seniors as Assistant**	2 (17%)	1 (9%)	0.59

† One junior GS trainee did not answer. ‡One junior O&G trainee did not answer.

Successful completion of tasks

Overall, O&G and GS trainees had 69 and 66 attempts at each of the three core tasks, respectively. A smaller number of attempts were successfully completed by O&G trainees in comparison to GS trainees on all three

tasks (LCN task: O&G 50 (72%) vs. GS 64 (97%), P <0.001; HEC task: O&G 54 (78%) vs. GS 64 (97%), P = 0.001; BMC task: O&G 47 (68%) vs. GS 62 (94%), P< 0.001).

Task Completion times (Speed)

There was a significant effect of specialty on completion times for LCN; F(3,33)= 6.26, P=0.005, HEC; F(3,33)=7.34, P=0.002, BMC; F(3,41)=11.6, P<0.001. Post hoc analyses showed significant differences between junior O&G and junior GS trainees only and no significant difference was found within the specialty groups, (i.e., between junior and senior trainees in either specialty). Between groups comparison showed that O&G specialty trainees were 73 seconds slower at completing LCN; O&G 166 ± 56, (139 to 193) seconds vs. GS 93 ± 21 (83 to 103) seconds, t(21)= 4.17, P<0.001, Effect size (ξ) = 0.76. O&G trainees were also significantly slower at HEC; O&G 105 ± 30 (90 to 119) seconds vs GS 67 ± 13 (60 to 73) seconds, t(25.6)=3.98, P<0.001, ξ= 0.66 and BMC task; O&G 139 ± 32 (125 to 153) seconds vs GS 100 ± 20 (92 to 109) seconds, t(43)= 4.74, P<0.001, ξ= 1.41. **(Figure 2a-c).**

Precision of movements (Accuracy)

Specialty had a significant effect on the precision of movements in LCN; F(3,33)=8.23, P=0.001, and BMC; F(3,33)=3.37, P=0.04. However, no significant difference was found in the precision of movements in HEC; F(3,33)= 0.96, P=0.43. Post hoc analysis showed that greater trainee experience did not significantly affect precision outcomes on these tasks. Therefore, the data was analysed by overall specialty. Overall, in LCN, O&G trainees found fewer targets, in the given time, than GS trainees; O&G 0.09 ± 0.04, (0.07 to 0.10) vs. GS 0.16 ± 0.03, (0.14 to 0.17), t(31.6)= 5.27, P<0.001, ξ= 0.82. In BMC, O&G trainees dropped a significantly greater number of pins than GS trainees; O&G 5.4 ± 2.3 (4.3 to 6.6) vs. GS 2.9 ± 1.7 (2.1 to 3.8), t(32.8)=3.03 P =0.005, ξ=0.53. O&G and GS trainees both dropped similar number of cylinders during HEC task; O&G 3.5 ± 2.7 (2.2 to 4.8) vs. GS 2.3 ± 1.6 (1.5 to 3.1), t(32.2)=1.23, P=0.22, ξ= 0.27. **(Figure 2 d-f).**

Suturing

The inter-rater agreement of the assessors on the suturing task was very high (Cronbach's alpha 0.98 for O&G and 0.97 for GS). One O&G trainee (4.3%) and eight GS trainees (36%) completed this task in time; P =0.007.

Number of inserted sutures and knots

Overall, O&G junior trainees were able to place fewer sutures and tie fewer intracorporeal knots than junior GS trainees (sutures: O&G 1 (1-1) vs. GS 4 (3-4), P = 0.005, Hedges *g* =0.98; knots: O&G 0 (0-1) vs. GS 2 (2-4), P = 0.005, *g*= 0.95). Senior O&G trainees tied significantly fewer knots than senior GS trainees (O&G 2.5 (1-3) vs. GS 4 (3-4), P = 0.03, *g* =0.51). However, senior trainees in O&G and GS groups placed similar number of sutures (O&G 3 (2-3) vs. GS 4 (3-4), P = 0.07, *g* =0.4).

Total suturing scores

O&G trainees had a significantly lower total suturing score than the GS trainees; F(3,33)=36.3, P <0.001). Post hoc analysis showed that junior O&G trainee's total suturing score was significantly lower than junior GS trainees; O&G 3.6 ± 2.1, (1.97 to 5.14) vs GS 14.9 ± 4.4 (11.5 to 18.3), P<0.001 and senior O&G trainees also scored lower than senior GS trainees; O&G 11.4 ± 4.4 (8.2 to 14.6) vs GS 16.8 ± 2.1 (15.2 to 18.4), P= 0.03. Senior O&G trainees had a significantly higher total suturing score than junior O&G trainees; Senior O&G 11.4 ± 4.4 (8.23 to 14.6) vs. Junior O&G 3.6 ± 2.1 (1.97 to 5.14), P = 0.004. Senior GS trainees, however,

For peer review only

scored like their junior colleagues; Senior GS 16.8 ± 2.1 (15.2 to 18.4) vs. Junior GS 14.9 ± 4.4 (11.5 to 18.3), P = 0.35 (**Figure 3).**

DISCUSSION

Principal findings

The acquisition of core laparoscopic skills depends on multiple factors including exposure to large volumes of laparoscopic procedures, [25] deliberate practice, [26] and structured simulation programs. [27] It is unknown whether the differing design of O&G and GS training leads to differential attainment of laparoscopic skills. Our study found that GS trainees performed better than O&G trainees in all tasks that measured core laparoscopic psychomotor skills. This may, in part, be due to the discrepancy in the volume of laparoscopic practice between the two specialties. Our baseline questionnaire showed that the average GS trainee attended the operating theatre almost three times as often as the average O&G trainee and were more likely to perform as the main operator in contrast to O&G trainees.

Our study found that increased training experience had an impact on suturing and knot tying but not on the other three core laparoscopic tasks. This may be due to the simplicity of these core tasks. Surgical skills such as navigating a camera and retracting surgical tissue are usually learnt early in the training and reach a plateau phase rather quickly. It has been confirmed that participants rapidly reached their optimal performance on simple tasks such as HEC and that despite further training no significant improvements were seen in performance. [5] Suturing, however, is regarded as a complex task and has been shown to improve with greater surgical experience. [28]

Meaning of the study: possible explanations and implications for clinicians and policymakers

Most of the emergency work in O&G relates to obstetrics and exposure to out-of-hours laparoscopic procedures is therefore limited. [29] Our study confirmed this. Overall, O&G trainees attended fewer laparoscopic theatre sessions and were less likely to be given the main operator's role than their GS counterparts. However, this difference was largely between the junior trainees only. Our baseline questionnaire showed that senior O&G trainees, in fact, attended a similar number of elective theatre sessions as the senior GS trainees and acted as the 'main operator' almost as frequently as the senior GS trainees. It appears that in O&G, theatre exposure and operative opportunities are concentrated in the latter part of the training. Psychological techniques have consistently shown that distributed practice is superior to concentrated practice and leads to the enhanced acquisition, consolidation, and retention of surgical skills. [30, 31] However, it remains unclear if the model of concentrated exposure in O&G may have contributed to the discrepancy in performance between the two specialties.

The Royal College of Obstetricians and Gynaecologists (RCOG) expects all senior (advanced) trainees to be independent in laparoscopic salpingectomy (a procedure used for removing tubal ectopic pregnancy). [3] However, senior trainees' competency in salpingectomy has been shown to vary between 32% and 89%. [32, 33] Based on feedback from O&G trainees, and documented benefits of distributed practice in learning new skills,[30, 31] introducing salpingectomy earlier in the O&G curriculum might be helpful. It may encourage hospitals to give trainees more surgical exposure from an earlier stage and trainees achieving

competency in this simple procedure may find it easier to learn more complex skills such as laparoscopic suturing. [5]

A greater number of O&G trainees reported using a pelvic simulator, however, only a minority reported using it frequently. Surgical skills learnt on simulators can be transferred to real patient surgery, but these benefits are mostly observed with repetitive practice and as part of a structured simulation program. [34-36] The latter is promoted as a solution for bridging the gap between required operative skills and reduced training opportunities. [37, 38] In this context the American College of Obstetricians & Gynaecologist have included a structured simulation program, as part of board certification for practice in O&G. [39]

Strengths and limitations of the study

To our knowledge, this is the first prospective study to examine trainees' laparoscopic skills in two surgical specialties who work in an anatomically similar environment. The training tools in this study were based on widely used and validated assessments, [12, 13] and our inter-observer reliability for the suturing assessments was very high. The two assessors were not involved with the individual participants' training, and they were blinded to the trainee's specialty, experience and to each other's scores.

This study was localised to the North-West region of the UK and testing it on a national level would provide more precision around the estimates of skill and enhance external validity.

The effect of training grade was only apparent in the suturing and knot tying exercise. The effect of training grade was only apparent in the suturing and knot tying exercise. In the original study validating LASTT model, the novices were predominantly students with little or no operative experience and the experts were specialists with significant experience in advance surgical procedures. So, although the original study showed significant differences between novices and experts,[12] our junior group was more experienced than their novices. Therefore, it is possible that such differences were not large enough between our groups.

Simulation practice can facilitate the acquisition of new surgical skills if used systemically and comprehensively. Only a minority of the trainees undertook regular simulation and as such it is unlikely to have had a significant effect on the study tasks. Nonetheless, the type of simulation practice in this study has not been recorded, and this is a limitation.

The sample size may appear small for an observational study. Nonetheless, there are no previous studies available examining a similar aspect, and due to the difficulties in estimating the minimum difference considered important in this context, a priori sample size estimation was not possible. Consequently, along with the mean and SD values, we also included CIs and effect sizes to enable future meta-analysis as well as inform readers of the precision and magnitude of the results.

Finally, the male: female ratio between the specialty groups was considerably different, probably reflecting the relevant population in each specialty. Although evidence points to lack of differences between male and female surgeons,[40, 41] future studies should aim to equate the participants based on sex, to alleviate any concerns around grouping male and female surgeons together.

Unanswered questions and future research

The validity of evaluating core psychomotor skills in laparoscopic surgery needs to be assessed against actual performance in the operating theatre. Our work showed that trainees with limited experience found

For peer review only

suturing (an actual surgical procedure) challenging but not the core psychomotor tasks. This implies that it is not just the mastery of core skills, but the cognitive and motor processes involved in applying these skills which may influence performance on actual surgical procedures. Therefore, future studies could look at cognitive and musculoskeletal stress amongst the two specialities and the seniority of its trainees.

Acknowledgements: We would like to thank Karl Storz, Medtronic, BSGE and BRIDGES society for their financial support with the study. We would also like to thank Mr Brice Rodriguez, Mr Kenn Ma, Dr Alison Sambrook, Mr Kenn Lim, Mr Mohamed Elsherbiny, Mr Mohamed Abdelrahman, Ms Christa Hammill, Ms Aleyamma Abraham, and Dr Nicholas Evans for their help with study protocol and conducting this study.

Funding: This study was funded by grants from the British Society of Gynaecological Endoscopy, Karl Storz and Medtronic.

Conflict of interest: None declared.

Author contributions: DAS and ZK conceived and developed the research idea. ZK, DS, DAS, CG, TJC and EH designed and implemented the study protocol. ZK, DS, AS, JEL, TJC, EH, KA, TB, CG and DAS conducted the study. TB, CG and ZK analysed the data. ZK, DS, AS, TJC, TB, CG, and DAS prepared the manuscript. All authors reviewed and approved the final manuscript.

Data statement: Data are available from the corresponding author (CG) upon reasonable request.

Ethics approval statement: Ethical approval was granted by the Faculty of Health and Medicine Research Ethics Committee at Lancaster University (FHMREC20033)

REFERENCES

1. Harrell AG, Heniford BT. Minimally invasive abdominal surgery: lux et veritas past, present, and future. The American Journal of Surgery. 2005;190(2):239-43.
2. Hasson H, Rotman C, Rana N, Kumari NA. Open Laparoscopy: 29- Years Experience. Obstet Gynecol. 2000;96:763-6.
3. Royal College of Obstetricians and Gynaecologists. Royal College of Obstetricians and Gynaecologists, Core Curriculum for Obstetrics and Gynaecology: RCOG; 2019 [updated June 2020. Available from: https://www.gmc-uk.org/-/media/documents/core-og-curriculum-2019-final-gmc-approved-20210504_pdf-79992893.pdf.
4. Intercollegiate surgical curriculum program (ISCP). Intercollegiate surgical curriculum program, Core Surgical Training Curriculum: ISCP; 2021 [updated Aug 2021. Available from: https://www.iscp.ac.uk/media/1326/core-surgical-training-curriculum-2021-minor-changes-for-august-2022.pdf.
5. Molinas CR, Campo R. Defining a structured training program for acquiring basic and advanced laparoscopic psychomotor skills in a simulator. Gynecological Surgery. 2010;7(4):427-35.
6. Molinas CR, Campo R. Retention of laparoscopic psychomotor skills after a structured training program depends on the quality of the training and on the complexity of the task. Gynecol Surg. 2016;13(4):395-402.
7. Royal College of Obstetricians and Gynaecologists. Royal College of Obstetricians and Gynaecologists, Training matrix London: RCOG; 2022 [Available from: https://www.rcog.org.uk/careers-and-training/starting-your-og-career/specialty-training/assessment-and-progression-through-training/training-matrix/.

For peer review only

8. Royal College of Obstetricians and Gynaecologists. Royal College of Obstetricians and Gynaecologists, Advanced Training in Obstetrics & Gynaecology: RCOG; 2019 [updated Aug 2019. Available from: https://www.rcog.org.uk/media/xigldqq1/advanced-training-definitive-document-2019.pdf.
9. McMurray R, Lawrence T, Afors K. Minimal access in gynaecological surgery: training the minimally invasive gynaecological surgeon. Obstetrics, Gynaecology & Reproductive Medicine. 2022;32(7):135-40.
10. Royal College of Obstetricians and Gynaecologists. Royal College of Obstetricians and Gynaecologists, Training Evaluation Feedback on Gynaecology training London: RCOG; 2021 [updated c2021. Available from: https://www.rcog.org.uk/media/vrkcsb4b/tef-2021-report-gynaecology-training.pdf.
11. Thomas C, Griffiths G, Abdelrahman T, Santos C, Lewis W. Does UK surgical training provide enough experience to meet today's training requirements? Bmj [Internet]. 2015 [cited 2022 October]; 350(h2503). Available from: https://www.bmj.com/content/350/bmj.h2503.
12. Molinas CR, De Win G, Ritter O, Keckstein J, Miserez M, Campo R. Feasibility and construct validity of a novel laparoscopic skills testing and training model. Gynecological surgery. 2008;5(4):281-90. eng.
13. Campo R, Reising C, Van Belle Y, Nassif J, O'Donovan P, Molinas CR. A valid model for testing and training laparoscopic psychomotor skills. Gynecological surgery. 2010;7(2):133-41. eng.
14. Campo R, Wattiez A, Leon De Wilde R, Molinas Sanabria CR. Training in laparoscopic surgery: From the lab to the or. Slovenian Journal of Public Health. 2012;51(4):285-98.
15. European Academy of Gynaecological Surgery. European Academy of Gynaecological Surgery, Laparoscopic Skills Training and Testing Method Europe: MiS Academy Europe; 2022 [Available from: https://europeanacademy.org/training-tools/lastt/.
16. Sleiman Z, Tanos V, Van Belle Y, Carvalho JL, Campo R. European Academy laparoscopic "Suturing Training and Testing'' significantly improves surgeons' performance. Facts Views Vis Obgyn. 2015;7(3):153-60. eng.
17. Feldman LS, Cao J, Andalib A, Fraser S, Fried GM. A method to characterize the learning curve for performance of a fundamental laparoscopic simulator task: defining "learning plateau" and "learning rate". Surgery. 2009;146(2):381-6.
18. LapPass Academy [online video]. Journal of surgical simulation. Laparoscopic Intracorporeal Suturing and Knot Tying, task 4. 1 video: 130 sec, HD, colour. UK: Journal of surgical simulation; 2020.
19. Field AP, Wilcox RR. Robust statistical methods: A primer for clinical psychology and experimental psychopathology researchers. Behav Res Ther. 2017;98:19-38.
20. Daszykowski M, Kaczmarek K, Vander Heyden Y, Walczak B. Robust statistics in data analysis — A review. Chemometrics and Intelligent Laboratory Systems. 2007;85(2):203-19.
21. Wilcox RR, Tian TS. Measuring effect size: a robust heteroscedastic approach for two or more groups. Journal of Applied Statistics. 2011;38(7):1359-68.
22. Brydges CR. Effect Size Guidelines, Sample Size Calculations, and Statistical Power in Gerontology. Innov Aging. 2019;3(4):1-8.
23. Tavakol M, Dennick R. Making sense of Cronbach's alpha. Int J Med Educ. 2011 Jun 27;2:53-5.
24. Bland JM, Altman DG. Statistics notes: Cronbach's alpha. BMJ [Internet]. 1997; 314(572).
25. Sadideen H, Alvand A, Saadeddin M, Kneebone R. Surgical experts: born or made? Int J Surg. 2013;11(9):773-8.
26. Reznick RK, MacRae H. Teaching Surgical Skills — Changes in the Wind. N Engl J Med. 2006;355(25):2664-9. eng.
27. Dawe SR, Pena GN, Windsor JA, Broeders JAJL, Cregan PC, Hewett PJ, et al. Systematic review of skills transfer after surgical simulation-based training. British Journal Of Surgery. 2014;101(9):pp1063-76. eng.
28. Train AT, Hu J, Narvaez JRF, Towle-Miller LM, Wilding GE, Cavuoto L, et al. Teaching surgery novices and trainees advanced laparoscopic suturing: a trial and tribulations. Surgical endoscopy. 2020;35(10):5816-26. eng.
29. Odejinmi F, Rizzuto I, Ballard KD. Potential barriers to the laparoscopic management of ectopic pregnancies: a regional UK study. Acta Obstet Gynecol Scand. 2010;89(10):1350-3. eng.
30. Cecilio-Fernandes D, Cnossen F, Jaarsma D, Tio RA. Avoiding Surgical Skill Decay: A Systematic Review on the Spacing of Training Sessions. J Surg Educ. 2018;75(2):471-80.
31. Gallagher AG, Jordan-Black JA, O'Sullivan GC. Prospective, randomized assessment of the acquisition, maintenance, and loss of laparoscopic skills. Ann Surg. 2012;256(2):387-93.
32. Moss EL, Bredaki FE, Jones PW, Hollingworth J, Luesley DM, Chan KK. Is gynaecological surgical training a cause for concern? A questionnaire survey of trainees and trainers. BMC Med Educ. 2011;11(1):32-. eng.

For peer review only

33. Christopoulos G, Kelly T, Lavery S, Trew G. Surgical skills of specialty trainees in emergency gynaecological laparoscopic procedures: a national UK survey. J Obstet Gynaecol. 2014;34(5):435-8.
34. Issenberg SB, McGaghie WC, Petrusa ER, Lee Gordon D, Scalese RJ. Features and uses of high-fidelity medical simulations that lead to effective learning: a BEME systematic review. Med Teach. 2005;27(1):10-28.
35. Gala R, Orejuela F, Gerten K, Lockrow E, Kilpatrick C, Chohan L, et al. Effect of validated skills simulation on operating room performance in obstetrics and gynecology residents: a randomized controlled trial. Obstet Gynecol. 2013;121(3):578-84.
36. Palter VN, Grantcharov TP. Development and validation of a comprehensive curriculum to teach an advanced minimally invasive procedure: a randomized controlled trial. Ann Surg. 2012;256(1):25-32.
37. Aggarwal R, Ward J, Balasundaram I, al. e. Proving the effectiveness of virtual reality simulation for training in laparoscopic surgery. Ann Surg. 2007;246(5):771-9.
38. Bryant-Smith A, Rymer J, Holland T, Brincat M. 'Perfect practice makes perfect': the role of laparoscopic simulation training in modern gynaecological training. The obstetrician & gynaecologist. 2020;22(1):69-74. eng.
39. American College of Obstetricians & Gynaecologists. American College of Obstetricians & Gynaecologists Washington: ACOG; 2022 [updated c2022. Available from: https://www.acog.org/education-and-events/creog/curriculum-resources/additional-curricular-resources/fundamentals-of-laparoscopic-surgery.
40. Ali A, Subhi Y, Ringsted C, Konge L. Gender differences in the acquisition of surgical skills: a systematic review. Surg Endosc. 2015;29(11):3065-73. eng.
41. Busshoff J, Datta RR, Bruns T, Kleinert R, Morgenstern B, Pfister D, et al. Gender benefit in laparoscopic surgical performance using a 3D-display system: data from a randomized cross-over trial. Surg Endosc. 2022 Jun;36(6):4376-85. PubMed PMID: 34750707. PMCID: PMC9085658. Epub 20211108.

Figure legends

Figure 1 - Outline of the training pathways in GS (a) and O&G (b). Adapted from the Royal College of Obstetricians & Gynaecologists (RCOG) (3) and intercollegiate surgical curriculum program (ISCP). (4)

Figure 2 - Time taken to complete laparoscopic tasks (a-c) and laparoscopic precision of movements by speciality (d-f). Task completion time for LCN (a), HEC (b) and BMC (c). Trainees' ability to integrate camera navigation skills with speed (d), the number of drops in HEC (e) and the number of drops in BMC (f). Data are presented as mean ± SD.

Figure 3 - Total suturing scores by trainee's experience within O&G and GS. Data are presented as mean ± SD.

For peer review only

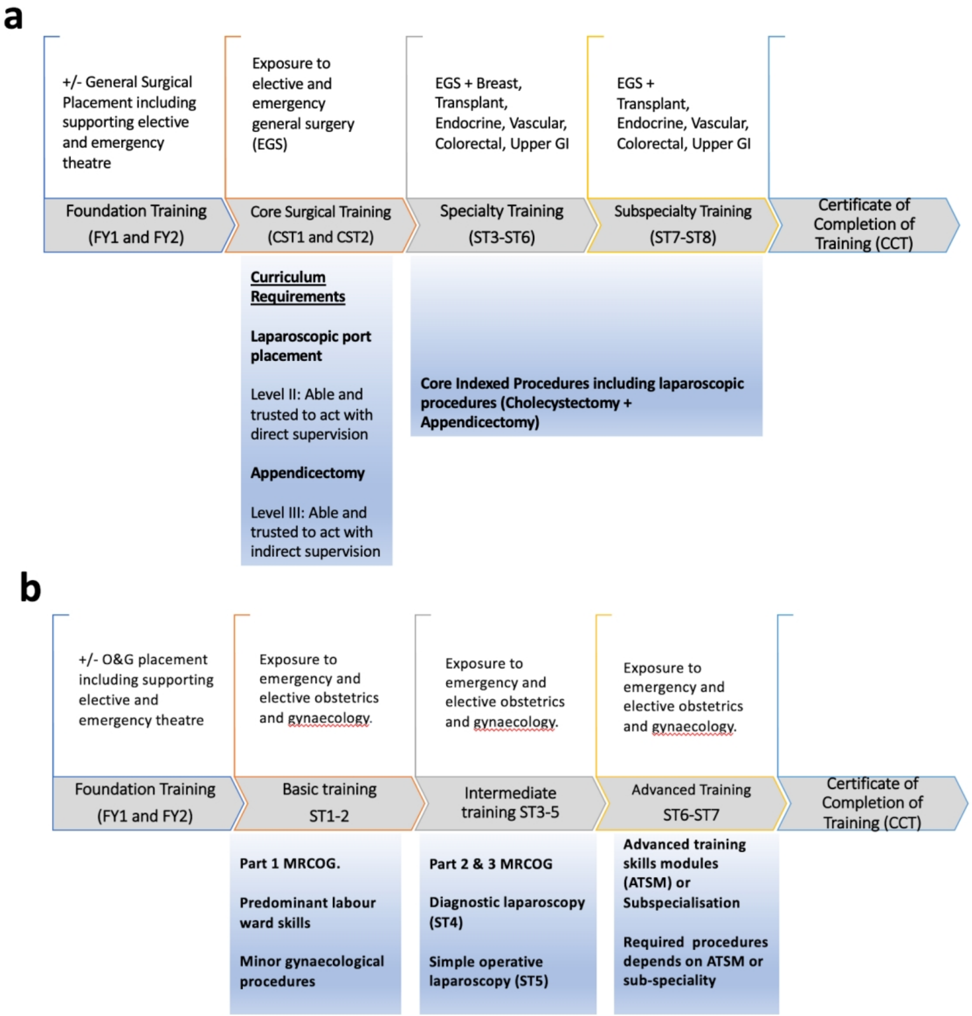

Figure 1: Outline of the training pathways in GS (a) and O&G (b). Adapted from the Royal College of Obstetricians & Gynaecologists (RCOG) (3) and intercollegiate surgical curriculum program (ISCP). (4)

140x145mm (300 x 300 DPI)

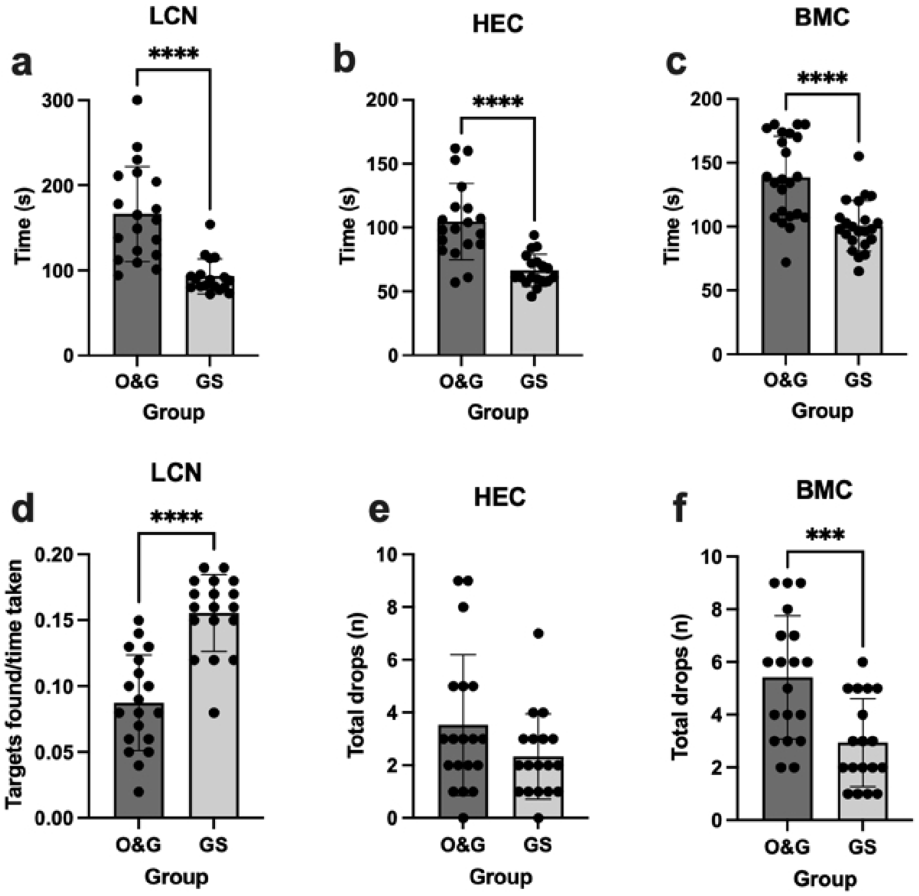

Figure 2. Time taken to complete laparoscopic tasks (a-c) and laparoscopic precision of movements by speciality (d-f). Task completion time for LCN (a), HEC (b) and BMC (c). Trainees' ability to integrate camera navigation skills with speed (d), the number of drops in HEC (e) and the number of drops in BMC (f). Data are presented as mean ± SD.

175x164mm (100 x 100 DPI)

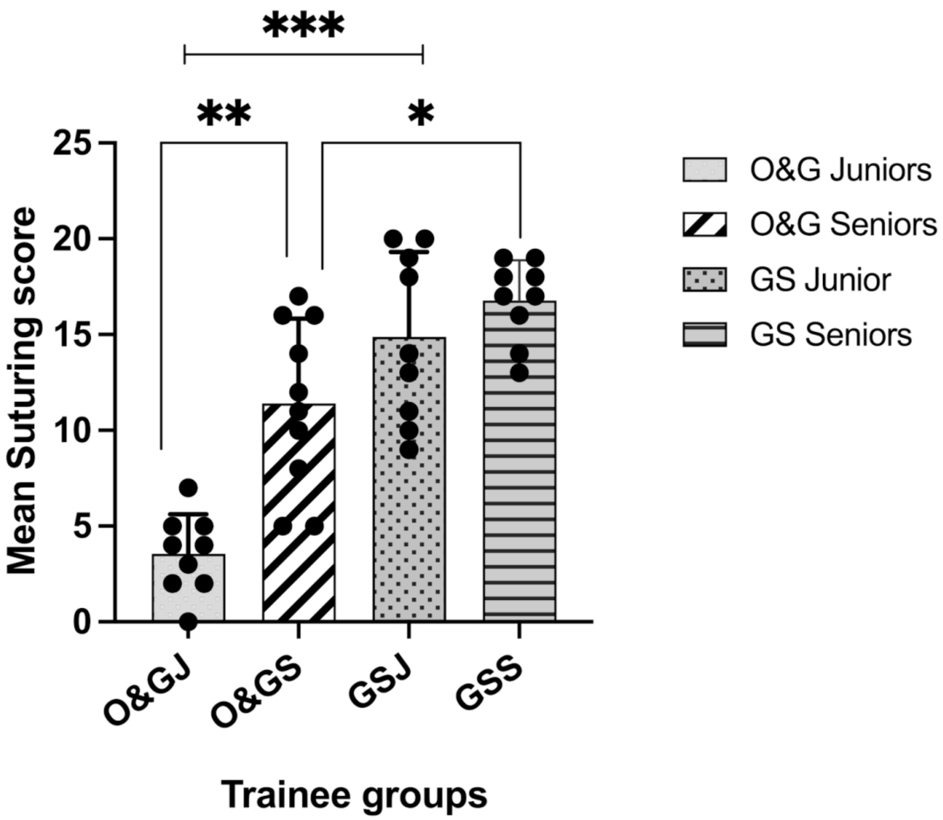

Figure 3. Total suturing scores by trainee’s experience within O&G and GS. Data are presented as mean ± SD.

128x108mm (300 x 300 DPI)

Table S1: Training matrix for O&G (adapted from RCOG). Required laparoscopic competencies are highlighted in **bold**. Competencies are signed off based on an entrustability scale* and as such no indicative numbers are included here.

	ST1	ST2	ST3	ST4	ST5	ST6	ST7
Curriculum	CiP progress appropriate to the relevant stage as per the CiP guides and entrustability levels.						
Examination	MRCOG Part 1			MRCOG Part 2 & Part 3			
At least 3 summative OSATS confirming competency by more than one assessor. At least one OSAT confirming competence should be supervised by a consultant.	Cervical smear	Caesarean section (Basic)<br><br>Non rotational assisted vaginal delivery (Ventouse & forceps)<br><br>Perineal repair<br><br>Surgical management of miscarriage/Surgical termination of pregnancy<br><br>Insertion of an Intrauterine system/intrauterine contraceptive device.<br><br>Endometrial biopsy	Manual removal of placenta<br><br>Transabdominal USS of early and late pregnancy	Hysteroscopy<br><br>**Diagnostic laparoscopy**<br><br>3rd degree perineal repair<br><br>Vulval biopsy	**Simple operative laparoscopy** (laparoscopic sterilisation or simple adnexal surgery e.g. adhesiolysis/ ovarian drilling<br><br>Caesarean section (Intermediate)<br><br>Rotational assisted vaginal delivery (any method)		Subspecialty specific competencies.<br><br>**Laparoscopic management of ectopic pregnancy**<br><br>**Ovarian cystectomy (laparoscopic & open)**<br><br>Surgical management of post partum haemorrhage

*Entrustability scale: 1= observe only, 2= direct supervision, 3= indirect supervision, 4= act independently with support, 5= act independently.

For peer review only

Table S2: Summary of required procedures in GS training. Indicative case/operative numbers are given for the specialty training phase where both the numbers and entrustability scales are used for assessment.

	CT1 Phase 1	CT2 Phase 1	ST3 Phase 2	ST4 Phase 2	ST5 Phase 2	ST6 Phase 2	ST7 Phase 3	ST8 Phase 3
Examinations		MRCS Part A<br>MRCS Part B						FRCS Part 1<br>FRCS Part 2
**Operative Requirements**<br>Level 1<br>Has observed<br>Level 2<br>Can do with assistance<br>Level 3<br>Can do whole but may need assistance<br>Level 4<br>Competent to do without assistance, including complications		Induction of pneumoperitoneum for laparoscopy with port placement (Level 2)<br>Appendicectomy (Level 3)<br>Open and close midline laparotomy incision (2)<br>Inguinal hernia repair (Level 2)<br>Primary abdominal wall hernia repair (Level 2)				Inguinal Hernia (level 4) [50 cases*]<br>Cholecystectomy (level 3) [40 cases*]<br>Segmental Colectomy (level 3) [15 cases*]<br>Emergency Laparotomy [45* cases]<br>Appendicectomy [60 cases*]		Emergency Laparotomy (Level 4) [100 cases*]<br>Appendicectomy (Level 4) [80 cases*]<br>Cholecystectomy [50 cases*] (level 4)<br>Segmental colectomy [20 cases*] (level 4)
**Other Operative Technical Skills**		Chest drain insertion (Level 3)<br>Needle biopsy including fine needle aspiration (Level 3)<br>Rigid sigmoidoscopy (Level 3)<br>Excision biopsy of benign skin or subcutaneous lesion (Level 4)						Indicative numbers and competencies for chosen specialty required. (Hepatopancreaticobiliary, Transplant, Endocrine, Colorectal, Oesophagogastric)

For peer review only

Reporting checklist for cross sectional study.

Based on the STROBE cross sectional guidelines.

Instructions to authors

Complete this checklist by entering the page numbers from your manuscript where readers will find each of the items listed below.

Your article may not currently address all the items on the checklist. Please modify your text to include the missing information. If you are certain that an item does not apply, please write "n/a" and provide a short explanation.

Upload your completed checklist as an extra file when you submit to a journal.

In your methods section, say that you used the STROBE cross sectionalreporting guidelines, and cite them as:

von Elm E, Altman DG, Egger M, Pocock SJ, Gotzsche PC, Vandenbroucke JP. The Strengthening the Reporting of Observational Studies in Epidemiology (STROBE) Statement: guidelines for reporting observational studies.

	Reporting Item	Page Number
**Title and abstract**		
Title	Indicate the study's design with a commonly used term in the title or the abstract	1
Abstract	Provide in the abstract an informative and balanced summary of what was done and what was found	2
**Introduction**		
Background / rationale	Explain the scientific background and rationale for the investigation being reported	3
Objectives	State specific objectives, including any prespecified hypotheses	3
**Methods**		
Study design	Present key elements of study design early in the paper	4
Setting	Describe the setting, locations, and relevant dates, including periods of recruitment, exposure, follow-up, and data collection	4

For peer review only

Eligibility criteria	Give the eligibility criteria, and the sources and methods of selection of participants.	4
	Clearly define all outcomes, exposures, predictors, potential confounders, and effect modifiers. Give diagnostic criteria, if applicable	5-6
Data sources / measurement	For each variable of interest give sources of data and details of methods of assessment (measurement). Describe comparability of assessment methods if there is more than one group. Give information separately for for exposed and unexposed groups if applicable.	5-6
Bias	Describe any efforts to address potential sources of bias	5-6
Study size	Explain how the study size was arrived at	Sample size not possible to calculate as no previous study on this topic to base clinically significant effect size, and standard deviation measures on.
Quantitative variables	Explain how quantitative variables were handled in the analyses. If applicable, describe which groupings were chosen, and why	6
Statistical methods	Describe all statistical methods, including those used to control for confounding	7
Statistical methods	Describe any methods used to examine subgroups and interactions	7
Statistical methods	Explain how missing data were addressed	7
Statistical methods	If applicable, describe analytical methods taking account of sampling strategy	NA
Statistical methods	Describe any sensitivity analyses	NA

Results

For peer review only

Participants	Report numbers of individuals at each stage of study—eg numbers potentially eligible, examined for eligibility, confirmed eligible, included in the study, completing follow-up, and analysed. Give information separately for for exposed and unexposed groups if applicable.	7
Participants	Give reasons for non-participation at each stage	7
Participants	Consider use of a flow diagram	NA
Descriptive data	Give characteristics of study participants (eg demographic, clinical, social) and information on exposures and potential confounders. Give information separately for exposed and unexposed groups if applicable.	8
Descriptive data	Indicate number of participants with missing data for each variable of interest	7-8
Outcome data	Report numbers of outcome events or summary measures. Give information separately for exposed and unexposed groups if applicable.	8-10
Main results	Give unadjusted estimates and, if applicable, confounder-adjusted estimates and their precision (eg, 95% confidence interval). Make clear which confounders were adjusted for and why they were included	7-10
Main results	Report category boundaries when continuous variables were categorized	9-10
Main results	If relevant, consider translating estimates of relative risk into absolute risk for a meaningful time period	No risks/RR reported
Other analyses	Report other analyses done—e.g., analyses of subgroups and interactions, and sensitivity analyses	9-10
**Discussion**		
Key results	Summarise key results with reference to study objectives	11
Limitations	Discuss limitations of the study, taking into account sources of potential bias or imprecision. Discuss both direction and magnitude of any potential bias.	12

For peer review only

Interpretation	Give a cautious overall interpretation considering objectives, limitations, multiplicity of analyses, results from similar studies, and other relevant evidence.	11-12
Generalisability	Discuss the generalisability (external validity) of the study results	12
**Other Information**		
Funding	Give the source of funding and the role of the funders for the present study and, if applicable, for the original study on which the present article is based	13

None The STROBE checklist is distributed under the terms of the Creative Commons Attribution License CC-BY. This checklist can be completed online using https://www.goodreports.org/, a tool made by the EQUATOR Network in collaboration with Penelope.ai

For peer review only

BMJ Open

A prospective observational study comparing proficiency of obstetrics & gynaecology trainees with general surgical trainees using simulated laparoscopic tasks in Health Education England, North-West

Journal:	*BMJ Open*
Manuscript ID	bmjopen-2023-075113.R3
Article Type:	Original research
Date Submitted by the Author:	11-Oct-2023
Complete List of Authors:	Khan, Zaibun N; Royal Lancaster Infirmary<br>Shrestha, Donna; Lancaster University<br>Shugaba, Abdulwarith; Lancaster University<br>Lambert, Joel E; Lancaster University<br>Clark, T; Birmingham Women's NHS Foundation Trust, Gynaecology; University of Birmingham,<br>Haslett, Elizabeth; Blackpool Victoria Hospital<br>Afors, Karolina; Whittington Health NHS Trust<br>Bampouras, Theodoros M; School of Sport and Exercise Sciences<br>Gaffney, Christopher; Lancaster University<br>Subar, Daren; East Lancashire Hospitals NHS Trust
**Primary Subject Heading**:	Surgery
Secondary Subject Heading:	Obstetrics and gynaecology, Medical education and training
Keywords:	OBSTETRICS, GYNAECOLOGY, MEDICAL EDUCATION & TRAINING

SCHOLARONE™
Manuscripts

I, the Submitting Author has the right to grant and does grant on behalf of all authors of the Work (as defined in the below author licence), an exclusive licence and/or a non-exclusive licence for contributions from authors who are: i) UK Crown employees; ii) where BMJ has agreed a CC-BY licence shall apply, and/or iii) in accordance with the terms applicable for US Federal Government officers or employees acting as part of their official duties; on a worldwide, perpetual, irrevocable, royalty-free basis to BMJ Publishing Group Ltd ("BMJ") its licensees and where the relevant Journal is co-owned by BMJ to the co-owners of the Journal, to publish the Work in this journal and any other BMJ products and to exploit all rights, as set out in our licence.

The Submitting Author accepts and understands that any supply made under these terms is made by BMJ to the Submitting Author unless you are acting as an employee on behalf of your employer or a postgraduate student of an affiliated institution which is paying any applicable article publishing charge ("APC") for Open Access articles. Where the Submitting Author wishes to make the Work available on an Open Access basis (and intends to pay the relevant APC), the terms of reuse of such Open Access shall be governed by a Creative Commons licence – details of these licences and which Creative Commons licence will apply to this Work are set out in our licence referred to above.

Other than as permitted in any relevant BMJ Author's Self Archiving Policies, I confirm this Work has not been accepted for publication elsewhere, is not being considered for publication elsewhere and does not duplicate material already published. I confirm all authors consent to publication of this Work and authorise the granting of this licence.

For peer review only

A prospective observational study comparing proficiency of obstetrics & gynaecology trainees with general surgical trainees using simulated laparoscopic tasks in Health Education England, North-West

Zaibun N Khan[1] (0000-0002-9558-8964), Donna Shrestha [2] (0000-0001-8711-3515), Abdulwarith Shugaba [2], Joel E Lambert [2] (0000-0001-5663-6284), T Justin Clark [3] (0000-0002-5943-1062), Elizabeth Haslett [4] (0000-0003-1165-0415), Karolina Afors [5], Theodoros M Bampouras [6] (0000-0002-8991-4655), Christopher J Gaffney [2]* (0000-0001-7990-2792), Daren A Subar [7].

Affiliations:

1. Royal Lancaster Infirmary, Ashton Rd, Lancaster, LA1 4RP, UK.

2. Lancaster Medical School, Lancaster University, Health Innovation One, Sir John Fisher Drive, Lancaster, LA1 4AT, UK.

3. Birmingham Women's Hospital, Birmingham Women's & Children's NHS Foundation Trust, Metchley Park Road, Edgbaston, Birmingham, B15 2TG, UK.

4. Blackpool Victoria Hospital, Whinney Heys Road, Blackpool, FY3 8NR.

5. Whittington Health NHS Trust, Magdala Avenue, London N19 5NF, UK.

6. Liverpool John Moores University, School of Sport and Exercise Sciences, Tom Reilly Building, Byrom Street, Liverpool, L3 3AF, UK.

7. East Lancashire Hospitals NHS Trust, Haslingden Road, Blackburn, BB2 3HH, UK.

Correspondence to: Dr Christopher J Gaffney, Lancaster Medical School, Lancaster University, Health Innovation One, Sir John Fisher Drive, Lancaster, LA1 4AT, UK; c.gaffney@lancaster.ac.uk; +44 (0) 1524 593 602

Word count: 3984

For peer review only

ABSTRACT

BACKGROUND: Training programmes for obstetrics and gynaecology (O&G) and general surgery (GS) vary significantly, but both require proficiency in laparoscopic skills. We sought to determine performance in each specialty.

DESIGN: Prospective, Observational study.

SETTING: Health Education England North-West, UK.

PARTICIPANTS: 47 surgical trainees (24 O&G and 23 GS) were sub-divided into four groups: 11 junior O&G, 13 senior O&G, 11 junior GS, and 12 senior GS trainees.

OBJECTIVES: Trainees were tested on four simulated laparoscopic tasks; laparoscopic camera navigation (LCN), hand eye co-ordination (HEC), bimanual co-ordination (BMC) and suturing with intracorporeal knot tying (suturing).

RESULTS: O&G trainees completed LCN (P <0.001), HEC (P <0.001) and BMC (P <0.001) significantly slower than GS trainees. Furthermore, O&G found fewer number of targets in LCN (P =0.001) and dropped a greater number of pins than the GS trainees in BMC (P =0.04). In all three tasks, there were significant differences between O&G and GS trainees but no difference between the juniors and senior groups within each specialty. Performance in suturing also varied by specialty; senior O&G trainees scored significantly lower than senior GS trainees; O&G 11.4 ± 4.4 vs GS 16.8 ± 2.1, P = 0.03. Whilst suturing scores improved with seniority among O&G trainees, there was no difference between the junior and senior GS trainees; senior O&G 11.4 ± 4.4 vs junior O&G 3.6 ± 2.1, P = 0.004.

DISCUSSION: GS trainees performed better than O&G trainees in core laparoscopic skills and the structure of obstetrics and gynaecology training may require modification.

Keywords: Laparoscopy; obstetrics; gynaecology; surgical training

For peer review only

Strengths and Limitations

- The study's prospective design, robust data collection techniques including duplicate and blinded outcome assessment, and use of validated tools allowed us to minimise bias.
- The study reported effect sizes as well standard deviations and confidence intervals to allow the reader to assess the magnitude of study findings.
- The generalisability of the study can be enhanced if the study is repeated on a national or international scale.
- Larger comparative cohorts can provide more precision around the estimates of skill and allow adjustment for potential prognostic factors.

For peer review only

INTRODUCTION

The foundations of laparoscopic surgery were laid by gynaecologists and the first sterilisation procedure was performed laparoscopically in 1936.[1] Gynaecologists have led advancements in laparoscopy through innovation in laparoscopic instruments and educational tools such as the pelvic simulator trainer and Hasson's open technique for entry, which is widely used by general surgeons today. [1, 2]

Obstetrics and gynaecology (O&G) and general surgery (GS) trainees are required to demonstrate competency in different procedures, [3, 4] however, the core psychomotor skills required for laparoscopy are similar. Some of these skills include laparoscopic camera navigation (LCN), hand eye co-ordination (HEC) and bimanual co-ordination (BMC). Surgical trainees should be proficient in these skills early in their training to enable development of more complex and specific laparoscopic procedural techniques. [5, 6]

O&G training, lasting seven years, consists of basic (ST1-ST2), intermediate (ST3-ST5) and advanced training (ST6-ST7). The training covers both obstetrics and gynaecology although there is a significant focus on acquiring obstetric competencies throughout the training. [7] Exposure to laparoscopic surgery is gained only through gynaecological practice. Trainees who wish to pursue gynaecological training can select Advanced Training Skills Modules (ATSM) or subspecialisation relevant to gynaecological surgery in the advance part of the program. [8] In contrast, GS training is eight years long, including two years of core surgical training (CST1-2) and six years of higher surgical training (ST3-ST8), where the final two years focusses on subspecialty training (Figure 1). [4] GS trainees are required to be independent in laparoscopic appendicectomy by the end of CST2. [4] In contrast, O&G trainees are expected to perform 'minor operative laparoscopy' by the end of the fifth training year. [3] GS trainees, therefore, gain laparoscopic experience throughout their training programme whilst O&G trainees receive most of their laparoscopic surgery exposure in the advanced part of the program. [8, 9] The content of each stage of laparoscopic training in obstetrics and gynaecology and general surgery training is detailed in Table S1 and S2.

Opportunities for theatre experience appear to be lacking in both specialties. In 2021, the Royal College of Obstetricians and Gynaecologists (RCOG) evaluated the training of 1415 trainees and found that less than half of the ST5 and ST6 trainees reported adequate opportunities to develop the required surgical skills relevant to their stage of training.[10] Similarly, amongst 155 GS applicants certifying for completion of training, only two-thirds had reached the required number of cases. However, nearly three-quarters of these trainees had met the requirements for key procedures in their field. [11]

Our study compared the proficiency in core laparoscopic psychomotor skills amongst junior (ST3-ST5 in both specialties) and senior trainees (ST6-ST7 in O&G ST6-ST8 in GS) using a Karl Storz Szabo-Berci box trainer. We hypothesised that there is no difference in the performance of core laparoscopic skills between O&G and GS trainees at all training stages.

For peer review only

METHODS

Participants

47 trainees (24 O&G and 23 GS) from Health Education England North-West (HEENW) were invited to participate in this prospective observational study between September 2021 and April 2022. Trainees were allocated a study number, which was recognisable only to the two study investigators involved in the recruitment of trainees. To explore the effect of surgical experience, the trainees were sub-divided by their training grades into four groups: junior O&G, senior O&G, junior GS and senior GS.

The 'junior' group consisted of trainees between ST3 and ST5, and the 'senior' group included trainees in the final two years of O&G and GS training programs. For the senior O&G group, we selected trainees undertaking one of the advanced modules in 'advanced laparoscopy for the excision of benign disease', 'benign abdominal surgery - open and laparoscopic' and 'gynae-oncology'. This was to enable the selection of trainees in receipt of regular gynaecology theatre sessions and, therefore, comparable with GS seniors. Senior GS trainees with a specialist interest in breast surgery were excluded due to limited laparoscopic work within this subspecialty.

All participants provided written informed consent prior to participation. They completed a questionnaire collecting data on demographic details and factors relating to laparoscopic proficiency, such as the use of video games and laparoscopic simulators, attendance at courses involving laparoscopic surgery, training stage at first exposure to laparoscopic work, and the typical frequency of attendance in theatre.

The study was approved by the O&G and GS heads of schools from HEENW. Ethical approval was granted by the Faculty of Health and Medicine Research Ethics Committee at Lancaster University (FHMREC20033) and testing was conducted in accordance with the Declaration of Helsinki. Following ethical approval, the study was registered at clinicaltrials.gov (NCT05116332).

Patient and public involvement

No patient involved.

Procedures

All trainees were assessed by two faculty members/assessors in individual rooms to minimise external distractions. Assessors were not involved in the training of any study participants and trainees were able to discretely request a different assessor (s) if they knew the pre-assigned member or felt uncomfortable with them, without giving a reason. Trainee's specialty and training stage was concealed from the assessors to ensure anonymity of trainees and blinding of the assessors. Laparoscopic proficiency was measured by observing four standardised, simulated tasks using validated assessment tools. [5, 12-14] All trainees received the same written and video instructions explaining the task before beginning any assessments.[15] All tasks were performed on a Karl Storz Szabo-Berci-Sackier laparoscopic trainer. The first three tasks assessed core laparoscopic psychomotor skills using the Laparoscopic Skills Training and Testing (LASTT) model. [13] The fourth task evaluated laparoscopic suturing and was assessed using the suturing and knot tying training and testing (SUTT-1) method by the European Academy of Gynaecological Surgery. [16] Trainees performed each task three times, except for the suturing task, which was completed once. The rationale behind restricting repetition to three iterations was to familiarise trainees to the task so that their optimal performance could be elicited without inducing a significant rehearsal effect. [17]

For peer review only

The same equipment was used throughout the testing period for all trainees. All assessors received standardised training modified from the "Training the Trainers" of the Gynaecological Endoscopic Surgical Education and Assessment (GESEA) program. This consisted of an overview of all study tasks, instruments, scoring systems, and specifics details relating to set up and delivery of all the study tasks. Tasks were performed in order of increasing technical difficulty as described below.

Tasks

Task 1 - Laparoscopic Camera Navigation (LCN)

This task assessed the trainees' ability to navigate a 30° 10mm laparoscope to find 14 targets within the LASTT model. [12-14] The maximum time allowed was 300 seconds per iteration. A validation study on the LASTT model showed that the median time for task completion was 188 seconds for novices and ranged between 142 and 292 seconds. [12] O&G trainees use 30°telescope in hysteroscopic surgery and when using smaller laparoscopes. As the experience with using larger 30° laparoscopes may have been limited, we used the upper limit of the time range as the allocated time.

On the scoring sheet, the time taken to identify all 14 targets, or the last target identified within 300 seconds, was recorded. The task was considered successful when all 14 targets were identified in every iteration within the allocated timeframe. The trainees' best time (of the three iterations) was used to assess the speed of task completion. To assess the trainees' ability to integrate speed with navigation skills, the ratio of the total number of targets found to the total time taken to complete the task was calculated.

Task 2 – Hand eye co-ordination (HEC)

This task required the trainee to transfer six coloured cylinders to their respective coloured pins using a forceps in their dominant hand and navigating a 0° laparoscope with their non-dominant hand. [12-14] Time permitted for this task was 180 seconds per iteration. [12, 13]

Completion was determined when six cylinders were placed on their pins within the allocated time. The trainees' best time was used to calculate the speed of task completion. We recorded the total number of times a cylinder was dropped during each iteration. A sum of the three iterations gave a total number of drops. This was used as an indicator of precision of movement.

Task 3 – Bimanual Co-ordination (BMC)

This task assessed the trainees' ability to transfer six coloured pushpins between forceps in their dominant and non-dominant hands and place them in their coloured slots on the LASTT model. [12-14] The assessor navigated the camera for the trainees based on their instructions. A maximum of 180 seconds was allowed per iteration and outcome measures were the same as for HEC.

Task 4 – Laparoscopic suturing and intracorporeal knot placement (suturing)

A foam pad was used to assess suturing and knot placement using the SUTT-1 method. [16] All trainees were shown a 60-second video demonstration of laparoscopic suturing and intracorporeal knot tying to ensure that the instructions were standardised, and expectations were clearly understood. [18] Trainees were asked to place four interrupted sutures and perform four intracorporeal knots comprising of three throws. A maximum of 15 minutes was permitted for this task. The quality of suturing and knot-tying was assessed by two experienced consultants (one O&G and one GS consultant; both with over 10 years of experience in laparoscopic suturing) after completion of the task using a validated SUTT scoring system. [16] The assessors were blinded to the trainee and each other's score. All components of the total suturing score, such as extent of trauma, were scored after thorough inspection of the foam pads. The suturing task

was deemed complete if four horizontal sutures and four secure knots were secured within 15 minutes. The median number of sutures and knots inserted (out of four) and the total suturing scores were analysed.

A summary of the surgical tasks and their assessment are provided in Table 1.

Table 1. Summary of laparoscopic surgical tasks using a box trainer and methods

Task	Iterations	Time allocated	Data recorded	Outcome
**1 – Laparoscopic camera navigation (LCN)**	3	300 seconds	Time taken to find 14 targets<br>If exceeding 300 seconds, the last target found	Best time*<br>No of targets found
**2 – Hand eye co-ordination (HEC)**	3	180 seconds	Time taken<br>No. of objects placed<br>No. of drops	Best time*<br>Overall no. of drops**
**3 – Bimanual co-ordination (BMC)**	3	180 seconds	Time taken<br>No. of objects placed<br>No. of drops	Best time*<br>Overall no. of drops**
**4 – Suturing and intracorporeal knot placement (suturing)**	1	15 minutes	Time taken<br>Quality of sutures and knots	Median no. of sutures and knots<br>Total suturing scores

*shortest completion time out of three iterations.
**sum of dropped objects across the three iterations.

Statistical analysis

The Chi squared test (χ^2) was used to analyse demographic, training related variables between specialties (Table 2) and successful completion of all tasks. All continuous variables are reported as mean, standard deviation and 95% confidence intervals.

Normality was checked for tasks 1-3, including the LCN time and efficiency ratio, HEC time and precision score, and BMC time and precision score. As normality was only confirmed for BMC time, a robust ANOVA [19, 20] was used to compare the junior and senior trainee groups within the two specialties. The Holm-Bonferroni post hoc test was carried out to locate the difference and adjust for multiple comparisons when a significant result was observed. Where trainee's surgical experience did not have a significant effect, robust independent t-tests were used to compare differences between O&G and GS. Effect sizes (ξ) were calculated for all significant comparisons and 0.1 was considered small, 0.3 moderate and 0.5 large.[21] BMC time was analysed using ANOVA to compare junior and senior trainee groups within the two specialties and independent t-tests to assess differences between specialties. Holm-Bonferroni post hoc test was carried out to locate the difference and adjust for multiple comparisons when a significant result was observed.

For peer review only

In the suturing task, the number of sutures and knots were compared between the four groups using the Kruskal Wallis test, with Holm-Bonferroni correction for multiple pairwise comparisons. This data is reported as median and interquartile range. Hedges *g* was calculated for all significant comparisons with 0.2, 0.5 and 0.8 considered as small, moderate and large, respectively. [22] Agreement of total suturing scores between assessors was examined with Cronbach's alpha. [23] According to Bland and Altman, $\alpha = 0.95$ is desirable for clinical applications.[24] Total suturing scores were analysed using robust statistics as above. Statistical analysis was conducted in Jamovi Version 2.3.18.0 (The Jamovi project, https://www.jamovi.org) while collation and creation of figures was completed in GraphPad Prism v9 (GraphPad Software, San Diego, Calif., USA). Statistical significance was set at $P \le 0.05$ and the corrected values are presented.

RESULTS

Participant Characteristics

Two trainees were excluded from the analysis as they did not meet the inclusion criteria (one senior O&G trainee) and had incomplete data (one senior GS trainee). 23 O&G trainees (mean ± SD, age 34 ± 4 years) and 22 GS trainees (34 ± 5 years) were selected for data analysis. The OG group consisted of 11 junior and 12 senior trainees and GS group consisted of 11 junior and 11 senior trainees. Both groups were not significantly different except their gender. Most O&G trainees were female in contrast to GS, where the majority were male.

Factors relating to proficiency in laparoscopic skills

Pre-testing baseline questionnaires showed that a significantly larger number of O&G trainees used a simulator than GS trainees; O&G 16 (70%) vs. GS 7 (32%), P =0.01. However, the number of trainees using the simulator frequently, such as once a month, was similar between the two specialties: O&G 3 (13%) vs. GS 2 (9%), P=0.32. O&G trainees reported attending significantly fewer elective and emergency laparoscopic theatre sessions; O&G 64 (37%) and 23 (19%) vs. GS 110 (63%) and 100 (81%), P <0.001 for both comparisons. However, analysis by training grade showed that senior O&G and senior GS trainees attended a similar number of elective sessions; O&G 51 (80%) vs. GS 56 (51%), P=0.30. Furthermore, junior O&G trainees were assigned to an assistant's role significantly more frequently than junior GS trainees; O&G 7 (64%) vs. GS 2 (18%), P =0.05 **(Table 2).**

For peer review only

Table 2. Laparoscopic training experience amongst O&G and GS trainees. Data is presented as frequencies (%). P values in bold indicate significant findings.

	O&G (n=23)	GS (n=22)	P
**Females**	15 (65%)	5 (13%)	**0.004**
**Males**	8 (35%)	17 (77%)	
**Juniors**	11 (48%)	11 (50%)	0.88
**Seniors**	12 (52%)	11 (50%)	
**Right handedness**	21 (91%)	19 (86%)	0.59
**Left/**ambidextrous	2 (9%)	3 (14%)	
**Played video games**	11 (48%)	8 (36%)	0.43
**Used pelvic simulator**	16 (70%)	7 (32%)	**0.01**
**Weekly**	2 (9%)	0 (0%)	
**Monthly**	3 (13%)	2 (9%)	0.32
**Less frequent**	18 (78%)	20 (91%)	
**Attended laparoscopic courses**	18 (78%)	20 (91%)	0.24
**Start of laparoscopic training:**			
**Core training**	14 (61%)	14 (67%)†	0.69
**Registrar training**	9 (39%)	7 (33%)	0.69
**Elective theatre sessions**	64 (37%)	110 (63%)	**<0.001**
**Junior**	13 (20%)	54 (49%)	**<0.001**
**Senior**	51 (80%)	56 (51%)	0.30
**Emergency theatre sessions/month**	23 (19%)	100 (81%)	**<0.001**
**Junior**	10 (43%)‡	46 (46%)	**0.003**
**Senior**	13 (57%)	54 (54%)	**<0.001**
**Type of exposure**			
**Juniors as Operator**	4 (36%)	9 (82%)	**0.03**
**Juniors as Assistant**	7 (64%)	2 (18%)	**0.03**
**Seniors as Operator**	10 (83%)	10 (91%)	0.59
**Seniors as Assistant**	2 (17%)	1 (9%)	0.59

† One junior GS trainee did not answer. ‡One junior O&G trainee did not answer.

Successful completion of tasks

Overall, O&G and GS trainees had 69 and 66 attempts at each of the three core tasks, respectively. A smaller number of attempts were successfully completed by O&G trainees in comparison to GS trainees on all three

tasks (LCN task: O&G 50 (72%) vs. GS 64 (97%), P <0.001; HEC task: O&G 54 (78%) vs. GS 64 (97%), P = 0.001; BMC task: O&G 47 (68%) vs. GS 62 (94%), P< 0.001).

Task Completion times (Speed)

There was a significant effect of specialty on completion times for LCN; F(3,33)= 6.26, P=0.005, HEC; F(3,33)=7.34, P=0.002, BMC; F(3,41)=11.6, P<0.001. Post hoc analyses showed significant differences between junior O&G and junior GS trainees only and no significant difference was found within the specialty groups, (i.e., between junior and senior trainees in either specialty). Between groups comparison showed that O&G specialty trainees were 73 seconds slower at completing LCN; O&G 166 ± 56, (139 to 193) seconds vs. GS 93 ± 21 (83 to 103) seconds, t(21)= 4.17, P<0.001, Effect size (ξ) = 0.76. O&G trainees were also significantly slower at HEC; O&G 105 ± 30 (90 to 119) seconds vs GS 67 ± 13 (60 to 73) seconds, t(25.6)=3.98, P<0.001, ξ= 0.66 and BMC task; O&G 139 ± 32 (125 to 153) seconds vs GS 100 ± 20 (92 to 109) seconds, t(43)= 4.74, P<0.001, ξ= 1.41. **(Figure 2a-c).**

Precision of movements (Accuracy)

Specialty had a significant effect on the precision of movements in LCN; F(3,33)=8.23, P=0.001, and BMC; F(3,33)=3.37, P=0.04. However, no significant difference was found in the precision of movements in HEC; F(3,33)= 0.96, P=0.43. Post hoc analysis showed that greater trainee experience did not significantly affect precision outcomes on these tasks. Therefore, the data was analysed by overall specialty. Overall, in LCN, O&G trainees found fewer targets, in the given time, than GS trainees; O&G 0.09 ± 0.04, (0.07 to 0.10) vs. GS 0.16 ± 0.03, (0.14 to 0.17), t(31.6)= 5.27, P<0.001, ξ= 0.82. In BMC, O&G trainees dropped a significantly greater number of pins than GS trainees; O&G 5.4 ± 2.3 (4.3 to 6.6) vs. GS 2.9 ± 1.7 (2.1 to 3.8), t(32.8)=3.03 P =0.005, ξ=0.53. O&G and GS trainees both dropped similar number of cylinders during HEC task; O&G 3.5 ± 2.7 (2.2 to 4.8) vs. GS 2.3 ± 1.6 (1.5 to 3.1), t(32.2)=1.23, P=0.22, ξ= 0.27. **(Figure 2 d-f).**

Suturing

The inter-rater agreement of the assessors on the suturing task was very high (Cronbach's alpha 0.98 for O&G and 0.97 for GS). One O&G trainee (4.3%) and eight GS trainees (36%) completed this task in time; P =0.007.

Number of inserted sutures and knots

Overall, O&G junior trainees were able to place fewer sutures and tie fewer intracorporeal knots than junior GS trainees (sutures: O&G 1 (1-1) vs. GS 4 (3-4), P = 0.005, Hedges *g* =0.98; knots: O&G 0 (0-1) vs. GS 2 (2-4), P = 0.005, *g*= 0.95). Senior O&G trainees tied significantly fewer knots than senior GS trainees (O&G 2.5 (1-3) vs. GS 4 (3-4), P = 0.03, *g* =0.51). However, senior trainees in O&G and GS groups placed similar number of sutures (O&G 3 (2-3) vs. GS 4 (3-4), P = 0.07, *g* =0.4).

Total suturing scores

O&G trainees had a significantly lower total suturing score than the GS trainees; F(3,33)=36.3, P <0.001). Post hoc analysis showed that junior O&G trainee's total suturing score was significantly lower than junior GS trainees; O&G 3.6 ± 2.1, (1.97 to 5.14) vs GS 14.9 ± 4.4 (11.5 to 18.3), P<0.001 and senior O&G trainees also scored lower than senior GS trainees; O&G 11.4 ± 4.4 (8.2 to 14.6) vs GS 16.8 ± 2.1 (15.2 to 18.4), P= 0.03. Senior O&G trainees had a significantly higher total suturing score than junior O&G trainees; Senior O&G 11.4 ± 4.4 (8.23 to 14.6) vs. Junior O&G 3.6 ± 2.1 (1.97 to 5.14), P = 0.004. Senior GS trainees, however,

For peer review only

scored like their junior colleagues; Senior GS 16.8 ± 2.1 (15.2 to 18.4) vs. Junior GS 14.9 ± 4.4 (11.5 to 18.3), P = 0.35 (**Figure 3).**

DISCUSSION

Principal findings

The acquisition of core laparoscopic skills depends on multiple factors including exposure to large volumes of laparoscopic procedures, [25] deliberate practice, [26] and structured simulation programs. [27] It is unknown whether the differing design of O&G and GS training leads to differential attainment of laparoscopic skills. Our study found that GS trainees performed better than O&G trainees in all tasks that measured core laparoscopic psychomotor skills**.** This may, in part, be due to the discrepancy in the volume of laparoscopic practice between the two specialties. Our baseline questionnaire showed that the average GS trainee attended the operating theatre almost three times as often as the average O&G trainee and were more likely to perform as the main operator in contrast to O&G trainees.

Our study found that increased training experience had an impact on suturing and knot tying but not on the other three core laparoscopic tasks. This may be due to the simplicity of these core tasks. Surgical skills such as navigating a camera and retracting surgical tissue are usually learnt early in the training and reach a plateau phase rather quickly. It has been confirmed that participants rapidly reached their optimal performance on simple tasks such as HEC and that despite further training no significant improvements were seen in performance. [5] Suturing, however, is regarded as a complex task and has been shown to improve with greater surgical experience. [28]

Meaning of the study: possible explanations and implications for clinicians and policymakers

Most of the emergency work in O&G relates to obstetrics and exposure to out-of-hours laparoscopic procedures is therefore limited. [29] Our study confirmed this. Overall, O&G trainees attended fewer laparoscopic theatre sessions and were less likely to be given the main operator's role than their GS counterparts. However, this difference was largely between the junior trainees only. Our baseline questionnaire showed that senior O&G trainees, in fact, attended a similar number of elective theatre sessions as the senior GS trainees and acted as the 'main operator' almost as frequently as the senior GS trainees. It appears that in O&G, theatre exposure and operative opportunities are concentrated in the latter part of the training. Psychological techniques have consistently shown that distributed practice is superior to concentrated practice and leads to the enhanced acquisition, consolidation, and retention of surgical skills. [30, 31] However, it remains unclear if the model of concentrated exposure in O&G may have contributed to the discrepancy in performance between the two specialties.

The Royal College of Obstetricians and Gynaecologists (RCOG) expects all senior (advanced) trainees to be independent in laparoscopic salpingectomy (a procedure used for removing tubal ectopic pregnancy). [3] However, senior trainees' competency in salpingectomy has been shown to vary between 32% and 89%. [32, 33] Based on feedback from O&G trainees, and documented benefits of distributed practice in learning new skills,[30, 31] introducing salpingectomy earlier in the O&G curriculum might be helpful. It may encourage hospitals to give trainees more surgical exposure from an earlier stage and trainees achieving

For peer review only

competency in this simple procedure may find it easier to learn more complex skills such as laparoscopic suturing. [5]

A greater number of O&G trainees reported using a pelvic simulator, however, only a minority reported using it frequently. Surgical skills learnt on simulators can be transferred to real patient surgery, but these benefits are mostly observed with repetitive practice and as part of a structured simulation program. [34-36] The latter is promoted as a solution for bridging the gap between required operative skills and reduced training opportunities. [37, 38] In this context the American College of Obstetricians & Gynaecologist have included a structured simulation program, as part of board certification for practice in O&G. [39]

Strengths and limitations of the study

To our knowledge, this is the first prospective study to examine trainees' laparoscopic skills in two surgical specialties who work in an anatomically similar environment. The training tools in this study were based on widely used and validated assessments, [12, 13] and our inter-observer reliability for the suturing assessments was very high. The two assessors were not involved with the individual participants' training, and they were blinded to the trainee's specialty, experience and to each other's scores.

This study was localised to the North-West region of the UK and testing it on a national level would provide more precision around the estimates of skill and enhance external validity.

The effect of training grade was only apparent in the suturing and knot tying exercise. The effect of training grade was only apparent in the suturing and knot tying exercise. In the original study validating LASTT model, the novices were predominantly students with little or no operative experience and the experts were specialists with significant experience in advance surgical procedures. So, although the original study showed significant differences between novices and experts,[12] our junior group was more experienced than their novices. Therefore, it is possible that such differences were not large enough between our groups.

Simulation practice can facilitate the acquisition of new surgical skills if used systemically and comprehensively. Only a minority of the trainees undertook regular simulation and as such it is unlikely to have had a significant effect on the study tasks. Nonetheless, the type of simulation practice in this study has not been recorded, and this is a limitation.

The sample size may appear small for an observational study. Nonetheless, there are no previous studies available examining a similar aspect, and due to the difficulties in estimating the minimum difference considered important in this context, a priori sample size estimation was not possible. Consequently, along with the mean and SD values, we also included CIs and effect sizes to enable future meta-analysis as well as inform readers of the precision and magnitude of the results.

Finally, the male: female ratio between the specialty groups was considerably different, probably reflecting the relevant population in each specialty. Although evidence points to lack of differences between male and female surgeons,[40, 41] future studies should aim to equate the participants based on sex, to alleviate any concerns around grouping male and female surgeons together.

Unanswered questions and future research

The validity of evaluating core psychomotor skills in laparoscopic surgery needs to be assessed against actual performance in the operating theatre. Our work showed that trainees with limited experience found

For peer review only - http://bmjopen.bmj.com/site/about/guidelines.xhtml

suturing (an actual surgical procedure) challenging but not the core psychomotor tasks. This implies that it is not just the mastery of core skills, but the cognitive and motor processes involved in applying these skills which may influence performance on actual surgical procedures. Therefore, future studies could look at cognitive and musculoskeletal stress amongst the two specialities and the seniority of its trainees.

Acknowledgements: We would like to thank Karl Storz, Medtronic, BSGE and BRIDGES society for their financial support with the study. We would also like to thank Mr Brice Rodriguez, Mr Kenn Ma, Dr Alison Sambrook, Mr Kenn Lim, Mr Mohamed Elsherbiny, Mr Mohamed Abdelrahman, Ms Christa Hammill, Ms Aleyamma Abraham, and Dr Nicholas Evans for their help with study protocol and conducting this study.

Funding: This study was funded by grants from the British Society of Gynaecological Endoscopy, Karl Storz and Medtronic.

Conflict of interest: None declared.

Author contributions: DAS and ZK conceived and developed the research idea. ZK, DS, DAS, CG, TJC and EH designed and implemented the study protocol. ZK, DS, AS, JEL, TJC, EH, KA, TB, CG and DAS conducted the study. TB, CG and ZK analysed the data. ZK, DS, AS, TJC, TB, CG, and DAS prepared the manuscript. All authors reviewed and approved the final manuscript.

Data statement: Data are available from the corresponding author (CG) upon reasonable request.

Ethics approval statement: Ethical approval was granted by the Faculty of Health and Medicine Research Ethics Committee at Lancaster University (FHMREC20033)

For peer review only

REFERENCES

1. Harrell AG, Heniford BT. Minimally invasive abdominal surgery: lux et veritas past, present, and future. The American Journal of Surgery. 2005;190(2):239-43.
2. Hasson H, Rotman C, Rana N, Kumari NA. Open Laparoscopy: 29- Years Experience. Obstet Gynecol. 2000;96:763-6.
3. Royal College of Obstetricians and Gynaecologists. Royal College of Obstetricians and Gynaecologists, Core Curriculum for Obstetrics and Gynaecology: RCOG; 2019 [updated June 2020. Available from: https://www.gmc-uk.org/-/media/documents/core-og-curriculum-2019-final-gmc-approved-20210504_pdf-79992893.pdf.
4. Intercollegiate surgical curriculum program (ISCP). Intercollegiate surgical curriculum program, Core Surgical Training Curriculum: ISCP; 2021 [updated Aug 2021. Available from: https://www.iscp.ac.uk/media/1326/core-surgical-training-curriculum-2021-minor-changes-for-august-2022.pdf.
5. Molinas CR, Campo R. Defining a structured training program for acquiring basic and advanced laparoscopic psychomotor skills in a simulator. Gynecological Surgery. 2010;7(4):427-35.
6. Molinas CR, Campo R. Retention of laparoscopic psychomotor skills after a structured training program depends on the quality of the training and on the complexity of the task. Gynecol Surg. 2016;13(4):395-402.
7. Royal College of Obstetricians and Gynaecologists. Royal College of Obstetricians and Gynaecologists, Training matrix London: RCOG; 2022 [Available from: https://www.rcog.org.uk/careers-and-training/starting-your-og-career/specialty-training/assessment-and-progression-through-training/training-matrix/.

8. Royal College of Obstetricians and Gynaecologists. Royal College of Obstetricians and Gynaecologists, Advanced Training in Obstetrics & Gynaecology: RCOG; 2019 [updated Aug 2019. Available from: https://www.rcog.org.uk/media/xigldqq1/advanced-training-definitive-document-2019.pdf.
9. McMurray R, Lawrence T, Afors K. Minimal access in gynaecological surgery: training the minimally invasive gynaecological surgeon. Obstetrics, Gynaecology & Reproductive Medicine. 2022;32(7):135-40.
10. Royal College of Obstetricians and Gynaecologists. Royal College of Obstetricians and Gynaecologists, Training Evaluation Feedback on Gynaecology training London: RCOG; 2021 [updated c2021. Available from: https://www.rcog.org.uk/media/vrkcsb4b/tef-2021-report-gynaecology-training.pdf.
11. Thomas C, Griffiths G, Abdelrahman T, Santos C, Lewis W. Does UK surgical training provide enough experience to meet today's training requirements? Bmj [Internet]. 2015 [cited 2022 October]; 350(h2503). Available from: https://www.bmj.com/content/350/bmj.h2503.
12. Molinas CR, De Win G, Ritter O, Keckstein J, Miserez M, Campo R. Feasibility and construct validity of a novel laparoscopic skills testing and training model. Gynecological surgery. 2008;5(4):281-90. eng.
13. Campo R, Reising C, Van Belle Y, Nassif J, O'Donovan P, Molinas CR. A valid model for testing and training laparoscopic psychomotor skills. Gynecological surgery. 2010;7(2):133-41. eng.
14. Campo R, Wattiez A, Leon De Wilde R, Molinas Sanabria CR. Training in laparoscopic surgery: From the lab to the or. Slovenian Journal of Public Health. 2012;51(4):285-98.
15. European Academy of Gynaecological Surgery. European Academy of Gynaecological Surgery, Laparoscopic Skills Training and Testing Method Europe: MiS Academy Europe; 2022 [Available from: https://europeanacademy.org/training-tools/lastt/.
16. Sleiman Z, Tanos V, Van Belle Y, Carvalho JL, Campo R. European Academy laparoscopic "Suturing Training and Testing'' significantly improves surgeons' performance. Facts Views Vis Obgyn. 2015;7(3):153-60. eng.
17. Feldman LS, Cao J, Andalib A, Fraser S, Fried GM. A method to characterize the learning curve for performance of a fundamental laparoscopic simulator task: defining "learning plateau" and "learning rate". Surgery. 2009;146(2):381-6.
18. LapPass Academy [online video]. Journal of surgical simulation. Laparoscopic Intracorporeal Suturing and Knot Tying, task 4. 1 video: 130 sec, HD, colour. UK: Journal of surgical simulation; 2020.
19. Field AP, Wilcox RR. Robust statistical methods: A primer for clinical psychology and experimental psychopathology researchers. Behav Res Ther. 2017;98:19-38.
20. Daszykowski M, Kaczmarek K, Vander Heyden Y, Walczak B. Robust statistics in data analysis — A review. Chemometrics and Intelligent Laboratory Systems. 2007;85(2):203-19.
21. Wilcox RR, Tian TS. Measuring effect size: a robust heteroscedastic approach for two or more groups. Journal of Applied Statistics. 2011;38(7):1359-68.
22. Brydges CR. Effect Size Guidelines, Sample Size Calculations, and Statistical Power in Gerontology. Innov Aging. 2019;3(4):1-8.
23. Tavakol M, Dennick R. Making sense of Cronbach's alpha. Int J Med Educ. 2011 Jun 27;2:53-5.
24. Bland JM, Altman DG. Statistics notes: Cronbach's alpha. BMJ [Internet]. 1997; 314(572).
25. Sadideen H, Alvand A, Saadeddin M, Kneebone R. Surgical experts: born or made? Int J Surg. 2013;11(9):773-8.
26. Reznick RK, MacRae H. Teaching Surgical Skills — Changes in the Wind. N Engl J Med. 2006;355(25):2664-9. eng.
27. Dawe SR, Pena GN, Windsor JA, Broeders JAJL, Cregan PC, Hewett PJ, et al. Systematic review of skills transfer after surgical simulation-based training. British Journal Of Surgery. 2014;101(9):pp1063-76. eng.
28. Train AT, Hu J, Narvaez JRF, Towle-Miller LM, Wilding GE, Cavuoto L, et al. Teaching surgery novices and trainees advanced laparoscopic suturing: a trial and tribulations. Surgical endoscopy. 2020;35(10):5816-26. eng.
29. Odejinmi F, Rizzuto I, Ballard KD. Potential barriers to the laparoscopic management of ectopic pregnancies: a regional UK study. Acta Obstet Gynecol Scand. 2010;89(10):1350-3. eng.
30. Cecilio-Fernandes D, Cnossen F, Jaarsma D, Tio RA. Avoiding Surgical Skill Decay: A Systematic Review on the Spacing of Training Sessions. J Surg Educ. 2018;75(2):471-80.
31. Gallagher AG, Jordan-Black JA, O'Sullivan GC. Prospective, randomized assessment of the acquisition, maintenance, and loss of laparoscopic skills. Ann Surg. 2012;256(2):387-93.
32. Moss EL, Bredaki FE, Jones PW, Hollingworth J, Luesley DM, Chan KK. Is gynaecological surgical training a cause for concern? A questionnaire survey of trainees and trainers. BMC Med Educ. 2011;11(1):32-. eng.

For peer review only

33. Christopoulos G, Kelly T, Lavery S, Trew G. Surgical skills of specialty trainees in emergency gynaecological laparoscopic procedures: a national UK survey. J Obstet Gynaecol. 2014;34(5):435-8.
34. Issenberg SB, McGaghie WC, Petrusa ER, Lee Gordon D, Scalese RJ. Features and uses of high-fidelity medical simulations that lead to effective learning: a BEME systematic review. Med Teach. 2005;27(1):10-28.
35. Gala R, Orejuela F, Gerten K, Lockrow E, Kilpatrick C, Chohan L, et al. Effect of validated skills simulation on operating room performance in obstetrics and gynecology residents: a randomized controlled trial. Obstet Gynecol. 2013;121(3):578-84.
36. Palter VN, Grantcharov TP. Development and validation of a comprehensive curriculum to teach an advanced minimally invasive procedure: a randomized controlled trial. Ann Surg. 2012;256(1):25-32.
37. Aggarwal R, Ward J, Balasundaram I, al. e. Proving the effectiveness of virtual reality simulation for training in laparoscopic surgery. Ann Surg. 2007;246(5):771-9.
38. Bryant-Smith A, Rymer J, Holland T, Brincat M. 'Perfect practice makes perfect': the role of laparoscopic simulation training in modern gynaecological training. The obstetrician & gynaecologist. 2020;22(1):69-74. eng.
39. American College of Obstetricians & Gynaecologists. American College of Obstetricians & Gynaecologists Washington: ACOG; 2022 [updated c2022. Available from: https://www.acog.org/education-and-events/creog/curriculum-resources/additional-curricular-resources/fundamentals-of-laparoscopic-surgery.
40. Ali A, Subhi Y, Ringsted C, Konge L. Gender differences in the acquisition of surgical skills: a systematic review. Surg Endosc. 2015;29(11):3065-73. eng.
41. Busshoff J, Datta RR, Bruns T, Kleinert R, Morgenstern B, Pfister D, et al. Gender benefit in laparoscopic surgical performance using a 3D-display system: data from a randomized cross-over trial. Surg Endosc. 2022 Jun;36(6):4376-85. PubMed PMID: 34750707. PMCID: PMC9085658. Epub 20211108.

Figure legends

Figure 1 - Outline of the training pathways in GS (a) and O&G (b). Adapted from the Royal College of Obstetricians & Gynaecologists (RCOG) (3) and intercollegiate surgical curriculum program (ISCP). (4)

Figure 2 - Time taken to complete laparoscopic tasks (a-c) and laparoscopic precision of movements by speciality (d-f). Task completion time for LCN (a), HEC (b) and BMC (c). Trainees' ability to integrate camera navigation skills with speed (d), the number of drops in HEC (e) and the number of drops in BMC (f). Data are presented as mean ± SD.

Figure 3 - Total suturing scores by trainee's experience within O&G and GS. Data are presented as mean ± SD.

For peer review only

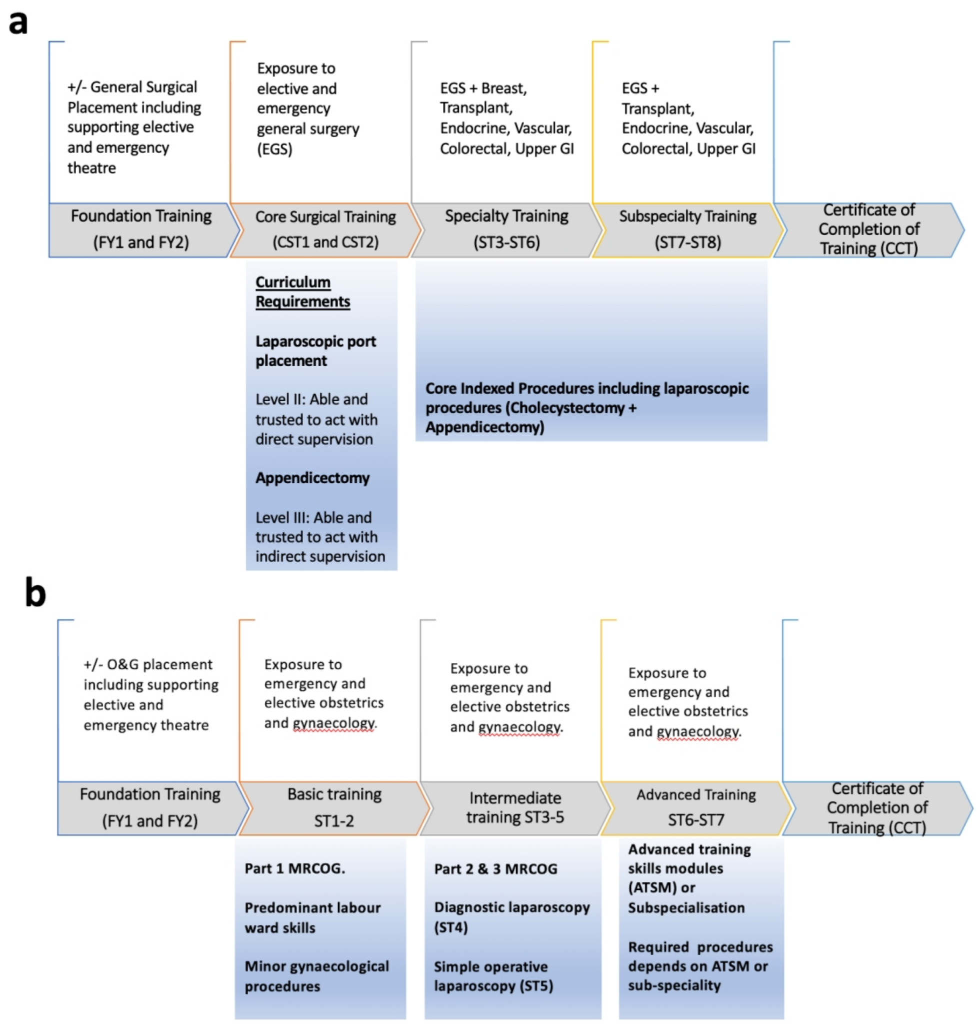

Figure 1: Outline of the training pathways in GS (a) and O&G (b). Adapted from the Royal College of Obstetricians & Gynaecologists (RCOG) (3) and intercollegiate surgical curriculum program (ISCP). (4)

140x145mm (300 x 300 DPI)

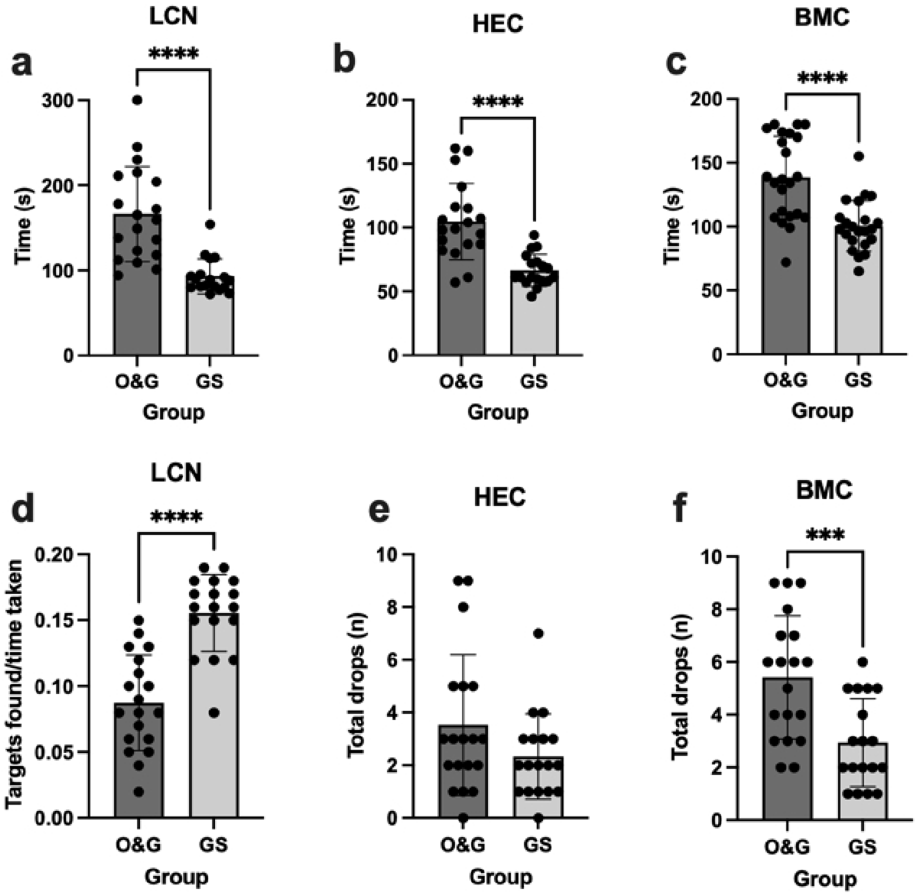

Figure 2. Time taken to complete laparoscopic tasks (a-c) and laparoscopic precision of movements by speciality (d-f). Task completion time for LCN (a), HEC (b) and BMC (c). Trainees' ability to integrate camera navigation skills with speed (d), the number of drops in HEC (e) and the number of drops in BMC (f). Data are presented as mean ± SD.

175x164mm (100 x 100 DPI)

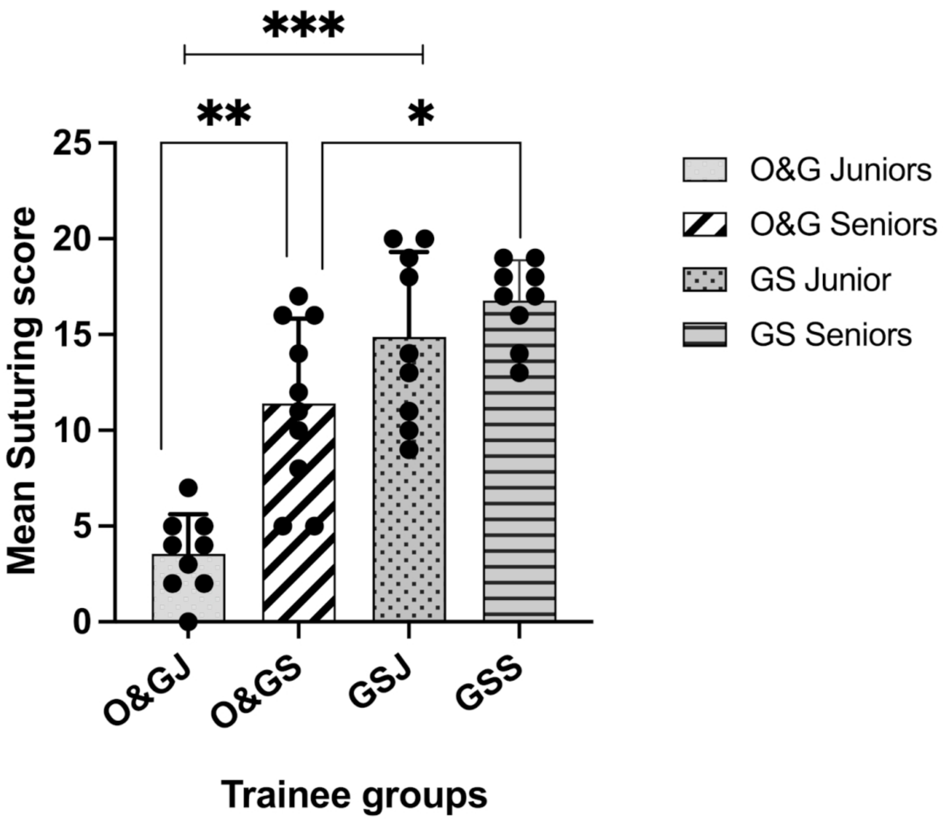

Figure 3. Total suturing scores by trainee’s experience within O&G and GS. Data are presented as mean ± SD.

128x108mm (300 x 300 DPI)

Table S1: Training matrix for O&G (adapted from RCOG). Required laparoscopic competencies are highlighted in **bold**. Competencies are signed off based on an entrustability scale* and as such no indicative numbers are included here.

	ST1	ST2	ST3	ST4	ST5	ST6	ST7
Curriculum	CiP progress appropriate to the relevant stage as per the CiP guides and entrustability levels.						
Examination	MRCOG Part 1			MRCOG Part 2 & Part 3			
At least 3 summative OSATS confirming competency by more than one assessor. At least one OSAT confirming competence should be supervised by a consultant.	Cervical smear	Caesarean section (Basic)<br><br>Non rotational assisted vaginal delivery (Ventouse & forceps)<br><br>Perineal repair<br><br>Surgical management of miscarriage/Surgical termination of pregnancy<br><br>Insertion of an Intrauterine system/intrauterine contraceptive device.<br><br>Endometrial biopsy	Manual removal of placenta<br><br>Transabdominal USS of early and late pregnancy	Hysteroscopy<br><br>**Diagnostic laparoscopy**<br><br>3rd degree perineal repair<br><br>Vulval biopsy	**Simple operative laparoscopy** (laparoscopic sterilisation or simple adnexal surgery e.g. adhesiolysis/ ovarian drilling<br><br>Caesarean section (Intermediate)<br><br>Rotational assisted vaginal delivery (any method)		Subspecialty specific competencies.<br><br>**Laparoscopic management of ectopic pregnancy**<br><br>**Ovarian cystectomy (laparoscopic & open)**<br><br>Surgical management of post partum haemorrhage

*Entrustability scale: 1= observe only, 2= direct supervision, 3= indirect supervision, 4= act independently with support, 5= act independently.

For peer review only

Table S2: Summary of required procedures in GS training. Indicative case/operative numbers are given for the specialty training phase where both the numbers and entrustability scales are used for assessment.

	CT1 Phase 1	CT2 Phase 1	ST3 Phase 2	ST4 Phase 2	ST5 Phase 2	ST6 Phase 2	ST7 Phase 3	ST8 Phase 3
Examinations		MRCS Part A<br>MRCS Part B						FRCS Part 1<br>FRCS Part 2
**Operative Requirements**<br>Level 1<br>Has observed<br>Level 2<br>Can do with assistance<br>Level 3<br>Can do whole but may need assistance<br>Level 4<br>Competent to do without assistance, including complications		Induction of pneumoperitoneum for laparoscopy with port placement (Level 2)<br>Appendicectomy (Level 3)<br>Open and close midline laparotomy incision (2)<br>Inguinal hernia repair (Level 2)<br>Primary abdominal wall hernia repair (Level 2)				Inguinal Hernia (level 4) [50 cases*]<br>Cholecystectomy (level 3) [40 cases*]<br>Segmental Colectomy (level 3) [15 cases*]<br>Emergency Laparotomy [45* cases]<br>Appendicectomy [60 cases*]		Emergency Laparotomy (Level 4) [100 cases*]<br>Appendicectomy (Level 4) [80 cases*]<br>Cholecystectomy [50 cases*] (level 4)<br>Segmental colectomy [20 cases*] (level 4)
**Other Operative Technical Skills**		Chest drain insertion (Level 3)<br>Needle biopsy including fine needle aspiration (Level 3)<br>Rigid sigmoidoscopy (Level 3)<br>Excision biopsy of benign skin or subcutaneous lesion (Level 4)						Indicative numbers and competencies for chosen specialty required. (Hepatopancreaticobiliary, Transplant, Endocrine, Colorectal, Oesophagogastric)

Reporting checklist for cross sectional study.

Based on the STROBE cross sectional guidelines.

Instructions to authors

Complete this checklist by entering the page numbers from your manuscript where readers will find each of the items listed below.

Your article may not currently address all the items on the checklist. Please modify your text to include the missing information. If you are certain that an item does not apply, please write "n/a" and provide a short explanation.

Upload your completed checklist as an extra file when you submit to a journal.

In your methods section, say that you used the STROBE cross sectionalreporting guidelines, and cite them as:

von Elm E, Altman DG, Egger M, Pocock SJ, Gotzsche PC, Vandenbroucke JP. The Strengthening the Reporting of Observational Studies in Epidemiology (STROBE) Statement: guidelines for reporting observational studies.

	Reporting Item	Page Number
**Title and abstract**		
Title	Indicate the study’s design with a commonly used term in the title or the abstract	1
Abstract	Provide in the abstract an informative and balanced summary of what was done and what was found	2
**Introduction**		
Background / rationale	Explain the scientific background and rationale for the investigation being reported	3
Objectives	State specific objectives, including any prespecified hypotheses	3
**Methods**		
Study design	Present key elements of study design early in the paper	4
Setting	Describe the setting, locations, and relevant dates, including periods of recruitment, exposure, follow-up, and data collection	4

For peer review only

Eligibility criteria	Give the eligibility criteria, and the sources and methods of selection of participants.	4
	Clearly define all outcomes, exposures, predictors, potential confounders, and effect modifiers. Give diagnostic criteria, if applicable	5-6
Data sources / measurement	For each variable of interest give sources of data and details of methods of assessment (measurement). Describe comparability of assessment methods if there is more than one group. Give information separately for for exposed and unexposed groups if applicable.	5-6
Bias	Describe any efforts to address potential sources of bias	5-6
Study size	Explain how the study size was arrived at	Sample size not possible to calculate as no previous study on this topic to base clinically significant effect size, and standard deviation measures on.
Quantitative variables	Explain how quantitative variables were handled in the analyses. If applicable, describe which groupings were chosen, and why	6
Statistical methods	Describe all statistical methods, including those used to control for confounding	7
Statistical methods	Describe any methods used to examine subgroups and interactions	7
Statistical methods	Explain how missing data were addressed	7
Statistical methods	If applicable, describe analytical methods taking account of sampling strategy	NA
Statistical methods	Describe any sensitivity analyses	NA

Results

For peer review only

Participants	Report numbers of individuals at each stage of study—eg numbers potentially eligible, examined for eligibility, confirmed eligible, included in the study, completing follow-up, and analysed. Give information separately for for exposed and unexposed groups if applicable.	7
Participants	Give reasons for non-participation at each stage	7
Participants	Consider use of a flow diagram	NA
Descriptive data	Give characteristics of study participants (eg demographic, clinical, social) and information on exposures and potential confounders. Give information separately for exposed and unexposed groups if applicable.	8
Descriptive data	Indicate number of participants with missing data for each variable of interest	7-8
Outcome data	Report numbers of outcome events or summary measures. Give information separately for exposed and unexposed groups if applicable.	8-10
Main results	Give unadjusted estimates and, if applicable, confounder-adjusted estimates and their precision (eg, 95% confidence interval). Make clear which confounders were adjusted for and why they were included	7-10
Main results	Report category boundaries when continuous variables were categorized	9-10
Main results	If relevant, consider translating estimates of relative risk into absolute risk for a meaningful time period	No risks/RR reported
Other analyses	Report other analyses done—e.g., analyses of subgroups and interactions, and sensitivity analyses	9-10
**Discussion**		
Key results	Summarise key results with reference to study objectives	11
Limitations	Discuss limitations of the study, taking into account sources of potential bias or imprecision. Discuss both direction and magnitude of any potential bias.	12

For peer review only

Interpretation	Give a cautious overall interpretation considering objectives, limitations, multiplicity of analyses, results from similar studies, and other relevant evidence.	11-12
Generalisability	Discuss the generalisability (external validity) of the study results	12
**Other Information**		
Funding	Give the source of funding and the role of the funders for the present study and, if applicable, for the original study on which the present article is based	13

None The STROBE checklist is distributed under the terms of the Creative Commons Attribution License CC-BY. This checklist can be completed online using https://www.goodreports.org/, a tool made by the EQUATOR Network in collaboration with Penelope.ai

For peer review only
